# Assessing social preferences in reimbursement negotiations for new Pharmaceuticals in Oncology: an experimental design to analyse willingness to pay and willingness to accept

**DOI:** 10.1186/s12913-021-06231-8

**Published:** 2021-03-16

**Authors:** Dominik J. Wettstein, Stefan Boes

**Affiliations:** Department of Health Sciences and Medicine, University of Lucerne, Frohburgstrasse 3, P.O. Box 4466, CH-6002 Lucerne, Switzerland

**Keywords:** Willingness to pay, Willingness to accept, QALY, Medicines regulation, Pharmaceutical policies, Value-based pricing, Behavioural economics

## Abstract

**Background:**

Price negotiations for specialty pharmaceuticals take place in a complex market setting. The determination of the added value of new treatments and the related societal willingness to pay are of increasing importance in policy reform debates. From a behavioural economics perspective, potential cognitive biases and other-regarding concerns affecting outcomes of reimbursement negotiations are of interest. An experimental setting to investigate social preferences in reimbursement negotiations for novel, oncology pharmaceuticals was used. Of interest were differences in social preferences caused by incremental changes of the patient outcome.

**Methods:**

An online experiment was conducted in two separate runs (*n* = 202, *n* = 404) on the Amazon Mechanical Turk (MTurk) platform. Populations were split into two (run one) and four (run two) equally sized treatment groups for hypothetical reimbursement decisions. Participants were randomly assigned to the role of a public price regulator for pharmaceuticals (buyer) or a representative of a pharmaceutical company (seller). In run two, role groups were further split into two different price magnitude framings (“real world” vs unconverted “real payoff” prices). Decisions had real monetary effects on other participants (in the role of premium payers or investors) and via charitable donations to a patient organisation (patient benefit).

**Results:**

56 (run one) and 59 (run two) percent of participants stated strictly monotone preferences for incremental patient benefit. The mean incremental cost-effectiveness ratio (ICER) against standard of care (SoC) was higher than the initial ICER of the SoC against no care. Regulators stated lower reservation prices in the “real world” prices group compared to their colleagues in the unconverted payoff group. No price group showed any reluctance to trade. Overall, regulators rated the relevance of the patient for their decision higher and the relevance of their own role lower compared to sellers.

**Conclusions:**

The price magnitude of current oncology treatments affects stated preferences for incremental survival, and assigned responsibilities lead to different opinions on the relevance of affected stakeholders. The design is useful to further assess effects of reimbursement negotiations on societal outcomes like affordability (cost) or availability (access) of new pharmaceuticals and test behavioural policy interventions.

**Supplementary Information:**

The online version contains supplementary material available at 10.1186/s12913-021-06231-8.

## Introduction

Price negotiations for specialty pharmaceuticals^1^ are characterized by specific features: They predominantly take place in bilateral oligopolistic or bilateral monopolistic market settings (patent-protection on the supply side and few to single payers on the demand side) [5, 9–12]. Further, the demand side is in most health systems divided in a “tripartite structure” of decider (provider), consumer (patient) and payer (insurance) [9, 11, 13, 14]. On top of that, the evaluation of the product characteristics (effectiveness, quality and safety) is inherently and increasingly complex [9, 14]. The availability of product information and thus the complexity of price negotiations has further increased with the introduction of cost-effectiveness models [5, 15, 16]. And it will further increase due to the demand to include also budget impact, burden of disease, socio-economic impact etc. in economic valuations for price determination besides cost-effectiveness [17–20]. To make a new treatment available to patients, sellers (pharmaceutical companies) and buyers (governmental regulators, health insurances or budget holders) need to agree on a reimbursable price. This holds true even in health systems with regulated pricing rules, since the market authorization holder can usually decide not to supply the respective market with the product. The determination of the added value of a new pharmaceutical and the society’s willingness to pay for an additional, quality-adjusted life year (QALY) plays an increasing role in policy reform debates [5, 16, 21–25]. Although the reform efforts are generally focused on the rules of value assessment and price determination, the process of reimbursement negotiations is itself subject to demands for reform [5, 7, 26–28].

## Background

### Perspective

The aim of our study is to broaden the debate from a behavioural perspective. The overarching ambition of our research is to bridge the gap between established health economic behavioural research on the one hand and the current discourse in research and policy on reforming pricing policies for new specialty pharmaceuticals on the other. Over the past decades, behavioural economic studies have provided insights and evidence on how individuals deviate from the standard assumption of neoclassical models [29–33]. In price negotiations in general, and reimbursement negotiations in particular, deviations due to anchoring effects, trade uncertainties, regret avoidance or concerns for others might affect negotiation outcomes [28, 31, 33, 34]. The following sections 2.3 to 2.5 summarize the relevant literature.

### Objective

We used an experimental setting to investigate social preferences in reimbursement negotiations for novel, specialty pharmaceuticals. The aspired design shall be useful to assess negotiation situations in a controlled laboratory environment. Our main interest lies on differences in stated social preferences caused by incremental changes of the patient outcome. Of further interest are differences in those preferences between different treatment groups, especially in response to the assigned roles (valuation gaps). Preferences are measured by stated reservation prices complemented by statements on the relevance of affected stakeholders. In principle, the design might be applicable to any new therapy that has a defined benefit for the patient compared to an existing alternative, measured in terms of life expectancy and quality of life. However, the study is primarily intended to contribute to the current debate on how public pricing policies for “new high-cost innovative medicines” (European Commission) can be reformed to improve “value for money” [5, 22, 33]. Since healthcare payers and regulators in OECD are faced with increasing numbers of these high-priced pharmaceuticals, which are often (predominantly) categorized as “specialty pharmaceuticals” [5–7, 15]. Global spending for specialty pharmaceuticals is projected to make up 40% in 2024 [6]. Especially new oncology treatments account for a high and increasing proportion of pharmaceutical expenditures in developed countries [7, 35]. This is likely to be accentuated as pharmaceutical companies’ oncology pipelines have grown by almost 80% in the last decade and now make up 30% of late-stage pipelines [36, 37]. Not surprisingly, regulators and payers are concerned with the “growth of pharmaceutical expenditures due to new high-cost innovative medicines” and their added value [5, 7]. For this reason, the implemented decision situation was geared towards the evaluation of specialty therapies with life-prolonging effects as in oncology.

The analysis of bargaining behaviour with offer statements and assessment of related societal effects is subject to a subsequent study [33], building on the findings below. We consider the division into two studies as necessary to clearly separate the analysis of stated preferences from the analysis of the interactive behaviour in a negotiation. It is crucial to understand whether societal effects of the reimbursement negotiation result from either contextual framing effects (of the assigned role and the setting) or from the negotiation interaction (bargaining). Our design is based on findings in the following fields of research:

### Valuation gaps: willingness to pay vs. willingness to accept

Potential endowment effects, status-quo biases, exchange asymmetries, reluctances to trade or valuation gaps in decisions have been studied in behavioural economics since the seventies (see Zeiler [34] and Korobkin [38] for an overview). A valuation gap (VG) “exists when the most a person is willing to pay for an item (WTP) is less than the least amount that same person is willing to accept (WTA) to give up the same item […]” [34]. This contradicts the neoclassic assumption that choices along the indifference curves are reversible [34, 38–41]. There have been different theories proposed to explain the phenomenon with no clear leader so far^2^ [34]. Although no “endowed” item is exchanged in our setting, role-related expectations, a different focus of the seller/buyer role [43, 49–53] or in general valuation and trade uncertainties [44, 45], regret avoidance (“bad-deal aversion”) [46–48] or moral commitments [54, 55] might cause a valuation gap [34]. Our research interest is not to contribute to the explanation efforts in general but to evaluate possible VGs for new pharmaceuticals in a plausible and relevant laboratory setting.

### Preferences for a quality adjusted life year (QALY)

There is an increasing interest in literature for preferences for a QALY, since cost-effectiveness thresholds are used in different countries to assess the value of new therapies [22, 23, 56, 57]. Several studies have already tried to assess WTP for a QALY in direct stated preference studies, mostly using some format of contingent valuation method [23, 58]. They did this either in general for a QALY of the overall population, or in specific disease areas and patient populations (e.g. diabetes) [59–61]. Our interest does not lie in nominal WTP values, but rather in differences induced the by role, the decision situation and the social effects of the decision.

### Social preferences in health economic laboratory experiments

Experimental games like the ultimatum or the dictator game have been widely used to investigate how much individuals deviate from a self-interested utility maximizing behaviour if social norms and consequences are introduced [62–64]. Findings are of special use to understand individual contributions to public goods [64]. However, there is still a surprisingly small number of experimental literature on redistribution, other-regarding or social preferences in choices of heath care provision [65–71]. Even a fewer number of these include, beside the treating physician and the patient, a third-party payer [67, 72]. The laboratory experiments on health insurance choices on the other side mostly focus on individual risk preferences (e.g. [73]), moral hazard (e.g. [74]) or willingness to pool (e.g. [75]), but not on social preferences for incremental (insurable) QALYs. No experimental evidence on pricing negotiations for pharmaceuticals was found in our systematic literature review [76].

Our experimental design integrates the three research interests to assess exchange asymmetries (WTP vs. WTA), QALY preferences (WTP for health) and social preferences (WTA or WTP reflecting distributional effects) [33]. This is necessary to assess preferences in reimbursement negotiations for new health interventions, particularly for specialty pharmaceuticals.

## Methods

### Design^3^

#### Overview design

Participants were randomly assigned into two (run one) and four (run two) treatment groups of equal size. Populations were split to play either in the role of a health minister (regulator) or as a representative of a pharmaceutical company (seller). In run two the role groups were further split into two different price magnitude framings. One with fictive “real world” prices (100 k$ group) vs one with prices at the “real payoff” level (1$ group). While final payoffs were equal in both groups, prices in the “fictive price” group were converted for the game to 100,000$ = 1USD (in the following, prices for the 100 k$ group are used for simplification). Participants were informed that their decisions would have real monetary consequences on other participants (payoffs for their passive role as premium payers or investors) and in form of charitable donations to a patient association (proxy for patient benefit).^4^ See Table 1 below for an overview of the runs and treatment groups.

**Table 1:** Design of the experiment: number of subjects randomly assigned to treatment groups

Game (round)	Intervention	Group (n), decision					
		Run 1		Run 2			
	Price magnitude^a^	100,000 $ = 1 US$		100,000 $ = 1 US$		1 $ = 1 US$	
		Group 1	Group 2	Group 3	Group 4	Group 5	Group 6
Game 1 (round 1 to 5)	Role	Regulator (100), WTP	Seller (101), WTA	Regulator (97), WTP	Seller (101), WTA	Regulator (105), WTP	Seller (101), WTA
		*Role reversal (within-subject)*		*No role reversal*			
Game 2 (round 1 to 5)	Role	Seller (100), WTA	Regulator (101), WTP	*Price offers (not covered in this publication)*			

In both runs, participants played the same five rounds in two consecutive games. All relevant information about the consequences of the negotiation were provided before the first game. Participants did not know which rounds would be relevant for final payoffs, nor that the game would be repeated after five rounds

Run one: four rounds voluntary training before first five rounds (initial role only) taken by *n* = 92 participants for group 1 (Regulator) and *n* = 93 for group 2 (Seller). One participant removed in run one due to age not meeting inclusion criteria. No participants removed for run two

Run two: New subjects recruited. Adjustments: introductory training mandatory for all participants; additional bonus for deciders if price offer facilitates an agreement (game 2, not covered in this publication); additional message displayed below decision table if price entered would lead to a not strictly monotone preference statement (“you did not increase your price while the patient outcome of the product increased. Does this truly reflect your preference?”); four comprehension questions (three to pass, one to filter) and one attention screening question in between to identify inattentive responders

Table adapted from Wettstein/Boes 2020 [33]

*WTP* willingness to pay, *WTA* willingness to accept

^a^Game currency converted to real payoff at the end of the experiment

#### Overview setting (contextual framing^5^)

**Fig. 1:**
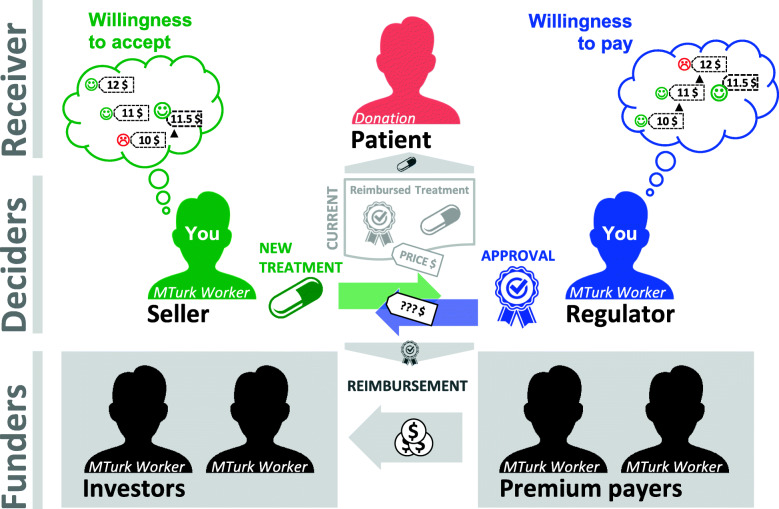
Design of the experiment: roles and tasks. In the experiment, available premiums were directly redistributed from the premium payers to the investors, depending on the pricing decision of the deciders. In a real setting, the premiums would contribute to a health fund or plan which would pay the treatment, usually via a health care provider, to the local market authorization holder. The final return on investment for the investor would have to reflect in addition research, development and operational cost. Figure adapted from the pictures displayed in the experimental survey used in this and the subsequent study [33]. The experimental surveys are available in Additional file 3 and Additional file 4. The original illustrations are the authors’ own creations

The reimbursement situation involved a hypothetical country with seven citizens, represented by five different types of stakeholders (Fig. 1). A single patient was chosen for comparability with existing experiments on physician treatment situations (e.g. [66–70]) while the funders were represented by a group of two each. A small patient number is also a plausible framing considering the increasing proportion of small and orphan indications in specialty treatment areas in general and oncology particular, for which high-priced pharmaceuticals are offered [5, 6, 25, 78, 79]. Regarding the number of affected funders the setting builds on the design of Kesternich et al. [67] as well as on Schumacher et al. who found that deciders in a distributional decision situation “attach the same weights to small and large groups“ of payers [80].

The role of each stakeholder was explained as follows (scenario displayed in Fig. 1):

The patient is suffering from a deadly blood cancer and is under treatment with an existing therapy (current standard of care, SoC), a pharmaceutical product with a known benefit to the patient.

The regulator is in charge of regulating prices for pharmaceuticals, eligible for payment by the public health insurance.

The seller represents an international pharmaceutical company that developed a new product to treat the patient. He is in charge of negotiating an officially reimbursed price for the product.

The premium payers finance the public health insurance. The collected premiums are the only financial source to pay for the patient’s treatment. Only treatments approved by the government for reimbursement are covered. If health expenditures are lower than the actual premiums, payers can accumulate savings. If the expenditures are higher, they will have to eat up their savings.

The investors have invested their savings in the past into the pharmaceutical company. They expect a return on their investment, which compensates them for the additional risk they took, compared to a “risk-free” investment in a government bond for example.

#### Overview decision situation

The reimbursement process was explained as follows:

The seller offers a new treatment at a proposed price. If the regulator considers the price to be too high, he will refuse approval of the product. Vice versa, if the seller receives a (simultaneous) counterproposal considered too low, he will not introduce the product in this market. If both parties agree, the patient will get access to the new treatment and the related benefits (life expectancy in months m, quality of life in percent q). The two investors will in consequence receive a revenue (price divided by two) and the two payers will pay the cost (leaving them an equal share of the difference between premiums and price). The patient’s quality of life is equal to his work ability. The income for a healthy person (q = 100%) is 10,000$ per month or 120,000$ per year. This gives the patient a benefit (total income) of q × 10,000$ × m. See Table 2 below for an overview.

**Table 2:** Design of the experiment: parameters per role and round

			Deciders		Receiver			Funders	
			Regulator	Seller	Patient			Payers	Investors
Treatment option	Round	Reservation price^a^	Benefit^a,b^	Benefit^a,b^	Survival (m)	Quality of Life (q)	Benefit^a^	Benefit^a^	Benefit^a^
No treatment					0	50%	0		
Standard treatment (SoC)	0	50	120	120	5	50%	25	240 - x	x
New treatment option	1	x	120	120	8	50%	40	240 - x	x
	2	x	120	120	10	50%	50	240 - x	x
	3	x	120	120	12	50%	60	240 - x	x
	4	x	120	120	15	50%	75	240 - x	x
	5	x	120	120	17	50%	85	240 - x	x

Table adapted from Wettstein/Boes 2020 [33]

*SoC* standard of care (status quo), *m* survival in months, *q* quality of life on a scale of 1–100%

^a^for groups 1 to 4, amounts displayed in thousands $ (converted 100,000 $ = 1 US$ at end of the experiment); for groups 5 to 6, amounts divided by 100 and displayed in $ (converted 1 $ = 1 US$ at end of the experiment)

^b^additional bonus for participating in training in run one and for successful offer in run two (game not covered in this publication)

The regulator and the seller are both employed and receive a fixed salary. An agreement is possible with a reservation price (x_S_) of the seller below or equal to the reservation price (x_R_) of the regulator. The price decision is constrained to a range between 50,000$ (which is the price of the current SoC) and 500,000$.

Price parameters were chosen to meet the following conditions:

1. Fictive prices had to be in the range of new, specialty pharmaceuticals in oncology, looking at yearly treatment cost [7, 36, 78, 81].
2. The fictive yearly incomes had to be in a plausible range for the framed responsibility (average compensation of middle managers in public services in OECD [82]).
3. Fictive price and salary numbers, as well as real payoffs, had to be easy to use in explanations and calculations, since the topic is complex for an experiment.
4. Conversion between fictive and real payoff amounts had to be simple and comparable while fulfilling at the same the time minimum wage requirement of the MTurk platform.

### Hypotheses

A “robust finding” from past laboratory experiments in economics is that “individuals take into account the welfare of all parties and have a preference for efficient outcomes” [33, 62, 63, 80, 83, 84] and that “non-selfish preferences are the rule rather than the exception” [85]. Building on these research results, the model used in this study was based on a simple CES^6^-function, where a rational decider (regulator, seller) should maximize his or her social utility considering the utility of the other involved stakeholders besides his or her own. For details on the underlying model, see Additional file 2.

- *Null hypothesis 1: reservation prices (x) are randomly distributed between 50,000$ and 500,000$ in each round (r) and do not differ between rounds with incremental patient benefit (E[X*_*r*_*] = 275,000$).*
  The first hypothesis tests whether the mean participant responds to the incremental patient benefit between rounds and, if not, whether the mean price is equal to the expected value of a random distribution. The null hypothesis assumes a rational decider with no other-regarding preferences.
- *Null hypothesis 2: reservation prices equal X*_*r*_ *= 120,000$ in each round.*
  The second hypothesis tests whether the mean participant responds to the incremental patient benefit and, if not, whether the mean price leads to an equal distribution of payoffs between the two passive funders. The null hypothesis is based on the assumption that a rational decider does not care about incremental patient benefit, but is averse to inequality between funders.^7^
- *Null hypothesis 3: the incremental cost-effectiveness ratio (ICER) compared to the given standard of care (SoC) equals 20,000$ for every round.*
  The third hypothesis tests whether the mean participant applies a simplifying heuristic to reduce the complexity of the decision situation instead of reflecting on the reservation price. To do this, the player simply divides the costs of the given standard treatment of 50,000$ by its effect of 5 months survival and increases the price for a new therapy option by 10,000$ for every additional month of survival. Reflecting quality of life this corresponds to an ICER of 20,000$ per quality adjusted life month (QALM, see Additional file 2). The null hypothesis assumes a bounded-rational participant with efficiency concerns^8^
- *Null hypothesis 4: reservation prices converted to payoff-magnitude do not differ between price groups for any round.*
  The fourth hypothesis tests whether the conversion (framing) of the price range for the 100 k$ group to the magnitude of real world list prices for pharmaceuticals in oncology has an impact on the stated reservation prices (converted back to the level of the final payoffs). The null hypothesis assumes a rational decider who is only interested in the actual payoffs to affected stakeholders at the end of the experiment.
- *Null hypothesis 5: reservation prices do not differ between role groups for any round.*
  The fifth hypothesis tests for potential valuation differences (WTP ≠ WTA) with special interest in valuation gaps (WTP < WTA, representing a reluctance to trade). The null hypothesis assumes a rational decider for whom the reservation price is independent of the assigned role.^9^
- *Null hypothesis 6: ranking of the stakeholders’ relevance is identical for all groups.*
  The sixth hypothesis tests for possible differences in the weighting of the stakeholders involved, which is determined after the pricing decisions have been made. The null hypothesis assumes a rational decider for whom the weighting of affected stakeholders is independent of the assigned role and who is only interested in the actual payoffs to affected stakeholders at the end of the experiment.

### Implementation

An online experiment was conducted in two separate runs (*n* = 202, *n* = 404) on the Amazon Mechanical Turk (MTurk) platform.^10^ The platform allows to conduct an experiment at a narrow timeframe, easily process monetary payoffs and has known reliability (see 3.5). Players had to use a slider to submit their prices.^11^ Regulators were instructed to state the “absolute maximum price”, which they would “still consider reasonable and fair for the new product”, while sellers were instructed to state their “absolute minimum price”. Both roles received an identical introduction, apart from their role framing. Participants played the same five rounds to state their reservation prices in both runs. In run one, participants played the reservation price game twice, switching to the opposite role for the second game. Participants had to rank all involved stakeholders after each game in terms of their relevance for the decision. An introductory training was voluntary in run one and mandatory in run two. To control for understanding of the setting, participants had to answer four comprehension questions in run two, three of them forcing correct answers, one without feedback (effect of incremental survival on patient benefit). To test for insufficient attention, an instructional manipulation check was implemented in run two [87, 88].

We used the strategy method [89] to trigger the financial consequences^12^: Participants were informed at the beginning of the experiment that all games would be potentially relevant for the social payoffs. After the experiment, in run one, one out of ten rounds was randomly selected for each participant and implemented for all four passive stakeholders randomly assigned. For run two, all rounds of game two were implemented. Participants were given full information on how any potential price would affect all stakeholders in each round, provided with a table that dynamically displayed all social payoffs for each slider position (see Additional file 1). Further they were shown their decisions from the previous rounds for comparison. In run two a message was displayed, if the entered price was equal or lower than in the previous round. However, participants were allowed to ignore this and submit any price in the given range.

For details on the displayed screens and the payoffs at the end of the experiment, see Additional file 1. The full experimental instructions used in this and the subsequent study [33] are available in Additional file 3 (run one) and Additional file 4 (run two).

### Preference elicitation method

While single binary-choice surveys are currently the preferred format for public goods preference elicitations, alternative formats of contingent valuation can be necessary and incentive-compatible under certain conditions [91, 92]. The implementation of a dichotomous choice format or a bidding procedure would neither have been appropriate nor implementable in the present setting.^13^ The contingent valuation method was used in a direct matching format instead, in line with comparable laboratory experiments on social preferences in health care [66–70]. The decision situation, while not dichotomous, was discrete in the sense that participants were forced to choose their reservation price from a defined price range with a finite number of increments.^14^ For each of these price increments all monetary consequences for every stakeholder involved were displayed dynamically in the decision table (see Figure 12 in Additional file 1). The table further displayed the change for all stakeholders for any potential price decision versus the status quo (baseline).^15^ In order to ensure incentive compatibility, three known sources of bias had to be addressed: bias due to strategic behaviour (i), bias due to lack of understanding of the monetary consequences (ii), bias due to a lack of engagement in the game or indifference to the social payments (iii) [91, 97, 98].

(i) As per the model presented, a rational player had no monetary incentive to over- or understate preference statements. Since this would have led to a suboptimal distribution of payoffs between payers and investors after the experiment, without benefiting the patient or the negotiators themselves.
(ii) To avoid bias due to over- or underestimation of cost and benefit of the decision, participants were provided with the above mentioned decision table consisting of all relevant monetary consequences for any potential price (multi-attribute alternatives). In addition, performance in the introductory training was surveyed to control the results for proper understanding (see 3.3.).
(iii) In online experiments in general, it is important to distinguish participants who understand and follow the instructions from those who focus on completing the survey as quickly as possible to minimise the time spent [87, 88]. In order to do this, the populations were divided after completion of the experiment into those with strictly monotone preferences, as per introduction and training, and those without. The grouping was tested for differences in time spent on the experiment, performance on the training and performance on the attention screener (see 3.3.). Results were reported separately for the two groups.

### Study population

No expert population was deliberately selected for the experiment. The expert surveys conducted in the recent past with competent authorities indicate how small the available pool might be (see e.g. [8, 26, 99, 100]). More importantly, the individual experience of professionals due to country specifics, focus in therapy area etc. would not be controllable. Also, the connection to the behavioural economic evidence from laboratory experiments (valuation gaps, social preferences) introduced above would hardly be plausible, nor would the implementation of monetary incentives. Instead the MTurk population with studied characteristics was used [101]. In past years, convincing evidence has been generated to support the reliability of MTurk results compared to laboratory and field experiments in general and the usefulness for assessing social preferences specifically [87, 102–109]. To avoid bias due to health system-related differences, participants had to be US resident (the vast majority of MTurk workers logs in from the US with over 70% [101]), at least 18 years of age. The target population was deliberately not focused on the health sector, but relevant professional experience was surveyed (health service providers, public authorities, pharmaceutical companies, health insurance companies, etc.). Additional demographic information, risk behaviour and health experience were surveyed at the end of the experiment. The respective variables were used to control the results.

### Statistical methods

We used Chi-square, Cramer’s V, Fisher’s exact test, independent and paired sample t-tests, independent Mann-Whitney U and Wilcoxon matched-pairs signed-rank tests, as well as hierarchical linear regressions (two-level random effects model).

## Results

### Reservation prices

Prices in the 100 k$ groups were converted in the following to the real payoff magnitude (at the end of the experiment) for comparison with the 1$ groups.

Average prices increased overall with each consecutive round and incremental patient benefit in all three reservation price games (*p* < 0.01, except first increment run one *p* < 0.05). All mean prices were different from the expected value of a random distribution (null hypothesis 1 rejected at p < 0.01, except first round in run one *p* < 0.05). Furthermore, for all rounds they were higher than 1.2$, which would have distributed assets evenly between payers and investors (null hypothesis 2 rejected at *p* < 0.01).

Whereas mean reservation prices suggested strictly monotone preferences for incremental patient benefit overall, this was only true for 56 and 57 (run one) respectively 59% (run two) of the respondents. While the majority of the participants submitted four consecutive times a higher price, 14 respectively 17% stated once or twice non-strict preferences for incremental patient benefit in run two. The group of participants with strictly monotone preferences had significantly lower mean reservation prices compared to the other participants (*p* < 0.01, except run one two rounds *p* < 0.05, one round not significant). For the latter group, the difference to the expected value of a random distribution was still significant (*p* < 0.01) for run one and the first three rounds in run two (p < 0.05). However, the significantly higher variances in this preference group (*p* < 0.01) raise the question whether these respondents focused on finishing the experiment as fast as possible to collect the fixed salary, rather than on deciding on their reservation price as per instruction. These participants spent significantly less time on the experiment in both runs (p < 0.01). They also did significantly worse in answering comprehension question four and detecting the attention screening question (*p* < 0.01) with 81% missing the screener.

The reservation prices per round were not different between the two runs for participants with monotone preferences, while prices for participants with non-monotone preferences were not comparable (*p* < 0.01, first round *p* < 0.05). Unless stated otherwise the following results will focus on the monotone preference group in run two.

### Cost-effectiveness as simplifying heuristic

**Fig. 2:**
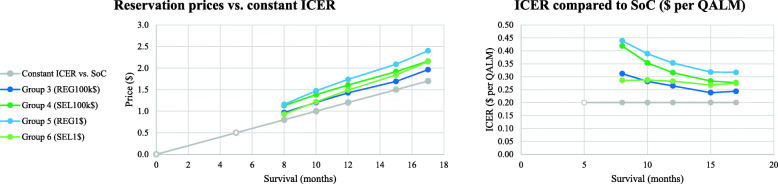
Cost-effectiveness of reservation prices submitted (run two, monotone preferences). REG, regulator; SEL, seller; SoC, standard of care; QoL, quality of life; pref., preferences; ICER, incremental cost-effectiveness ratio; QALM, quality adjusted life month

The cost-effective prices were the most frequent answers looking at the modal value (except for one round in run one with two equal modes). However, the mean ICER per QALM versus SoC was significantly higher than 0.2$ overall (null hypothesis 3 rejected at *p* < 0.01, Fig. 2) and for all treatment groups (*p* < 0.01, except group three *p* < 0.05 for the first three rounds, round four not significant). The consecutive ICERs between rounds decreased in the monotone group from round one to three, increasing again in round five (*p* < 0.01, in round four not significant). One explanation could be that the mean participant did not adjust to the higher incremental survival in round four (+ 3 m) compared to the rounds two, three and five (+ 2 m).

### Valuation differences due to the price magnitude

**Fig. 3:**
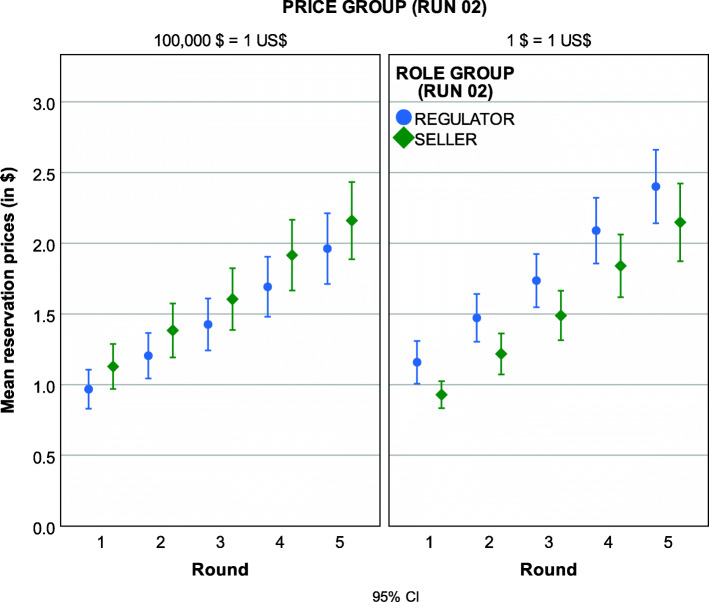
Reservation prices, means per price and role group (run two, monotone preferences). CI, confidence intervals

Mean reservation prices between the two price groups did not differ overall (*p* > 0.05). They did, however, looking at WTP and WTA separately (Fig. 3). Regulators stated lower prices in the 100 k$ group compared to their colleagues in the 1$ group in round two to five (*p* < 0.05). While sellers of the 100 k$ group had higher reservation prices, but only in the first round (*p* < 0.05). If we filter results further for participants who detected the attention screener, the effect becomes significant for all rounds in the regulator group (null hypothesis 4 rejected at *p* < 0.01) and no round in the seller group.

### Role-related differences

We tested two potential valuation gaps, one between subjects (null hypothesis 5a) and one within subjects (null hypothesis 5b, run one). The paired t-test did not differ for the same round between games for none of the rounds in run one, hence we cannot assume valuation gaps within subjects (null hypothesis 5b not rejected with *p* > 0.05).

Looking at the mean reservation prices in run two (Fig. 3), we found a potential valuation gap between regulators and sellers in the 100 k$ group since WTP < WTA for all rounds. This would represent a reluctance to trade. However, this gap was not significant overall (null hypothesis 5a not rejected with *p* > 0.05). In the 1$ price group, we found a negative gap (“preference range”) with WTP > WTA for all rounds. This gap was significant for round one and two (null hypothesis 5a rejected at *p* < 0.05). The Mann-Whitney test confirmed no role-related differences for the 100 k$ group, while distributions were different for two rounds in the 1$ group (*p* < 0.05). The later effect vanished if controlled with the attention screener.

### Ranking of stakeholders’ relevance

In the first game, the patient was for the majority of participants the most important stakeholder (both runs), followed by the own role (run two *p* < 0.01). After the role switch in run one, the own role caught up to the patient, rated together more often first, compared to the other stakeholders (*p* < 0.01). The ranking of the most relevant stakeholder differed between preference groups in both runs and all games (*p* < 0.01, *p* < 0.05 for the second game in run one) with a higher rating of the patient’s relevance by participants with strict monotone preferences.

Looking at the full stakeholder ranking (not only number ones), participants with monotone preferences in run two ranked their negotiation partner third, after the patient (first *p* < 0.01) and the own role (second *p* < 0.01), before investors and premium payers (*p* < 0.05, Wilcoxon test).

Interestingly, participants in run two, who rated the patient as number one, submitted lower prices in all rounds compared to those who rated themselves first (*p* < 0.01). Controlling for preferences, the effect is still significant in all rounds for participants with strict monotone preferences (*p* < 0.05), but only for the first round in the non-monotone group (*p* < 0.05).

The ranking was further significantly different between the two roles in the group with monotone preferences in both runs (null hypothesis 6a rejected at *p* < 0.01, *p* < 0.5 for game one, run one) with regulators rating the patient more often as most important. The ranking of the own role as most important on the other side was more frequent in sellers. The ranking did not differ between the two price groups (null hypothesis 6b not rejected at *p* > 0.01).

### Full regression model

A linear regression (two-level random effects model) over all 1190 reservation prices of the monotone group in run two was performed (Table 3). The results confirm the findings above in general, particularly increasing preferences for incremental patient benefit (*p* < 0.01). The regression confirms the finding regarding price magnitude (4.3 above) with lower price statements for regulators in the 100 k$ group compared to their colleagues in the 1$ group (*p* < 0.01 with model 2, *p* < 0.05 with model 1 and 3). In addition to the above findings, prices increased less in the $100 k group (*p* < 0.05), accompanied by a higher average price across all rounds (*p* < 0.01). The regression further confirms the finding regarding role-related differences (4.4 above); sellers had lower prices than regulators in the 1$ group (*p* < 0.05), while prices did not differ between roles in the 100 k group (*p* > 0.05). In contrast to the results above, controlling for sufficient attention (screener) did not neutralize the role-related difference in the $1 group. The regression finally confirms the correlation between patient orientation and reservation price (4.5 above). With the refinement that players who rated the patient as the most important stakeholder did not generally have lower reservation prices than players who prioritised their own role (*p* > 0.05). Rather, the prices of the former rose less than those of the latter (*p* < 0.05). However, the effect is weak and limited to the 100 k group. The extended model 3 also shows that the effect seems to be driven by self-oriented players and differs between roles, with higher price sensitivity for sellers who prioritise their own role over the patient (*p* < 0.05). The attention screener explained price differences overall (*p* < 0.01) and in the 1$ prices group (*p* < 0.05), while the comprehension question had no additional impact (*p* > 0.05).

**Table 3:** Linear regression for reservation prices in run two (two-level random effects model)

	Overall										Price groups											
Model	*Model 0*		*Model 1*		*Model 2*		*Model 3*		*Model 3b*		*Model 1*				*Model 2*				*Model 3*			
Price magnitude^c^											100 k$		1$		100 k$		1$		100 k$		1$	
Estimates of fixed effects (explanatory)	Est.	sig.	Est.	sig.	Est.	sig.	Est.	sig.	Est.	sig.	Est.	sig.	Est.	sig.	Est.	sig.	Est.	sig.	Est.	sig.	Est.	sig.
Intercept	1.60	^a^	0.41	–	− 0.01	–	− 0.15	–	− 0.15	–	0.59	–	0.58	–	0.40	–	0.24	–	− 0.01	–	0.55	–
Survival			0.13	^a^	0.15	^a^	0.15	^a^	0.15	^a^	0.11	^a^	0.13	^a^	0.13	^a^	0.14	^a^	0.13	^a^	0.14	^a^
ROLE GROUP [=0]^d^			0.26	^b^	0.26	^b^	0.22	–	0.22	–	−0.11	–	0.28	^b^	−0.23	–	0.31	^b^	−0.22	–	0.30	^b^
PRICE GROUP [=0]			0.37	^a^	0.40	^a^	0.36	^a^	0.36	^a^												
RANK Patient			−0.01	–							−0.02	–	0.03	–								
RANK Premium payers			−0.05	–	−0.01	–	−0.00	–	− 0.00	–	−0.06	–	− 0.05	–	−0.01	–	− 0.01	–	−0.00	–	− 0.03	–
RANK Investors			−0.06	–	−0.04	–	− 0.03	–	−0.04	–	− 0.06	–	−0.06	–	− 0.03	–	−0.07	–	− 0.01	–	−0.10	–
RANK Own role			−0.05	–							0.02	–	−0.11	–								
RANK Negotiation partner			0.00	–	0.08	–	0.07	–	0.07	–	0.00	–	0.00	–	0.10	–	0.05	–	0.10	–	0.01	–
Stakeholder No1 (Patient = 0, self = 1) [=0]					−0.12	–	−0.04	–	−0.04	–					0.02	–	−0.29	–	0.08	–	−0.21	–
ROLE GROUP [=0] × PRICE GROUP [=0]			−0.35	^b^	−0.48	^a^	−0.39	^b^	−0.39	^b^												
ROLE GROUP [=0] × Survival			−0.00	–	0.00	–	0.00	–	−0.01	–	− 0.01	–	0.00	–	− 0.00	–	0.01	–	− 0.00	–	0.01	–
PRICE GROUP [=0] × Survival			−0.02	^b^	−0.02	^b^	−0.02	^b^	−0.02	^b^												
Stakeholder No1 [=0] × Survival					−0.02	^b^	−0.02	^b^							−0.03	^b^	−0.01	–	−0.03	^b^	−0.01	–
Stakeholder No1 [=0] × ROLE [=0] × Survival									−0.01	–												
Stakeholder No1 [=1] × ROLE [=0] × Survival									−0.03	^b^												
Estimates of fixed effects (control variables)																						
Performance: attention screening question							0.26	^a^	0.26	^a^									0.20	–	0.31	^b^
Performance: comprehension question 4							−0.01	–	−0.01	–									0.07	–	−0.03	–
Health experience & risk behaviour							*X*		*X*										*X*		*X*	
Demographics			*X*		*X*		*X*		*X*		*X*		*X*		*X*		*X*		*X*		*X*	
Estimates of covariance parameters																						
Residual	0.29	^a^	0.01	^a^	0.01	^a^	0.01	^a^	0.01	^a^	0.01	^a^	0.01	^a^	0.01	^a^	0.01	^a^	0.01	^a^	0.01	^a^
Intercept [subject]^e^, Variance	0.50	^a^	0.39	^a^	0.36	^a^	0.33	^a^	0.33	^a^	0.38	^a^	0.37	^a^	0.33	^a^	0.36	^a^	0.30	^a^	0.33	^a^
Survival [subject], Variance			0.01	^a^	0.00	^a^	0.00	^a^	0.00	^a^	0.00	^a^	0.01	^a^	0.00	^a^	0.01	^a^	0.00	^a^	0.01	^a^
Model summary																						
Observations: price statements (participants)	1190	(238)	1190	(238)	1020	(204)	1020	(204)	1020	(204)	610	(122)	580	(116)	525	(105)	495	(99)	525	(105)	495	(99)
Intraclass correlation (ICC)^e^	0.63		*0.97*		*0.97*		*0.96*		*0.96*		*0.97*		*0.96*		*0.97*		*0.96*		*0.97*		*0.96*	
Proportional reduction of error term (R2)^f^			0.49		0.53		0.56		0.56		0.50		0.51		0.56		0.51		0.61		0.56	
R2 level 1^g^			0.96		0.96		0.96		0.96		0.96		0.95		0.96		0.95		0.96		0.95	
R2 level 2^h^			0.22		0.28		0.33		0.33		0.23		0.26		0.33		0.26		0.40		0.34	
Model description																						
*preferences*			*X*		*X*		*X*		*X*		*X*		*X*		*X*		*X*		*X*		*X*	
*patient orientation*					*X*		*X*		*X*						*X*		*X*		*X*		*X*	
*performance & personality*							*X*		*X*										*X*		*X*	

Depending variable: reservation prices 1US$; only monotone preferences included

Survival (in months); PRICE GROUP (0, 100 k$; 1 1$); ROLE GROUP (0, REGULATOR; 1, SELLER); Comprehension question (0, wrong; 1, correct); Attention screener (0, wrong; 1, correct); RANK Stakeholder (1, highest … 5, lowest rank); Stakeholder No1 (0, Patient; 1, Own role; excluded if else); Health experience (5 questions on own health history and history with family or friends); Risk behaviour (4 questions on economic, 4 questions on social risk behaviour)

^a^significant at 1% level; ^b^significant at 5% level

^c^Game currency converted to real payoff at the end of the experiment

^d^Variable [=1] not displayed, if redundant to [=0];

^e^Due to the significant intercept at level 2 (subject or participant), the two-level model is indicated. The ICC shows for the null model that 63% of variance is explained by differences between subjects (level 2) and 37% by differences within subjects (level 1). For the other models the ICC explains the remaining variance

^f^E.g. for Model 1, the proportional reduction of the predictive error is 49%

^g^E.g. for Model 1, the predictive power is 96% higher with the level 1 predictor compared to the null model

^h^E.g. for Model 1, the predictive power is 22% higher with the level 2 predictor compared to the null model

## Discussion

The objective of the present study was to assess differences in stated social preferences caused by incremental changes of the patient outcome in a controlled experiment. Of special interest were differences between assigned roles (valuation gaps) and the price magnitudes. The design proposed led to meaningful results with a majority of participants stating strictly monotone preferences for incremental survival of the patient. No systematic reluctance to trade (valuation gaps) was found which would prevent an agreement (patient access) between mean negotiators in a subsequent trade interaction. However, the found impact of the assigned role on the stated relevance of the stakeholders involved could have a potential influence in a subsequent price negotiation.^16^ From a methodological point of view, the valuation differences found in the $1 group require further investigation and potential improvement of the design. The preference range could be due to the complexity of the decision situation in general, which should, however, affect both price groups equally.

The price magnitude of current oncology treatments seems to affect stated preferences for incremental survival. Differences found between price groups call for further investigation of different price framings. It cannot be completely ruled out that the given (limited) price range had an influence on the stated preferences. Such an anchoring effect should be the same across price groups, since the real payoffs were also the same. However, the mean differences found could vary just as well with a larger or smaller given price range. This might be important, especially since anchoring in real reimbursement negotiations, driven by the first offer (but also by the standard of care), is likely to play a relevant role for negotiation outcomes [28].

The finding that participants did not apply a stable cost-effectiveness rule as a simplifying heuristic could be a promising starting point for further research. To address the need mentioned above to understand the influence of the complexity of the decision situation, as well as the impact of nominal price anchors. The incremental cost-effectiveness ratio (ICER) reflects financial consequences in relation to the resulting patient benefit. The introduction of an ICER-calculator in the pricing decision could help to reduce potential behavioural biases. The available evidence from laboratory experiments shows that participants do show efficiency concern and use or are susceptible to simplifying heuristics [67, 73, 83, 86]. At the same time, investigating the use of cost-effectiveness in reimbursement negotiations would be a plausible bridge to the real world setting and related reform debates. The key figure has been found to be the most important predictor for NICE decisions for example [27, 112]. While other countries such as France, Germany, Sweden or Italy also require or allow elements of economic evaluation (like cost-effectiveness or cost-benefit) to directly or indirectly influence their reimbursement decisions, the evidence for the effectiveness of such measures is generally still scarce [18, 76, 113, 114]. The design presented could be enhanced by introducing an ICER tool to test its potential impact on reservation prices and value-based price negotiations.

Our subsequent study on the design presented will complete the negotiation setting with binding price offers to an assigned negotiation partner [33]. This is important, since the effective bargaining behaviour is expected to have an additional impact on prices offered, compared to the players’ private reservation price. The analysis of the related negotiation outcome will further allow us to analyse and discuss societal effects regarding cost and availability of new pharmaceuticals. A third experimental study will finally test, whether behavioural (policy) interventions have a positive or negative impact on these societal effects.

### Limitations

The heuristics and implications of behavioural economics have been integrated into policy analysis and implementations over the past decades, in health care as well as in other policy areas [32, 33, 115–120]. Yet, external validity of results from laboratory experiments, especially from social-preference games, depend greatly on the relevant context of the experiment [33, 64, 119, 121–126]. A concern towards the study presented could be the transferability of observed behaviour from the mean participant to a professional negotiator in a real life reimbursement situation [33]. While the difference in experience or sophistication should not be neglected, we still deem the results relevant due to two main reasons. On one side empirical research comparing inexperienced with experienced and professional traders in financial markets have shown, “that experience reduces behavioural biases, but biases remain relevant even for experienced traders” [33, 127–135]. This has recently been confirmed for bounded rationality and social preferences in experiments between physician and student populations [69, 71, 72]. On the other side certain biases are much more likely to be linked to “trade uncertainties” and related anticipated regrets which cannot be fully reduced by market experience [44–48]. At least not in markets where “the subjects of the negotiation as well as the relevant rules of the game” are relatively complex and less stable compared to simple, repetitive good exchanges [33]. On a continuum of trade complexity pharmaceutical reimbursement negotiations might be closer to financial market trades than to repetitive trades of exchangeable commodities. However, the weakness of the presented study is certainly the fact that it does not reflect the expressed preferences of professional individuals, which have a direct influence on the reimbursement of new specialty pharmaceuticals in real-world. Moreover, it focuses on a hypothetical situation that had social consequences (on patients and fellow players), but did not take into account long-term consequences due to dependence on an organisation as an employed or mandated negotiator. As mentioned at the beginning, we see our study as bridging the gap between established behavioural research in health economics on the one hand and the current discourse in research and politics on reforming pricing policies for new specialty pharmaceuticals on the other. Further research along these lines could seek to connect with the policy-oriented research with observational data of expert surveys on reimbursement decisions (see [99], as well as [8, 26, 100]). Laboratory experiments can serve as complements to non-experimental methods [119] and have the potential to be a “‘wind tunnel’ before implementing large-scale studies or institutional changes of the healthcare market” [120, 136]. In our setting of interest their potential in this regard might even be higher, since reliable real-world data on pharmaceutical pricing negotiations is hardly available, not least because of confidential agreements in many European countries [5, 8, 25, 26, 33, 100].

## Conclusions

Price negotiations for specialty pharmaceuticals take place in a complex market setting. The determination of the added value of a novel treatment and the related societal willingness to pay are of increasing importance in policy reform debates [5, 16, 21–25]. Not only the pricing rules but also the process of reimbursement negotiation itself is subject to demands for reform [5, 7, 26–28]. From a behavioural economics perspective potential cognitive biases affecting negotiations outcomes are of interest [28, 31, 33, 34]. Laboratory experiments could provide a useful test environment for behavioural policy interventions. Assuming that bounded rationality and other-regarding concerns may differ between inexperienced and experienced traders, but remain relevant in both. As is the case in other markets with complex transactions. Our findings show, that the price magnitude in a reimbursement negotiation for pharmaceuticals in oncology has an impact on stated preferences for incremental survival. Further, the assigned role in the negotiation has an impact on the stated relevance of affected stakeholders. Both could have an undesirable influence on reimbursement negotiations. The design was found useful to further assess the effects of the negotiation setting on societal outcomes like cost and availability of new specialty pharmaceuticals in an experimental setting and test appropriate behavioural policy interventions.

## Supplementary Information

**Additional file 1.** Selected screens and payoff details.

**Additional file 2.** Details on model and hypotheses.

**Additional file 3.** Complete instructions for the first run of the experiment.

**Additional file 4.** Complete instructions for the second run of the experiment.

## Data Availability

Experimental instructions used in this and the subsequent study [33] are available in Additional file 3 and Additional file 4. Datasets (fully anonymized) analysed during the current and the subsequent study [33] are available in the zenodo repository 10.5281/zenodo.3575971 upon request.

## Abbreviations

100 k$: 100,000 $
CI: Confidence intervals
ICER: Incremental cost-effectiveness ratio
MTurk: Amazon Mechanical Turk
OECD: Organisation for Economic Co-operation and Development
QALM: Quality adjusted life month
QALY: Quality-adjusted life year
QoL: Quality of life
REG: Regulator
SEL: Seller
SoC: Standard of care
USD: US Dollar
WTA: Willingness to accept
WTP: Willingness to pay

## Footnotes

1 We use the term “pharmaceutical” in this study synonymous to the terms “medicine”, “medicinal product” or “drug product”. Following the definition of the World Health Organization (WHO) and the European Medicines Agency (EMA) this refers to any “substance or combination of substances that is intended to treat, prevent or diagnose a disease, or to restore, correct or modify physiological functions by exerting a pharmacological, immunological or metabolic action” [1–3]. This definition is similar to the one used in the United States by the Food and Drug Administration (FDA) [4]. Our study focuses on the reimbursement of “new high-cost innovative medicines” (European Commission [5]) which are often (predominantly) categorized as “specialty pharmaceuticals” [6]. There is no standard definition of a speciality pharmaceutical, let alone a scientific definition of an “innovative” therapy [6, 7]. Following the frequently referenced definition of IQVIA, we understand it to mean pharmaceutical products that treat chronic, complex or rare diseases (i.e. often more serious medical conditions such as cancers, autoimmune diseases, hepatitis C, etc.) [6, 8]. They are initiated or managed by a specialist and are typically offered at a high list price, so patients need financial support to pay for them [6].

2 The earlier explanations suggested that individuals experience disutility from losing an endowed good (loss aversion) which triggers reluctance to trade [34, 40]. Later attempts explain the phenomenon e.g. by extra utility experienced from owning a good, respectively a different valuation depending on ownership or role [34, 42, 43]. Newer evidence suggested to explain valuation gaps by choice or trade uncertainty or by regret-avoidance [44–48]. None of these attempts has been able to predict all published empirical findings [34, 38].

3 The present study on social preferences forms the basis for a follow-up study in which incentivized bargaining behaviour was investigated [33]. In the following, the basic design for both studies is described, the outlines of which were published in summary form in the subsequent study [33].

4 Donated to the Leukemia & Lymphoma Society (LLS). Participants were further informed, that LLS “provides financial support for patients with blood cancer (https://www.lls.org/support/financial-support)”.

5 Evidence from experimental research has shown that meaningful context can aid in understanding and reduce confusion and implicit associations which helps to gain experimental control [77]. In order to reduce heterogeneous perceptions and expectations of the participants regarding their own role in a pharmaceutical reimbursement negotiation, the simplified context of a fictitious country was introduced.

6 Constant elasticity of substitution

7 For considerations on efficiency concerns see for example [83].

8 The incremental cost-effectiveness ratio of the given standard of care versus no treatment can be derived by dividing the incremental costs (50,000$-0$) by the incremental, quality adjusted survival (5×0.5–0×0.5). For details on efficiency and effectiveness measures, see Additional file 2. For considerations on simplifying heuristics, or on efficiency concerns from a behavioural economics perspective, see for example [67, 73, 83, 86].

9 For considerations on valuations gaps see e.g. [34, 38–41].

10 The experiment was implemented as Qualtrics survey and linked on MTurk as Human Intelligence Task (HIT). The Decision Science Laboratory of ETH Zurich (DeSciL) executed the experimental runs and delivered anonymized data to the researchers. Bonus distributions were performed by the DeSciL to ensure participants remain anonymous to the researchers. The first run of the experiment was conducted on 18 February 2019, run two on 2 May 2019. We ran a technical pre-test of the survey on 22 December 2018 (*n* = 31) to check for basic understanding by MTurk users, the indicated estimation of time needed in the informed consent and potential errors in the survey flow.

11 The price range started at 50,000$ (representing the price of the available standard of care SoC) and ended at 500,000$ for the 100 k$ group, respectively at 0.5$ and 5$ for the 1$ group. The slider allowed the participant to “discretely” compare choices in the price range by increments of 2000$ (100 k$ group) respectively 0.02 (1$ group). Default position of the slider was SoC at the very left.

12 Meaning that participants did not know which of the games they played would be relevant for payoff. This has been used in similar laboratory experiments, based on the findings of Brandts and Charness (2011) who found no difference between the strategy method, where players make “conditional decisions for each possible information set” versus the direct-response method [67, 80, 85, 89, 90].

13 To obtain actual WTP and WTA values for the negotiation setting, a single binary or dichotomous choice would not have been sufficient. This was however crucial for the extended setting of the follow-up study with price offers paired with an actual negotiation partner [33]. Alternatively, the decisions would have had to be repeated in an iterative process, which would have meant an inflationary increase in the duration per round and the experiment overall. In addition, the statements in these procedures can also be distorted by anchor effects and interpretation errors due to the increased complexity of the decision [91]. Due to the complexity of the decision situation the implementation of a single bid mechanism like the Becker-DeGroot-Marschak (BDM) [93] or the Random Price Voting Mechanism (RPVM) [94] had to be abandoned as well, in light of the known challenges with the BDM mechanism [34, 95, 96].

14 A closed-ended format was chosen since an unlimited price range would have required players to be provided with unlimited starting capital to avoid a negative payoff for payers. According to standard laboratory policies, participation in an experiment may not result in any payment obligation for the players in real world. Otherwise, a price increase by the players above a certain level would have no or a distorted distribution effect.

15 Consequently, in the broadest sense, participants had to choose from a (multinomial) discrete ‘choice set’, where the set was however not observable in a single view with all properties per choice.

16 It would also so interesting to assess whether the patient-orientation in professional negotiators on the selling side is higher considering the current attempts of the pharmaceutical industry to become more patient-centric [110, 111].
