# Supplementary material for: Assessing social preferences in reimbursement negotiations for new Pharmaceuticals in Oncology: an experimental design to analyse willingness to pay and willingness to accept
Source: BMC Health Serv Res. 2021 Mar 16;21:234. doi: 10.1186/s12913-021-06231-8 (PMC7968195; doi:10.1186/s12913-021-06231-8)
Supplement: Supplementary file 1 — Additional file 1. Selected screens and payoff details. [file 12913_2021_6231_MOESM1_ESM.pdf]

# Additional file 1: Selected screens and payoff details

## Figure 4 and 5: reservation price decision screen both roles (100k$ groups 1-4)

**Training round 1 (of 4):**

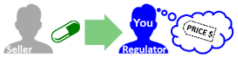


The Pharma Company offers a new pharmaceutical treatment which prolongs the survival of the patient by **six** months (compared to no treatment), increasing the life expectancy by **one** additional month compared to the current standard therapy. The treatment does not increase the quality of life compared to the standard treatment.

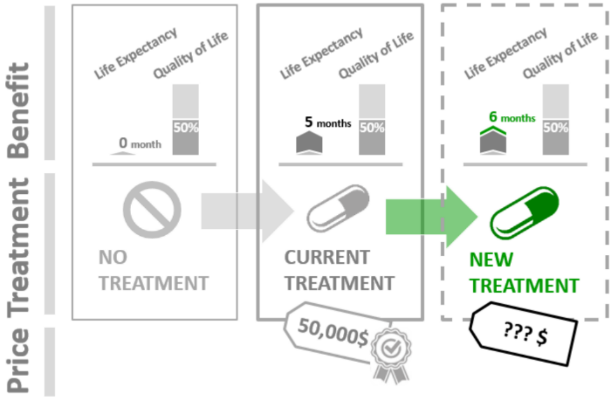


You expect the offer from the pharma company shortly. Before you enter into a negotiation, you should decide on the **absolute maximum price, which you would still consider reasonable and fair for the new product:**

| Your maximum price of ___,000 $ leads to the following state of the society: | | | |
|---|---|---|---|
| *in thousand Dollars* | **New Benefit** | **Compared to Current State** | **New Asset (Benefit + Initial Asset)** |
| **Patient** | 30 | +5 | 30 |
| **2 Payers** | | | |
| **2 Investors** | | | |
| **Seller** | 120 | 0 | 240 |
| **Regulator** | 120 | 0 | 240 |

Please select <u>your maximum price</u> by moving the red slider below.
You will see the related consequences in the table above.
*If the values do not change, please klick the red slider again.*

**Training round 1 (of 4):**

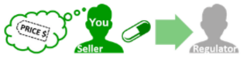


Your company developed a new treatment which prolongs the survival of the patient by **six** months (compared to no treatment), increasing the life expectancy by **one** additional month compared to the current standard treatment. The treatment does not increase the quality of life compared to the standard treatment.

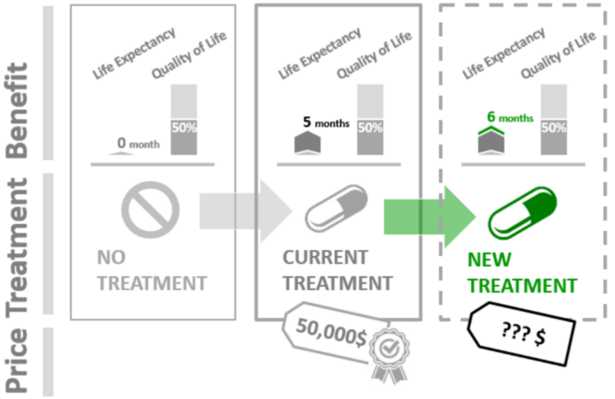


You have to prepare an offer for the Health minister shortly. Before you enter into a negotiation, you should decide on the **absolute minimum price, which you would still consider reasonable and fair for the new product:**

| Your minimum price of ___,000 $ leads to the following state of the society: | | | |
|---|---|---|---|
| *in thousand Dollars* | **New Benefit** | **Compared to Current State** | **New Asset (Benefit + Initial Asset)** |
| **Patient** | 30 | +5 | 30 |
| **2 Payers** | | | |
| **2 Investors** | | | |
| **Seller** | 120 | 0 | 240 |
| **Regulator** | 120 | 0 | 240 |

Please select <u>your minimum price</u> by moving the red slider below.
You will see the related consequences in the table above.
*If the values do not change, please klick the red slider again.*

Figures extracted from the experimental survey used in this and the subsequent study [1]. The experimental surveys are available in Additional file 3 and 4. The illustrations are the authors' own creations.

# Figure 6: run 02, control questions Q1 and Q2

The **Seller** offers the new treatment at a proposed price, which has to be approved by the government (**Regulator**). If the **Regulator** considers the price as to high, he/she will refuse to approve the product. Vice versa, if the **Seller** is confronted with a counterproposal (reduced price) considered too low, he/she will not introduce the product in this market.

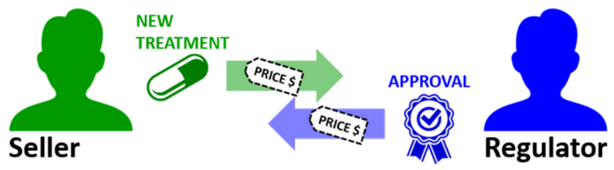


If both parties agree, the **Patient** will get access to the new treatment with all its benefits (life expectancy, quality of life). The **Investors** will in consequence receive the price (revenue) and the **Payers** will have to pay the price (cost).

As long as the regulator and the seller do not agree, the product will not be available, which means no additional benefit for the patient, no additional costs for the premium payers and no revenue for investors. The regulator and the seller are both employed and receive a fix salary.

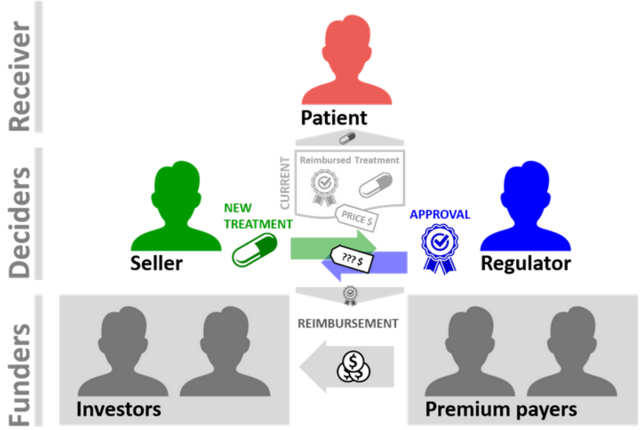


**Control question 1**: What happens, if the **seller** proposes the new product at a price much higher than the regulator wants to accept? Please select all correct answers below:

- The company will still sell the new product, since the patient pays for his treatment out-of-pocket in this country.
- The patient will not get access to the new treatment, since the new product will not be reimbursable by the public health insurance.
- The seller and the regulator will not receive their fix salary.
- The premium payers will have to pay for the new treatment at an unreasonable high price.
- The investors earn more money on their risky investment than they originally expected.

**Control question 2**: Would your answer be different if the **regulator** askes for a price of the new product lower than the lowest price the seller wants to accept?

- Yes, this is different.
- No, the effect is the same as in the question above.

Figure extracted from the experimental survey used in this and the subsequent study [1]. The experimental surveys are available in Additional file 3 and 4. The illustrations are the authors' own creations.

Figure 7 and 8: run 02, role introduction (left group 4: seller 100k$, right group 5: regulator 1$)

You represent an international pharmaceutical company as **Seller**. Your company focuses on research and development (R&D) of new pharmaceuticals. In the following, you will be responsible to **sell new products to the Health Minister** of this country. You will see their expected benefit, based on clinical studies.

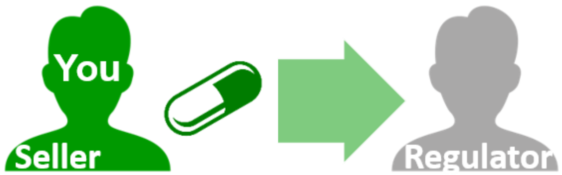


Before you enter into a negotiation, you should decide on the **absolute minimum price, which you would still consider reasonable and fair for your new product**. Bellow this "walk-away price" you would never agree to sell the new pharmaceutical in this country.

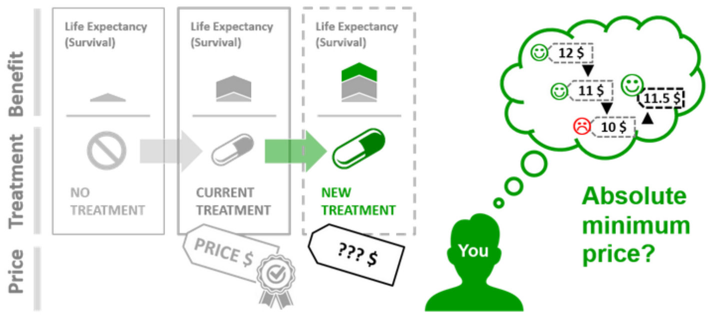


This part of the experiment focuses only on your private valuation of new health technologies. You do not have to reflect on any negotiation tactics or strategies. There is no "correct" answer; your decision should be based solely on your personal preferences.
Be aware that you will not be able to change your decisions after submission.

All prices are expressed in fictive "Dollar" ($) and trade at the end of the experiment at a currency rate of **100,000 $ = 1 US$.**

You are the responsible **Regulator** ("Health Minister") in this country. In the following, **you will receive offers from a pharma company** for the reimbursement of new pharmaceuticals. You will see their expected benefit, based on clinical studies.

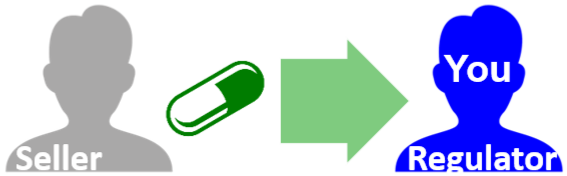


Before you enter into a negotiation, you should decide on the **absolute maximum price, which you would still consider reasonable and fair for the new product**. Above this "walk-away price" you would never allow the new pharmaceutical to be reimbursed by the public health insurance.

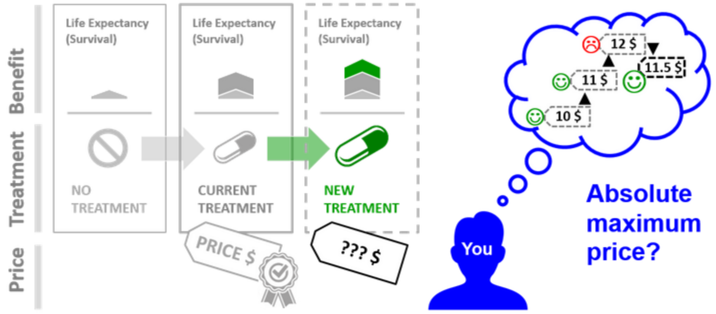


This part of the experiment focuses only on your private valuation of new health technologies. You do not have to reflect on any negotiation tactics or strategies. There is no "correct" answer; your decision should be based solely on your personal preferences.
Be aware that you will not be able to change your decisions after submission.

All prices are expressed in fictive "Dollar" ($) and trade at the end of the experiment at a currency rate of **1 $ = 1 US$**.

Figures extracted from the experimental survey used in this and the subsequent study [1]. The experimental surveys are available in Additional file 3 and 4. The illustrations are the authors' own creations.

Followed by comprehension question Q3 (Figure 9 and 10 below) displayed on the same screen.

Figure 9 and 10: run 02, comprehension question Q3 (left group 4: seller 100k$, right group 5: regulator 1$)

**Control question 3**: Let us pretend that you are a coffee shop owner. We further assume that your customers are real coffee lovers. Meaning that they always benefit more from two cups than from one, more from a large cup than from a small one (see picture below). Let us finally assume that 1 $ represents the minimum price you still consider reasonable and fair for a **small** cup of 12 oz.

a) Assuming that increasing your coffee output requires additional effort (investment) from you: Which price would represent your absolute minimum willingness to sell a **medium** cup of 16 oz.?

- A price higher than 1 $
- A price of 1 $
- A price lower than 1 $

b) Would you sell the large cup of 20 oz. for 1 $?

- Yes
- No

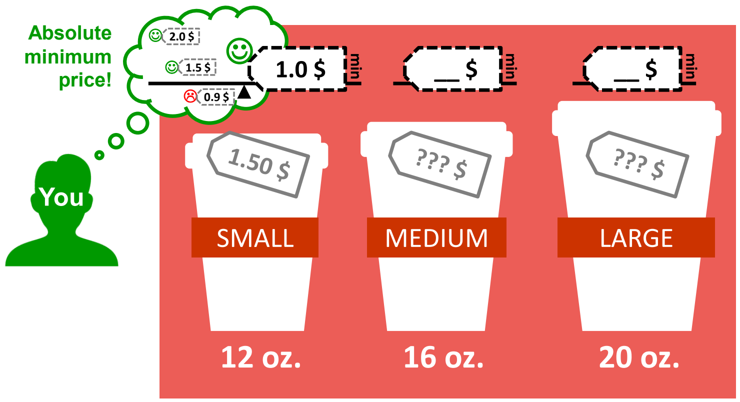


**Control question 3**: Let us assume that you are a real coffee lover (if not, you can replace coffee with soda or else). Meaning that you always benefit more from two cups than from one, more from a large cup than from a small one (see picture below). Let us further assume that 2 $ represents the maximum price you still consider reasonable and fair for a **small** cup of 12 oz.

a) Assuming all other things equal and that you have the money available and can afford it: Which price would represent your absolute maximum willingness to pay for a **medium** cup of 16 oz.?

- A price higher than 2 $
- A price of 2 $
- A price lower than 2 $

b) Would you buy a large cup of 20 oz. for 2 $?

- Yes
- No

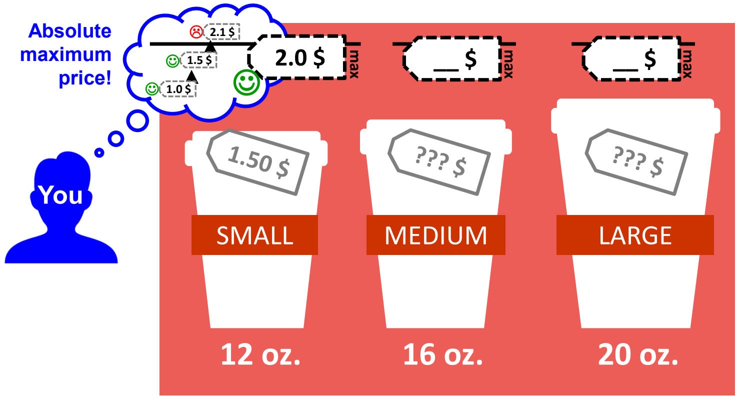


Figures based on the images in the experimental survey used in this and the subsequent study [1]. The experimental surveys are available in Additional file 3 and 4. The illustrations are the authors' own creations. In the original images, the coffee cups are adjusted from wikiHow: How to Order Coffee. wikiHow Inc.; 2019.

Figure 11: run 02, instructional manipulation check ("screener") to identify inattention

**Decision situation (1 of 1):**

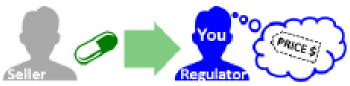


Now the Pharma Company offers a new treatment which prolongs the survival of the patient by minus **one month** (compared to no treatment), decreasing the life expectancy below **zero**. This obviously makes no sense. We just need to test, whether participants read the relevant information. If you did so, please click on the green pill below and submit without entering a price.

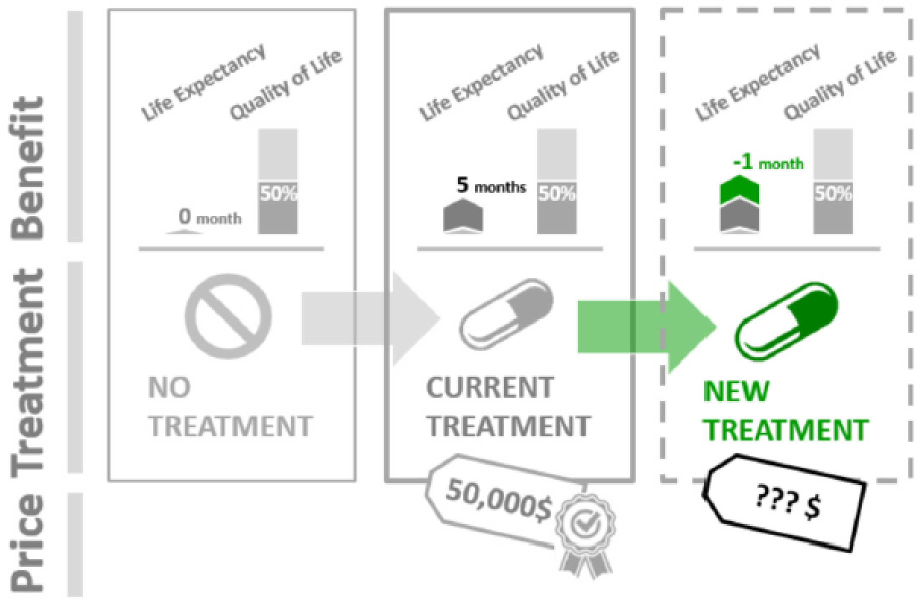


You expect the offer from the pharma company shortly. Before you enter into a negotiation, you should decide on the **absolute maximum price, which you would still consider reasonable and fair for the new product:**

| Your maximum price of ___,000 $ leads to the following state of the society: | | | |
|---|---|---|---|
| *in thousand Dollars* | **New Benefit** | **Compared to Current State** | **New Asset (Benefit + Initial Asset)** |
| **Patient** | -5 | -30 | -30 |
| **2 Payers** | | | |
| **2 Investors** | | | |
| **Seller** | 120 | 0 | 240 |
| **Regulator** | 120 | 0 | 240 |

Figure extracted from the experimental survey used in this and the subsequent study [1]. The experimental surveys are available in Additional file 3 and 4. The illustrations are the authors' own creations.

Figure 12: decision table with message for price entries equal or lower than previous round (run two only)

| Your maximum price of 160000 $ leads to the following state of the society: | | | |
|---|---|---|---|
| *in thousand Dollars* | **New Benefit** | **Compared to Current State** | **New Asset (Benefit + Initial Asset)** |
| **Patient** | 50 | +25 | 50 |
| **2 Payers** | 80 | -110 | 320 |
| **2 Investors** | 160 | +110 | 400 |
| **Seller** | 120 | 0 | 240 |
| **Regulator** | 120 | 0 | 240 |

You did not increase your price although the patient outcome of the product increased. Does this truly reflect your preference?

Please select your minimum price by moving the red slider below.
You will see the related consequences in the table above.
*If the values do not change, please click the red slider again.*

*Your previous decision:*
*8 months: 162,000 $*

Figure extracted from the experimental survey used in this and the subsequent study [1]. The experimental surveys are available in Additional file 3 and 4. The illustrations are the authors' own creations.

Figure 13: initial state (introduction screen, currency group 100k$) and comprehension question Q4 (all displayed on same screen)

**Initial position:**

Patients suffer from a deadly, incurable blood cancer. With no treatment, they have a remaining life expectancy below one month. There is one pharmaceutical treatment available, which increases the patient's life expectancy (*survival*) by **five months** at an unchanged quality of life (*QoL*). The QoL is an experience-based, self-reported indicator for the patient's physical functioning, bodily pain, as well as mental, emotional and social functioning etc. It is measured at a scale from 0 to 100%. The lower the score the more disabled the patient. The QoL of the patient under current standard treatment is **50%**.

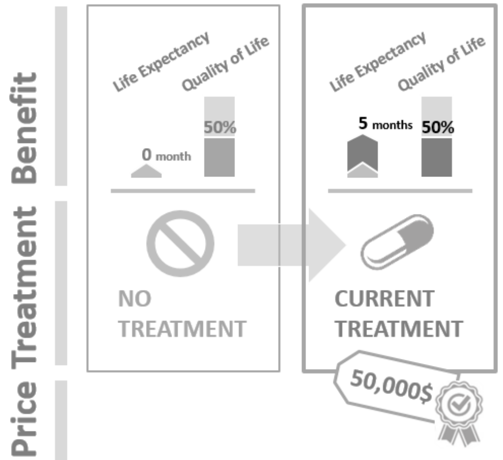


At full QoL (100%) the patient could realize a monthly income of 10,000 $. Due to the lower QoL the patient's work ability (productivity) is reduced proportionally. In consequence the potential income he/she can generate equals 10,000 $ * 50% = 5,000 $ per month. This translates into a total economic benefit for the patient under current standard therapy of 10,000 $ * **50%** * **5** months = 25,000 $.

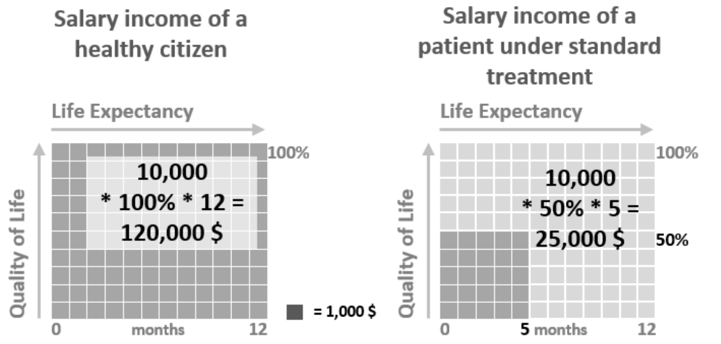


With exception of the patient, all citizens have an initial asset of 120,000 $ (equals to a yearly income at full work ability). In addition to this, the Health minister (you) as well as the representative from the pharma company expect both a fix salary of 120,000 $. The two payers will receive the same income of 120,000 $ but will have to share the health care costs, deducted from their yearly income. The two investors have no fix income but will share the revenue generated with the reimbursed pharmaceuticals.

The current standard treatment costs **50,000 $** per therapy and patient, paid by the health insurance.
In consequence each of the two payers earns currently 95,000 $ (= 120,000 – 50,000 / 2) and each of the two investors 25,000 $ (= 50,000 / 2).

---

**Control question 4**: If the patient's survival increases from zero to 4 months at the same quality of life (50%), how much will he/she gain "economically" in cash?

- Nothing, the benefit of survival cannot be expressed in monetary terms.
- Nothing, the patient cannot work at a reduced quality of life.
- 20,000 $
- 25,000 $
- 40,000 $

Figure extracted from the experimental survey used in this and the subsequent study [1]. The experimental surveys are available in Additional file 3 and 4. The illustrations are the authors' own creations.

***Payoffs to stakeholders:***

The experiment was implemented as Qualtrics survey and linked on MTurk as Human Intelligence Task (HIT). The Decision Science Laboratory of ETH Zurich (DeSciL) executed the experimental runs and delivered anonymized data to the researchers. Bonus distributions were performed by the DeSciL to ensure participants remain anonymous to the researchers.

- All prices expressed in fictive “Dollar” ($) during the game traded at the end of the experiment at a currency rate of 100,000 $ = 1 US$ for group 1 to 4. Prices for group 5 to 6 traded at par.
- Patient: benefit converted to US$ (divided by ten for run one) was donated to the Leukemia & Lymphoma Society (LLS) which provides financial support for patients with blood cancer (https://www.lls.org/support/financial-support).
- Payers and investors: benefit plus initial assets converted to US$ divided by ten was paid to four other MTurk-Users (randomly selected, only positive amounts implemented).
- Regulator or Seller: A fix amount of 2.4 US$ was paid to each participant (voucher right at the end of the survey) corresponding to a “yearly income” plus initial assets in the experiment. Bonus was distributed by the DeSciL, after the run was closed.

  Bonus run one: participants received an additional bonus of 0.6 US$ for participating in the voluntary introductory training (corresponding to 25% of fix salary).

  Bonus run two (covered in subsequent study [1]): no bonus for the mandatory training. Participants received instead the difference between their price offer and their reservation price, if their offer lead to an agreement for any successful round in game two. This was determined by a random pairing of regulators and sellers at the end of the experiment.

References

1. Wettstein DJ, Boes S. The impact of reimbursement negotiations on cost and availability of new pharmaceuticals: evidence from an online experiment. *Health Econ Rev*. 2020;10(1):13.
