# Supplementary material for: Assessing social preferences in reimbursement negotiations for new Pharmaceuticals in Oncology: an experimental design to analyse willingness to pay and willingness to accept
Source: BMC Health Serv Res. 2021 Mar 16;21:234. doi: 10.1186/s12913-021-06231-8 (PMC7968195; doi:10.1186/s12913-021-06231-8)
Supplement: Supplementary file 2 — Additional file 2. Details on model and hypotheses. [file 12913_2021_6231_MOESM2_ESM.pdf]

Additional file 2: Model and hypotheses[1]

Model and hypotheses

A "robust finding" from past laboratory experiments in economics is that "individuals take into account the welfare of all parties and have a preference for efficient outcomes" [2-6,1] and that "non-selfish preferences are the rule rather than the exception" [7]. Building on these research results, the model used in this study was based on a simple CES[2]-function, where a rational decider (regulator R or seller S) should maximize his/her social utility (U) considering the utility (benefit π) of the other involved stakeholders besides his own: U (π_R,π_S,π_C,π_P,π_I), including the patient's (consumer C), the payers' (P) and the investors' (I) benefit.

a) Stated preferences: reservation prices

Since payoffs in the reservation price games are for both deciding roles identical, independent of their decision (x) and flat during the game (fix salary) the utility functions can be simplified for both deciding roles (D): U_D (π_C,π_P,π_I) = $(\gamma_D \pi_C^\rho + \delta_D \pi_P^\rho + \varepsilon_D \pi_I^\rho)^{1/\rho}$ with γ=1-δ-ε for the weighting of the affected stakeholders' benefits.

- Hypothesis H0-I: the two deciding roles weight the benefit of the other stakeholders with zero (no social or distributional preferences with γ=δ=ε=0), hence reservation prices (x) are randomly distributed between 50,000 $ and 500,000 $ in each round and do not differ between rounds with incremental patient benefit ($E[X_r]$ = 275,000 $ for r = {1;…;5}).
- Hypothesis H0-II: the deciders are inequality averse regarding funders only. Since π_D is independent of (x) and since π_P and π_I are assumed to be symmetrical, both deciding roles

[1] The present study on social preferences forms the basis for a subsequent study in which incentivized bargaining behaviour was investigated [1]. The model described was also used in an adapted form in the subsequent study [1].
[2] Constant elasticity of substitution

will simply redistribute the total assets (all initial assets 4*120'000 plus initial premium benefit of the payers 2*120,000 $) to minimize the difference between the funders (payer, investor) by choosing x=120,000 $, indifferent between any patient outcome (patient benefit, affected by incremental survival and QoL), assuming that:
- they do not care for pareto-efficiency (since "payer" framed as being "available to pay"),
- they have an identical utility distribution as per defined model,
- they have equal agency/sympathy for the funders with $\delta=\varepsilon$.

- Hypothesis H0-III: reservation prices in relation to the patient outcome gained do not differ comparing each round to the standard of care, using ICER (see Table 3 and 4 further below). Since QoL and monthly income are constant in this experiment, the respective price to keep each incremental constant between rounds does not differ between alternative measures.
  - Hypothesis H0-IIIb: reservation prices in relation to the patient outcome gained do not differ between consecutive rounds, using ICER.
- Hypothesis H0-IV: difference due to price magnitude framing (hypothetical 100k$ vs 1$ price group) has no effect since effective payoffs at the end of the experiment converted to USD are identical for all groups.

- Hypothesis H0-V: since payoff-functions are identical for both deciding roles and indifference curves are reversible, for every round WTP equals WTA, hence the assigned role has no effect on the price decision (no valuation differences) with $\gamma_R=\gamma_S$, $\delta_R=\delta_S$ and $\varepsilon_R=\varepsilon_S$.

b) Stated preferences: relevance of stakeholders

- Hypothesis H0-VI: since payoff-functions are identical for all groups and indifference curves are reversible, ranking of the stakeholders' relevance is identical.

Efficiency and effectiveness

Efficiency and effectiveness measures:

i. WTP or WTA per life month gained: reservation price in proportion to the incremental survival, compared to no treatment
(x_r / m_r)

ii. WTP or WTA per quality adjusted life month (QALM) gained: reservation price in proportion to the incremental survival reflecting quality of life
$(x_r / m_r * q_r)$

iii. Incremental cost-effectiveness ratio ICER: difference between the reservation price and the price of the standard treatment in proportion to the QALM gained
$(x_r – x_0) / (m_r * q_r – m_0 * q_0)$

iv. Incremental cost-benefit ratio ICBR: difference between the reservation price and the price of the standard treatment in proportion to the patient benefit (QALM * monthly income) gained
$(x_r – x_0) / (\pi_C(m_r * q_r) – \pi_C(m_0 * q_0))$

v. Added value: difference between the value of the new treatment and the value of the standard treatment. Value is measured by the patient benefit in proportion to the cost of the treatment [8]
$\pi C(mr * qr) / xr – \pi C(m0 * q0) / x0$

Table 4: efficiency and effectiveness measures

i) WTP or WTA per life month gained: $\frac{x_r}{m_r}$	ii) WTP or WTA per quality adjusted life month (QALM) gained $\frac{x_r}{m_r \times q_r}$
iii) Incremental cost-effectiveness ratio ICER: $\frac{x_r - x_0}{m_r \times q_r - m_0 \times q_0}$	iv) Incremental cost-benefit ratio ICBR: $\frac{x_r - x_0}{\pi_C(m_r, q_r) - \pi_C(m_0, q_0)}$
v) Added value: $\frac{\pi_C(m_r, q_r)}{x_r} - \frac{\pi_C(m_0, q_0)}{x_0}$	

Table 5: Prices for constant efficiency and effectiveness per measure

							Prices for constant…					**…effectiveness & efficiency**				
**Treat**	**Round**	**Survival (months)**	**Δ**	**QoL**	**Δ**	**Patient benefit**	WTP,WTA per incr. Month	WTP,WTA per incremental QALM	ICER (cost per incremental QALM)	Incremental Cost-Benefit Ratio (reflecting monthly income)	Added value	**WTP,WTA per incr. Month**	**WTP,WTA per incremental QALM**	**ICER (cost per incremental QALM)**	**Incremental Cost-Benefit Ratio (reflecting monthly income)**	**Added value**
None		*0*		*0.5*		*0*	*0.0*	*0.0*	*0.0*	*0.0*	*0.0*					
SoC	*0*	*5*	*+5*	*0.5*	*0*	***0.25***	*0.5*	*0.5*	*0.5*	*0.5*	*0.5*	*0.1*	*0.2*	*0.2*	*2*	*nv*
New	1	**8**	+3	**0.5**	0	**0.40**	0.8	0.8	0.8	0.8	0.8	0.1	0.2	0.2	2	0%
	2	**10**	+2	**0.5**	0	**0.50**	1.0	1	1	1	1	0.1	0.2	0.2	2	0%
	3	**12**	+2	**0.5**	0	**0.60**	1.2	1.2	1.2	1.2	1.2	0.1	0.2	0.2	2	0%
	4	**15**	+3	**0.5**	0	**0.75**	1.5	1.5	1.5	1.5	1.5	0.1	0.2	0.2	2	0%
	5	**17**	+2	**0.5**	0	**0.85**	1.7	1.7	1.7	1.7	1.7	0.1	0.2	0.2	2	0%

SoC, standard of care; QoL, quality of life; nv, no value

References

1. Wettstein, D.J., Boes, S.: The impact of reimbursement negotiations on cost and availability of new pharmaceuticals: evidence from an online experiment. Health Econ Rev 10(1), 13 (2020). https://doi.org/10.1186/s13561-020-00267-y
2. Schumacher, H., Kesternich, I., Kosfeld, M., Winter, J.: One, two, many—Insensitivity to group size in games with concentrated benefits and dispersed costs. The Review of Economic Studies 84(3), 1346-1377 (2017).
3. Andreoni, J., Miller, J.: Giving According to GARP: An Experimental Test of the Consistency of Preferences for Altruism. Econometrica 70(2), 737-753 (2002).
4. Charness, G., Rabin, M.: Understanding Social Preferences with Simple Tests. The Quarterly Journal of Economics 117(3), 817-869 (2002).
5. Engelmann, D., Strobel, M.: Inequality aversion, efficiency, and maximin preferences in simple distribution experiments. Am. Econ. Rev. 94(4), 857-869 (2004).
6. Fisman, R., Kariv, S., Markovits, D.: Individual preferences for giving. Am. Econ. Rev. 97(5), 1858-1876 (2007).
7. Bruhin, A., Fehr, E., Schunk, D.: The many faces of human sociality: Uncovering the distribution and stability of social preferences. Journal of the European Economic Association 17(4), 1025-1069 (2018).
8. Porter, M.E., Teisberg, E.O.: Redefining health care: creating value-based competition on results. Harvard Business Press, (2006)
