# Supplementary material for: Assessing social preferences in reimbursement negotiations for new Pharmaceuticals in Oncology: an experimental design to analyse willingness to pay and willingness to accept
Source: BMC Health Serv Res. 2021 Mar 16;21:234. doi: 10.1186/s12913-021-06231-8 (PMC7968195; doi:10.1186/s12913-021-06231-8)
Supplement: Supplementary file 3 — Additional file 3. Complete instructions for the first run of the experiment. [file 12913_2021_6231_MOESM3_ESM.pdf]

Intro_1

This survey is part of a scientific study interested in understanding preferences for new health technologies. You will be asked to assign monetary values to new medical treatments. Your answers will affect others in real-life via monetary payoffs.

The experiment will take approximately 25 minutes. It will start with a detailed description of the decision situation, followed by ten decision situations (rounds) split in two sets of five each. You will receive US$ 2.40 for thorough completion of the experiment. If you participate in the introductory training (four rounds), you will be awarded with an additional US$ 0.6 (corresponding to a 25% bonus).

Your participation is anonymous to the responsible researchers; they will not be able to draw conclusions to you as an individual. The data provided during the survey will be used for scientific purposes only.

Please indicate whether you want to participate in this study:

- ○ Yes
- ○ No

Are you 18+ years old?

- ○ Yes
- ○ No

Are you resident in the USA?

- ○ Yes
- ○ No

These page timer metrics will not be displayed to the recipient.
First Click: *0 seconds*
Last Click: *0 seconds*
Page Submit: *0 seconds*
Click Count: *0 clicks*

Intro_2

Dear participant

Thank you very much in advance for your willingness to contribute to our research!

This study was designed by the University of Lucerne (Switzerland) and carried out in cooperation with the Decision Science Laboratory of the ETH Zurich (Switzerland).

Please read carefully the instructions on the following pages.

These page timer metrics will not be displayed to the recipient.
First Click: *0 seconds*
Last Click: *0 seconds*
Page Submit: *0 seconds*
Click Count: *0 clicks*

May we first ask some basic information about you.

Please select your year of birth.

[⌄]

Social Preferences in Reimbursement Negotiations, Additional file 3 Wettstein D., Boes S. 2021

Please select your gender.

- ○ Male
- ○ Female
- ○ Other

These page timer metrics will not be displayed to the recipient.
First Click: *0 seconds*
Last Click: *0 seconds*
Page Submit: *0 seconds*
Click Count: *0 clicks*

The objective of this research is to find out the revealed “true” value that you assign to a new health technology in comparison to an existing (“standard”) technology.
You are given in each decision situation complete information on how all relevant stakeholders are affected.

Please assume for the following experiment that you belong to a country with in total seven citizens: one *Patient*, one *Regulator (representing the Government)*, one *Seller (representing a pharmaceutical company)*, two *Premium Payers (who finance the public health insurance)* and two *Investors (who financed a new pharmaceutical)*.

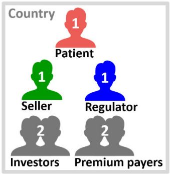

The Patient is suffering from a severe disease: a deadly blood cancer. He/she is under treatment with an existing therapy (current standard of care): a pharmaceutical product with a known benefit to the patient (life expectancy and quality of life).
The Regulator is the responsible “Health Minister” and represents the government. He/she is in charge to regulate prices for pharmaceuticals. Only pharmaceuticals with an official list price are eligible for payment by the public health insurance.
The Seller represents an international pharmaceutical company that developed a new product to treat the patient's disease. He/she is in charge to negotiate with the government an officially reimbursed price.
The Premium Payers finance the public health insurance. Insurance is mandatory and the collected premiums are the only financial source to pay the treatment for any patients in need. However, only treatments approved by the government for reimbursement are covered. If health expenditures are lower than the actual premiums the payers benefit. If the expenditures are higher, they will have to eat up their savings or incur dept.
The Investors have invested their savings in the past into a research-based pharmaceutical company. They expect a return on their investment, which compensates them for the additional risk they took, compared to a ”risk-free” investment in a government bond for example.

These page timer metrics will not be displayed to the recipient.
First Click: *0 seconds*
Last Click: *0 seconds*
Page Submit: *0 seconds*
Click Count: *0 clicks*

The **Seller** offers the new treatment at a proposed price, which has to be approved by the government (**Regulator**). If the **Regulator** considers the price as to high, he/she will refuse to approve the product. Vice versa, if the **Seller** is confronted with a counterproposal (reduced price) considered too low, he/she will not introduce the product in this market.

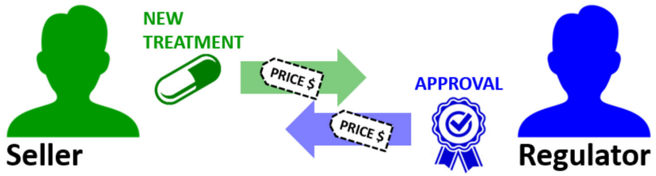

If both parties agree, then the **Patient** will get access to the new treatment with all its benefits (life expectancy, quality of life). The **Investors** will in consequence receive the price (revenue) and the **Payers** will have to pay the price (cost).

 This work is licensed under a Creative Commons Attribution 4.0 International License. Survey created with Qualtrics.

As long as the regulator and the seller do not agree, the product will not be available, which means no additional benefit for the patient, no additional costs for the premium payers and no revenue for investors. The regulator and the seller are both employed and receive a fix salary.

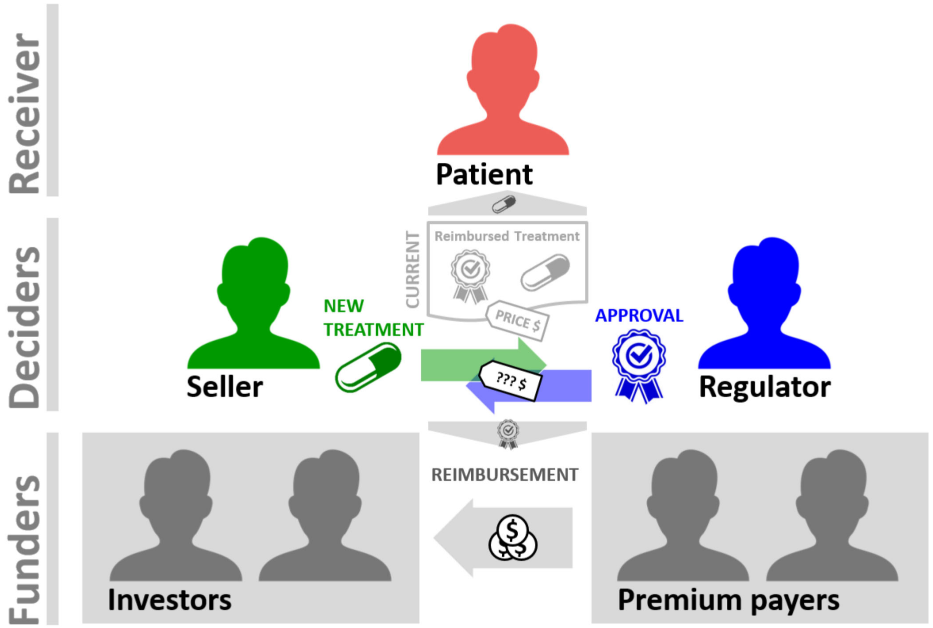

These page timer metrics will not be displayed to the recipient.
First Click: *0 seconds*
Last Click: *0 seconds*
Page Submit: *0 seconds*
Click Count: *0 clicks*

Regulator - Intro & Training

You are the responsible **Regulator** ("Health Minister") in this country. In the following, **you will receive offers from a pharma company** for the reimbursement of new pharmaceuticals. You will see their expected benefit, based on clinical studies.

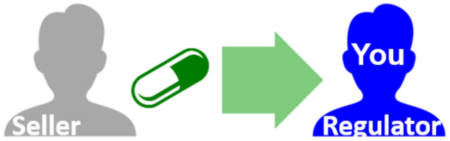

Before you enter into a negotiation, you should decide on the **absolute maximum price, which you would still consider reasonable and fair for the new product**. Above this "walk-away price" you would never allow the new pharmaceutical to be reimbursed by the public health insurance.

This work is licensed under a Creative Commons Attribution 4.0 International License. Survey created with Qualtrics.

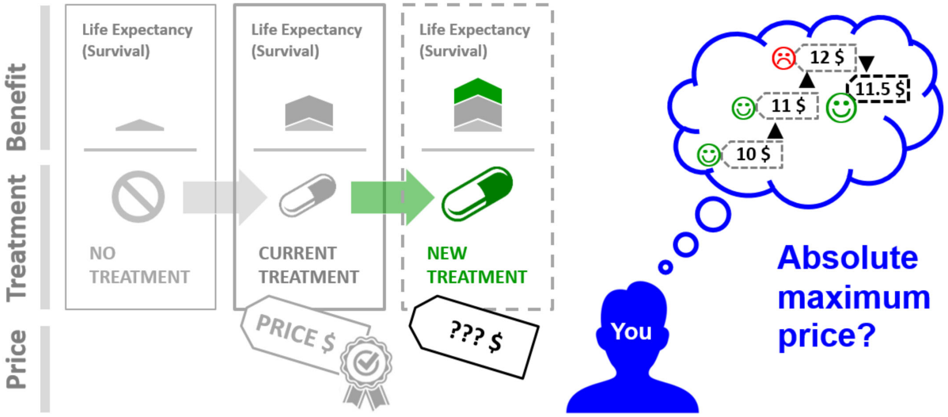

This experiment focuses only on your valuation of new health technologies. The subsequent price negotiation is not part of this survey and you do not have to reflect on any negotiation tactics or strategies. There is no "correct" answer; your decision should be based solely on your own preferences.
Be aware that you will not be able to change your decisions after submission. Assume further, that once an official price is settled with the Seller, it will remain fixed for a longer period.

All prices are expressed in fictive "Dollar" ($) and trade at the end of the experiment at a currency rate of **100,000 $ = 1 US$**.

These page timer metrics will not be displayed to the recipient.
First Click: *0 seconds*
Last Click: *0 seconds*
Page Submit: *0 seconds*
Click Count: *0 clicks*

Your decision will have real consequences on others. After you've finished this experiment, one of your decisions will be selected randomly and implemented as follows:

Patient: Benefit converted to US$ /10 will be donated to the Leukemia & Lymphoma Society (LLS) which provides financial support for patients with blood cancer (https://www.lls.org/support/financial-support)
Payers: Positive benefit converted to US$ /10 will be paid to two other MTurk-Users (randomly selected)
Investors: Positive benefit converted to US$ /10 will be paid to two other MTurk-Users (randomly selected)
Regulator or **Seller**: Benefit converted to US$ will be paid to you (your fictive negotiation partner's decision will be implemented separately based on his or her own responses).

This work is licensed under a Creative Commons Attribution 4.0 International License. Survey created with Qualtrics.

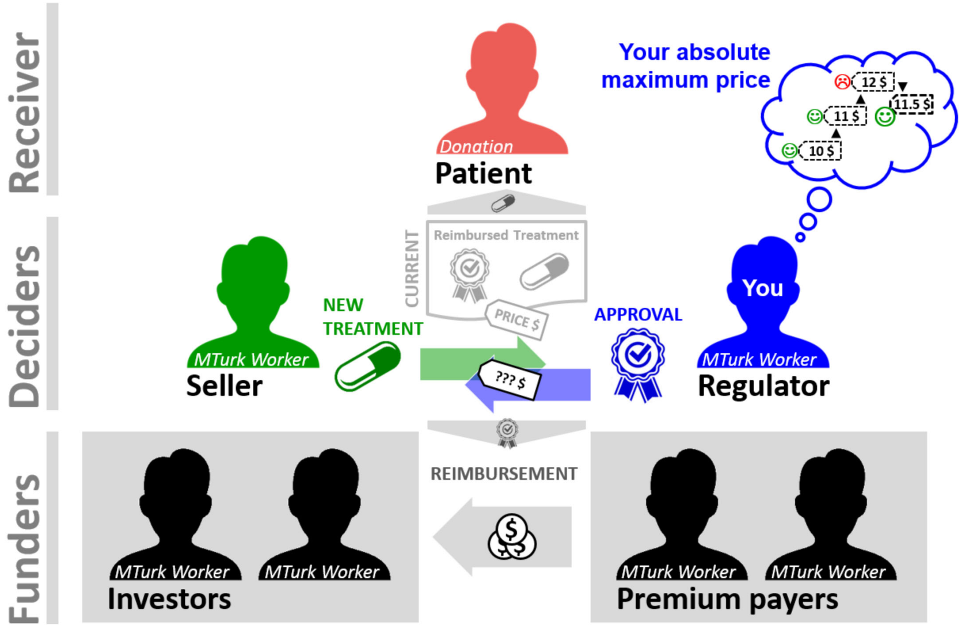

These page timer metrics will not be displayed to the recipient.
First Click: *0 seconds*
Last Click: *0 seconds*
Page Submit: *0 seconds*
Click Count: *0 clicks*

Initial position:

Patients suffer from a deadly, incurable blood cancer. With no treatment, they have a remaining life expectancy below one month. There is one pharmaceutical treatment available, which increases the patient's life expectancy (*survival*) by **five months** at an unchanged quality of life (*QoL*). The QoL is an experience-based, self-reported indicator for the patient's physical functioning, bodily pain, as well as mental, emotional and social functioning etc. It is measured at a scale from 0 to 100%. The lower the score the more disabled the patient. The QoL of the patient under current standard treatment is **50%**.

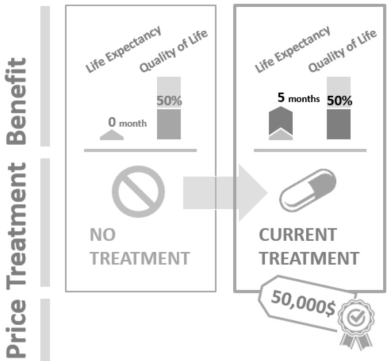

At full QoL (100%) the patient could realize a monthly income of 10,000 $. Due to the lower QoL the patient's work ability (productivity) is reduced proportionally. In consequence the potential income he/she can generate equals 10,000 $ * 50% = 5,000 $ per month. This translates into a total economic benefit for the patient under current standard therapy of 10,000 $ * **50%** * **5** month = 25,000 $.

This work is licensed under a Creative Commons Attribution 4.0 International License. Survey created with Qualtrics.

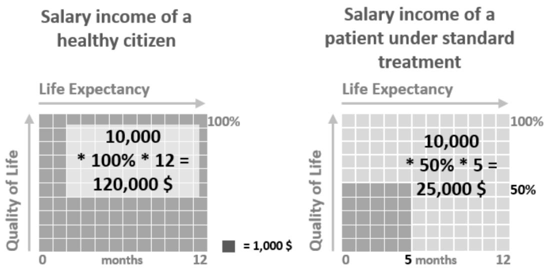

With exception of the patient, all citizens have an initial asset of 120,000 $ (equals to a yearly income at full work ability). In addition to this, the Health minister (you) as well as the representative from the pharma company expect both a fix salary of 120,000 $. The two payers will receive the same income of 120,000 $ but will have to share the health care costs, deducted from their yearly income. The two investors have no fix income but will share the revenue generated with the reimbursed pharmaceuticals.

The current standard treatment costs **50,000 $** per therapy and patient, paid by the health insurance.
In consequence each of the two payers earns currently 95,000 $ (= 120,000 – 50,000 / 2) and each of the two investors 25,000 (= 50,000 / 2).

These page timer metrics will not be displayed to the recipient.
First Click: *0 seconds*
Last Click: *0 seconds*
Page Submit: *0 seconds*
Click Count: *0 clicks*

You have now the option to participate in a short training (4 decisions), before starting the actual experiment. We encourage you to do so. For the thorough completion of the training you will be awarded with additional US$ 0.6 after the experiment.

- ○ YES, please start the training
- ○ NO, I prefer to skip the training

These page timer metrics will not be displayed to the recipient.
First Click: *0 seconds*
Last Click: *0 seconds*
Page Submit: *0 seconds*
Click Count: *0 clicks*

Training round 1 (of 4):

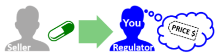

The Pharma Company offers a new pharmaceutical treatment which prolongs the survival of the patient by **six** months (compared to no treatment), increasing the life expectancy by **one** additional month compared to the current standard therapy. The treatment does not increase the quality of life compared to the standard treatment.

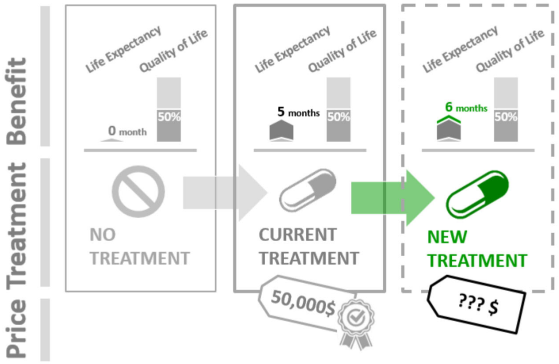

This work is licensed under a Creative Commons Attribution 4.0 International License. Survey created with Qualtrics.

You expect the offer from the pharma company shortly. Before you enter into a negotiation, you should decide on the **absolute maximum price, which you would still consider reasonable and fair for the new product:**

Your maximum price of ___,000 $ leads to the following state of the society:			
in thousand Dollars	**New Benefit**	**Compared to Current State**	**New Asset (Benefit + Initial Asset)**
**Patient**	30	+5	30
**2 Payers**			
**2 Investors**			
**Seller**	120	0	240
**Regulator**	120	0	240

[illegible]

Please select your maximum price by moving the red slider below.
You will see the related consequences in the table above.
If the values do not change, please klick the red slider again.

These page timer metrics will not be displayed to the recipient.
First Click: *0 seconds*
Last Click: *0 seconds*
Page Submit: *0 seconds*
Click Count: *0 clicks*

Training round 2 (of 4):

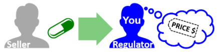

Now the Pharma Company offers a new pharmaceutical treatment which prolongs the survival of the patient by **nine** months (compared to no treatment), increasing the life expectancy by an additional **four** months compared to the current standard therapy. The treatment does not increase the quality of life compared to the standard treatment.

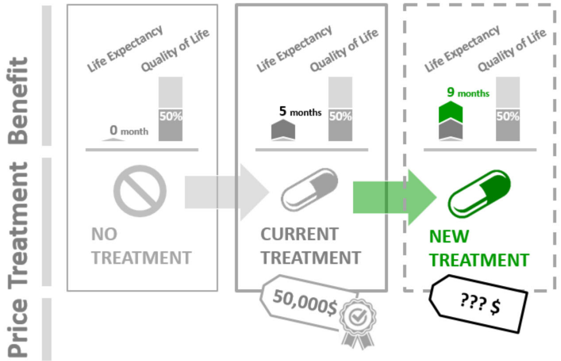

You expect the offer from the pharma company shortly. Before you enter into a negotiation, you should decide on the **absolute maximum price, which you would still consider reasonable and fair for the new product:**

This work is licensed under a Creative Commons Attribution 4.0 International License. Survey created with Qualtrics.

Your maximum price of ___,000 $ leads to the following state of the society:			
in thousand Dollars	**New Benefit**	**Compared to Current State**	**New Asset (Benefit + Initial Asset)**
**Patient**	45	+20	45
**2 Payers**			
**2 Investors**			
**Seller**	120	0	240
**Regulator**	120	0	240

[illegible]

Please select your maximum price by moving the red slider below.
You will see the related consequences in the table above.
If the values do not change, please klick the red slider again.

Your previous decision:
6 months: ${q://QID174/ChoiceNumericEntryValue/1},000

These page timer metrics will not be displayed to the recipient.
First Click: *0 seconds*
Last Click: *0 seconds*
Page Submit: *0 seconds*
Click Count: *0 clicks*

Training round 3 (of 4):

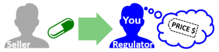

Now the Pharma Company offers a new pharmaceutical treatment which prolongs the survival of the patient by **thirteen** months (compared to no treatment), increasing the life expectancy by an additional **eight** months compared to the current standard treatment. The treatment does not increase the quality of life compared to the standard treatment.

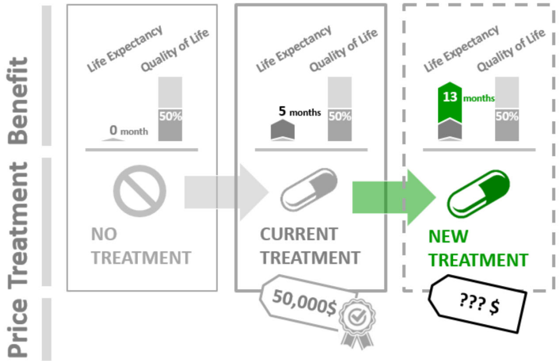

You expect the offer from the pharma company shortly. Before you enter into a negotiation, you should decide on the **absolute maximum price, which you would still consider reasonable and fair for the new product:**

This work is licensed under a Creative Commons Attribution 4.0 International License. Survey created with Qualtrics.

Your maximum price of ___,000 $ leads to the following state of the society:			
in thousand Dollars	**New Benefit**	**Compared to Current State**	**New Asset (Benefit + Initial Asset)**
**Patient**	65	+40	65
**2 Payers**			
**2 Investors**			
**Seller**	120	0	240
**Regulator**	120	0	240

[illegible]

Please select your maximum price by moving the red slider below.
You will see the related consequences in the table above.
If the values do not change, please klick the red slider again.

Your previous decisions:
6 months: ${q://QID174/ChoiceNumericEntryValue/1},000
9 months: ${q://QID178/ChoiceNumericEntryValue/1},000

These page timer metrics will not be displayed to the recipient.
First Click: *0 seconds*
Last Click: *0 seconds*
Page Submit: *0 seconds*
Click Count: *0 clicks*

Training round 4 (of 4):

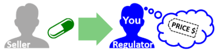

Now the Pharma Company offers a new pharmaceutical treatment which prolongs the survival of the patient by **fourteen** months (compared to no treatment), increasing the life expectancy by an additional **nine** months compared to the current standard treatment. The treatment does not increase the quality of life compared to the standard treatment.

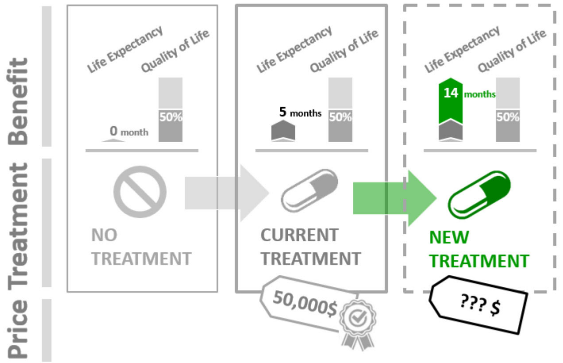

You expect the offer from the pharma company shortly. Before you enter into a negotiation, you should decide on the **absolute maximum price, which you would still consider reasonable and fair for the new product:**

This work is licensed under a Creative Commons Attribution 4.0 International License. Survey created with Qualtrics.

Your maximum price of ___,000 $ leads to the following state of the society:			
in thousand Dollars	**New Benefit**	**Compared to Current State**	**New Asset (Benefit + Initial Asset)**
**Patient**	70	+45	70
**2 Payers**			
**2 Investors**			
**Seller**	120	0	240
**Regulator**	120	0	240

[illegible]

Please select your maximum price by moving the red slider below.
You will see the related consequences in the table above.
If the values do not change, please klick the red slider again.

Your previous decisions:
6 months: ${q://QID174/ChoiceNumericEntryValue/1},000
9 months: ${q://QID178/ChoiceNumericEntryValue/1},000
13 months: ${q://QID182/ChoiceNumericEntryValue/1},000

These page timer metrics will not be displayed to the recipient.
First Click: *0 seconds*
Last Click: *0 seconds*
Page Submit: *0 seconds*
Click Count: *0 clicks*

You have successfully completed the training. Thank you very much!

These page timer metrics will not be displayed to the recipient.
First Click: *0 seconds*
Last Click: *0 seconds*
Page Submit: *0 seconds*
Click Count: *0 clicks*

Seller - Intro & Training

You represent an international pharmaceutical company as **Seller**. Your company focuses on research and development (R&D) of new pharmaceuticals. In the following, you will be responsible to **sell new products to the Health Minister** of this country. You will see their expected benefit, based on clinical studies.

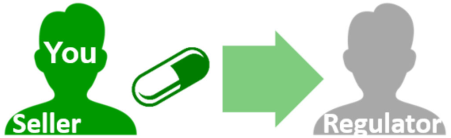

Before you enter into a negotiation, you should decide on the **absolute minimum price, which you would still consider reasonable and fair for your new product**. Bellow this "walk-away price" you would never agree to sell the new pharmaceutical in this country.

This work is licensed under a Creative Commons Attribution 4.0 International License. Survey created with Qualtrics.

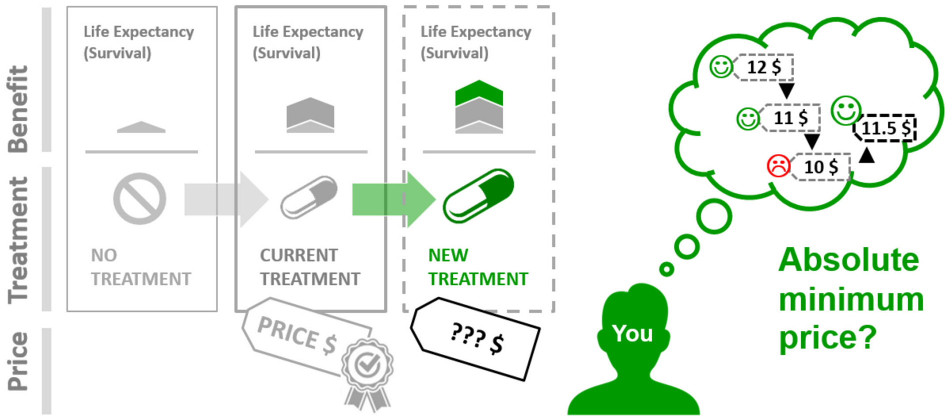

This experiment focuses only on your valuation of new health technologies. The subsequent price negotiation is not part of this survey and you do not have to reflect on any negotiation tactics or strategies. There is no "correct" answer; your decision should be based solely on your own preferences.
Be aware that you will not be able to change your decisions after submission. Assume further, that once an official price is settled with the Government, it will remain fixed for a longer period.

All prices are expressed in fictive "Dollar" ($) and trade at the end of the experiment at a currency rate of **100,000 $ = 1 US$.**

These page timer metrics will not be displayed to the recipient.
First Click: *0 seconds*
Last Click: *0 seconds*
Page Submit: *0 seconds*
Click Count: *0 clicks*

Your decision will have real consequences on others. After you've finished this experiment, one of your decisions will be selected randomly and implemented as follows:

Patient: Benefit converted to US$ /10 will be donated to the Leukemia & Lymphoma Society (LLS) which provides financial support for patients with blood cancer (https://www.lls.org/support/financial-support)
Payers: Positive benefit converted to US$ /10 will be paid to two other MTurk-Users (randomly selected)
Investors: Positive benefit converted to US$ /10 will be paid to two other MTurk-Users (randomly selected)
Regulator or **Seller**: Benefit converted to US$ will be paid to you (your fictive negotiation partner's decision will be implemented separately based on his or her own responses).

 This work is licensed under a Creative Commons Attribution 4.0 International License. Survey created with Qualtrics.

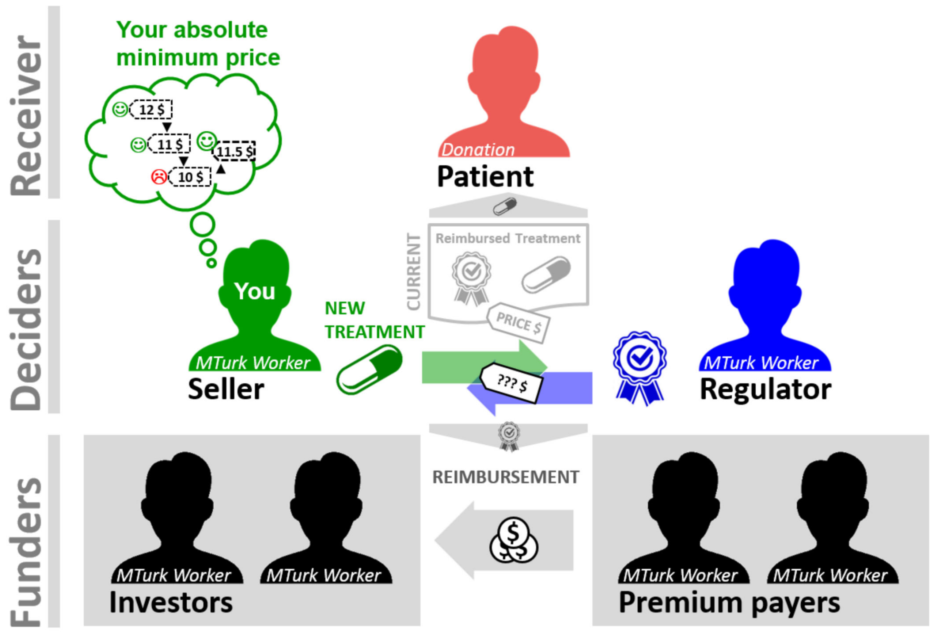

These page timer metrics will not be displayed to the recipient.
First Click: *0 seconds*
Last Click: *0 seconds*
Page Submit: *0 seconds*
Click Count: *0 clicks*

Initial position:

Patients suffer from a deadly, incurable blood cancer. With no treatment, they have a remaining life expectancy below one month. There is one pharmaceutical treatment available, which increases the patient's life expectancy (*survival*) by **five months** at an unchanged quality of life (*QoL*). The QoL is an experience-based, self-reported indicator for the patient's physical functioning, bodily pain, as well as mental, emotional and social functioning etc. It is measured at a scale from 0 to 100%. The lower the score the more disabled the patient. The QoL of the patient under current standard treatment is **50%**.

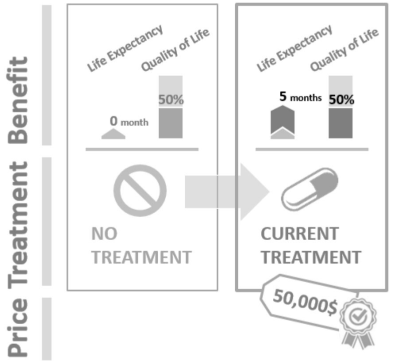

At full QoL (100%) the patient could realize a monthly income of 10,000 $. Due to the lower QoL the patient's work ability (productivity) is reduced proportionally. In consequence the potential income he/she can generate equals 10,000 $ * 50% = 5,000 $ per month. This translates into a total economic benefit for the patient under current standard treatment of 10,000 $ * **50%** * **5** month = 25,000 $.

 This work is licensed under a Creative Commons Attribution 4.0 International License. Survey created with Qualtrics.

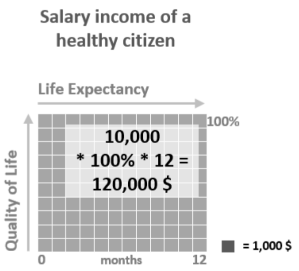

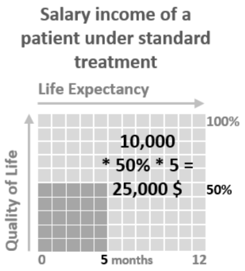

With exception of the patient, all citizens have an initial asset of 120,000 $ (equals to a yearly income at full work ability). In addition to this, the Health minister as well as the representative from the pharma company (you) expect both a fix salary of 120,000 $. The two payers will receive the same income of 120,000 $ but will have to share the health care costs, deducted from their yearly income. The two investors have no fix income but will share the revenue generated with the reimbursed pharmaceuticals.

The current standard treatment costs **50,000 $** per therapy and patient, paid by the health insurance.
In consequence each of the two payers earns currently 95,000 $ (= 120,000 – 50,000 / 2) and each of the two investors 25,000 (= 50,000 / 2).

These page timer metrics will not be displayed to the recipient.
First Click: *0 seconds*
Last Click: *0 seconds*
Page Submit: *0 seconds*
Click Count: *0 clicks*

You have now the option to participate in a short training (4 decisions), before starting the actual experiment. We encourage you to so. For the thorough completion of the training you will be awarded with additional US$ 0.6 after the experiment.

- YES, please start the training
- NO, I prefer to skip the training

These page timer metrics will not be displayed to the recipient.
First Click: *0 seconds*
Last Click: *0 seconds*
Page Submit: *0 seconds*
Click Count: *0 clicks*

Training round 1 (of 4):

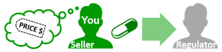

Your company developed a new treatment which prolongs the survival of the patient by **six** months (compared to no treatment), increasing the life expectancy by **one** additional month compared to the current standard treatment. The treatment does not increase the quality of life compared to the standard treatment.

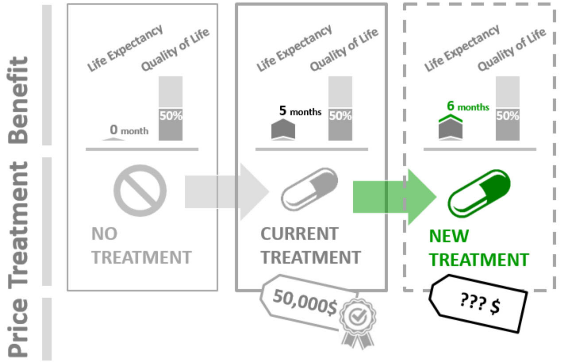

 This work is licensed under a Creative Commons Attribution 4.0 International License. Survey created with Qualtrics. 27.09.2020, 15:05

You have to prepare an offer for the Health minister shortly. Before you enter into a negotiation, you should decide on the **absolute minimum price, which you would still consider reasonable and fair for the new product:**

Your minimum price of ___,000 $ leads to the following state of the society:			
in thousand Dollars	**New Benefit**	**Compared to Current State**	**New Asset (Benefit + Initial Asset)**
**Patient**	30	+5	30
**2 Payers**			
**2 Investors**			
**Seller**	120	0	240
**Regulator**	120	0	240

[illegible]

Please select your minimum price by moving the red slider below.
You will see the related consequences in the table above.
If the values do not change, please klick the red slider again.

These page timer metrics will not be displayed to the recipient.
First Click: *0 seconds*
Last Click: *0 seconds*
Page Submit: *0 seconds*
Click Count: *0 clicks*

Training round 2 (of 4):

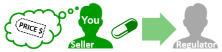

Now your company developed a new treatment which prolongs the survival of the patient by **nine** months (compared to no treatment), increasing the life expectancy by an additional **four** months compared to the current standard treatment. The treatment does not increase the quality of life compared to the standard treatment.

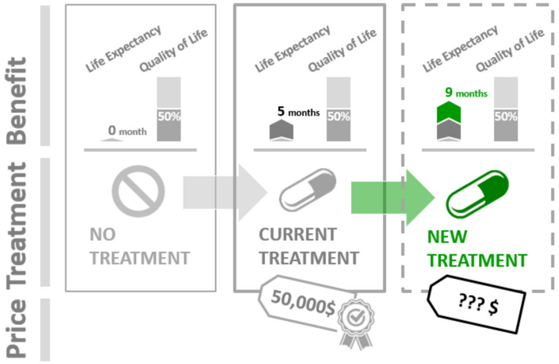

You have to prepare an offer for the Health minister shortly. Before you enter into a negotiation, you should decide on the **absolute minimum price, which you would still consider reasonable and fair for the new product:**

 This work is licensed under a Creative Commons Attribution 4.0 International License. Survey created with Qualtrics. 27.09.2020, 15:05

Your minimum price of ___,000 $ leads to the following state of the society:			
in thousand Dollars	New Benefit	Compared to Current State	New Asset (Benefit + Initial Asset)
**Patient**	45	+20	45
**2 Payers**			
**2 Investors**			
**Seller**	120	0	240
**Regulator**	120	0	240

[illegible]

Please select <u>your minimum price</u> by moving the red slider below.
You will see the related consequences in the table above.
If the values do not change, please klick the red slider again.

Your previous decision:
6 months: ${q://QID202/ChoiceNumericEntryValue/1},000

These page timer metrics will not be displayed to the recipient.
First Click: *0 seconds*
Last Click: *0 seconds*
Page Submit: *0 seconds*
Click Count: *0 clicks*

Training round 3 (of 4):

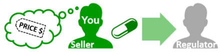

Now your company developed a new treatment which prolongs the survival of the patient by **thirteen** months (compared to no treatment), increasing the life expectancy by an additional **eight** months compared to the current standard treatment. The treatment does not increase the quality of life compared to the standard treatment.

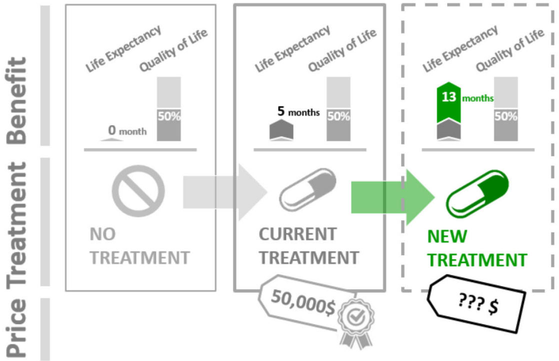

You have to prepare an offer for the Health minister shortly. Before you enter into a negotiation, you should decide on the **absolute minimum price, which you would still consider reasonable and fair for the new product:**

This work is licensed under a Creative Commons Attribution 4.0 International License. Survey created with Qualtrics.

Your maximum price of ___,000 $ leads to the following state of the society:			
in thousand Dollars	New Benefit	Compared to Current State	New Asset (Benefit + Initial Asset)
**Patient**	65	+40	65
**2 Payers**			
**2 Investors**			
**Seller**	120	0	240
**Regulator**	120	0	240

[illegible]

Please select <u>your minimum price</u> by moving the red slider below.
You will see the related consequences in the table above.
If the values do not change, please klick the red slider again.

Your previous decision:
6 months: ${q://QID202/ChoiceNumericEntryValue/1},000
9 months: ${q://QID206/ChoiceNumericEntryValue/1},000

These page timer metrics will not be displayed to the recipient.
First Click: *0 seconds*
Last Click: *0 seconds*
Page Submit: *0 seconds*
Click Count: *0 clicks*

Training round 4 (of 4):

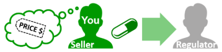

Now your company developed a new treatment which prolongs the survival of the patient by **fourteen** months (compared to no treatment), increasing the life expectancy by an additional **nine** months compared to the current standard treatment. The treatment does not increase the quality of life compared to the standard treatment.

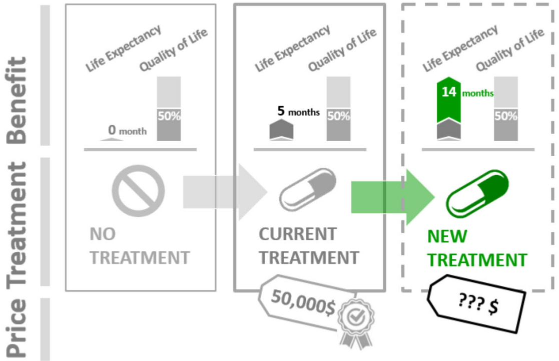

You have to prepare an offer for the Health minister shortly. Before you enter into a negotiation, you should decide on the **absolute minimum price, which you would still consider reasonable and fair for the new product:**

This work is licensed under a Creative Commons Attribution 4.0 International License. Survey created with Qualtrics.

Your minimum price of ___,000 $ leads to the following state of the society:			
in thousand Dollars	**New Benefit**	**Compared to Current State**	**New Asset (Benefit + Initial Asset)**
**Patient**	70	+45	70
**2 Payers**			
**2 Investors**			
**Seller**	120	0	240
**Regulator**	120	0	240

[illegible]

Please select <u>your minimum price</u> by moving the red slider below.
You will see the related consequences in the table above.
If the values do not change, please klick the red slider again.

Your previous decision:
6 months: ${q://QID202/ChoiceNumericEntryValue/1},000
9 months: ${q://QID206/ChoiceNumericEntryValue/1},000
13 months: ${q://QID210/ChoiceNumericEntryValue/1},000

These page timer metrics will not be displayed to the recipient.
First Click: *0 seconds*
Last Click: *0 seconds*
Page Submit: *0 seconds*
Click Count: *0 clicks*

You have successfully completed the training. Thank you very much!

These page timer metrics will not be displayed to the recipient.
First Click: *0 seconds*
Last Click: *0 seconds*
Page Submit: *0 seconds*
Click Count: *0 clicks*

Regulator - Decisions

Let us start with the experiment!

These page timer metrics will not be displayed to the recipient.
First Click: *0 seconds*
Last Click: *0 seconds*
Page Submit: *0 seconds*
Click Count: *0 clicks*

Decision situation (1 of 5):

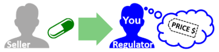

The Pharma Company offers a new pharmaceutical treatment which prolongs the survival of the patient by **eight** months (compared to no treatment), increasing the life expectancy by an additional **three** months compared to the current standard therapy. The treatment does not increase the quality of life compared to the standard treatment.

This work is licensed under a Creative Commons Attribution 4.0 International License. Survey created with Qualtrics.

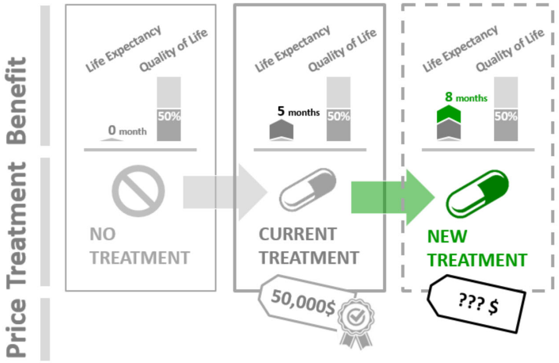

You expect the offer from the pharma company shortly. Before you enter into a negotiation, you should decide on the **absolute maximum price, which you would still consider reasonable and fair for the new product:**

Your maximum price of ___,000 $ leads to the following state of the society:			
in thousand Dollars	**New Benefit**	**Compared to Current State**	**New Asset (Benefit + Initial Asset)**
**Patient**	40	+15	40
**2 Payers**			
**2 Investors**			
**Seller**	120	0	240
**Regulator**	120	0	240

[illegible]

Please select <u>your maximum price</u> by moving the red slider below.
You will see the related consequences in the table above.
If the values do not change, please klick the red slider again.

These page timer metrics will not be displayed to the recipient.
First Click: *0 seconds*
Last Click: *0 seconds*
Page Submit: *0 seconds*
Click Count: *0 clicks*

Decision situation (2 of 5):

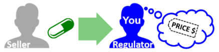

Now the Pharma Company offers a new pharmaceutical treatment which prolongs the survival of the patient by **ten** months (compared to no treatment), increasing the life expectancy by an additional **five** months compared to the current standard treatment. The treatment does not increase the quality of life compared to the standard treatment.

This work is licensed under a Creative Commons Attribution 4.0 International License. Survey created with Qualtrics.

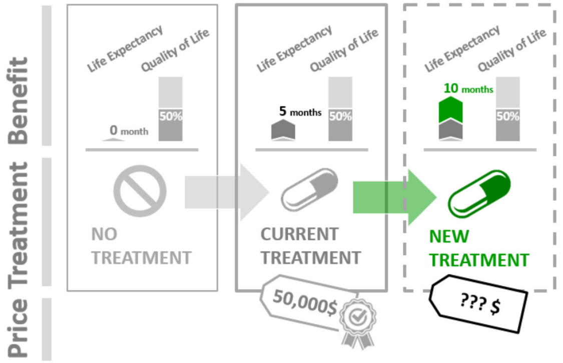

You expect the offer from the pharma company shortly. Before you enter into a negotiation, you should decide on the **absolute maximum price, which you would still consider reasonable and fair for the new product:**

Your maximum price of ___,000 $ leads to the following state of the society:			
in thousand Dollars	**New Benefit**	**Compared to Current State**	**New Asset (Benefit + Initial Asset)**
**Patient**	50	+25	50
**2 Payers**			
**2 Investors**			
**Seller**	120	0	240
**Regulator**	120	0	240

[illegible]

Please select <u>your maximum price</u> by moving the red slider below. You will see the related consequences in the table above. *If the values do not change, please klick the red slider again.*

Your previous decision:
8 months: ${q://QID116/ChoiceNumericEntryValue/1},000

These page timer metrics will not be displayed to the recipient.
First Click: *0 seconds*
Last Click: *0 seconds*
Page Submit: *0 seconds*
Click Count: *0 clicks*

Decision situation (3 of 5):

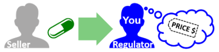

Now the Pharma Company offers a new pharmaceutical treatment which prolongs the survival of the patient by **twelve** months (compared to no treatment), increasing the life expectancy by an additional **seven** months compared to the current standard treatment. The treatment does not increase the quality of life compared to the standard treatment.

This work is licensed under a Creative Commons Attribution 4.0 International License. Survey created with Qualtrics.

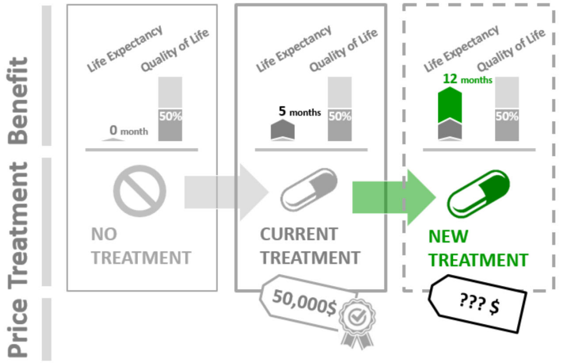

You expect the offer from the pharma company shortly. Before you enter into a negotiation, you should decide on the **absolute maximum price, which you would still consider reasonable and fair for the new product:**

Your maximum price of ___,000 $ leads to the following state of the society:			
in thousand Dollars	**New Benefit**	**Compared to Current State**	**New Asset (Benefit + Initial Asset)**
**Patient**	60	+35	60
**2 Payers**			
**2 Investors**			
**Seller**	120	0	240
**Regulator**	120	0	240

[illegible]

Please select <u>your maximum price</u> by moving the red slider below. You will see the related consequences in the table above. *If the values do not change, please klick the red slider again.*

Your previous decisions:
8 months: ${q://QID116/ChoiceNumericEntryValue/1},000
10 months: ${q://QID117/ChoiceNumericEntryValue/1},000

These page timer metrics will not be displayed to the recipient.
First Click: *0 seconds*
Last Click: *0 seconds*
Page Submit: *0 seconds*
Click Count: *0 clicks*

Decision situation (4 of 5):

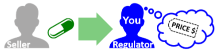

Now the Pharma Company offers a new pharmaceutical treatment which prolongs the survival of the patient by **fifteen** months (compared to no treatment), increasing the life expectancy by an additional **ten** months compared to the current standard treatment. The treatment does not increase the quality of life compared to the standard treatment.

This work is licensed under a Creative Commons Attribution 4.0 International License. Survey created with Qualtrics.

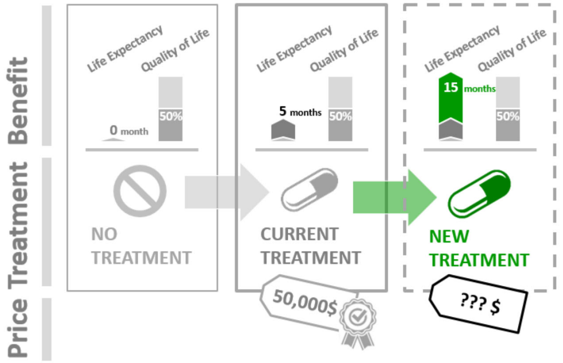

You expect the offer from the pharma company shortly. Before you enter into a negotiation, you should decide on the **absolute maximum price, which you would still consider reasonable and fair for the new product:**

Your maximum price of ___,000 $ leads to the following state of the society:			
in thousand Dollars	**New Benefit**	**Compared to Current State**	**New Asset (Benefit + Initial Asset)**
**Patient**	75	+50	75
**2 Payers**			
**2 Investors**			
**Seller**	120	0	240
**Regulator**	120	0	240

[illegible]

Please select your maximum price by moving the red slider below.
You will see the related consequences in the table above.
If the values do not change, please klick the red slider again.

Your previous decisions:
8 months: ${q://QID116/ChoiceNumericEntryValue/1},000
10 months: ${q://QID117/ChoiceNumericEntryValue/1},000
12 months: ${q://QID118/ChoiceNumericEntryValue/1},000

These page timer metrics will not be displayed to the recipient.
First Click: *0 seconds*
Last Click: *0 seconds*
Page Submit: *0 seconds*
Click Count: *0 clicks*

Decision situation (5 of 5):

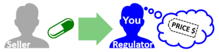

Now the Pharma Company offers a new pharmaceutical treatment which prolongs the survival of the patient by **seventeen** months (compared to no treatment), increasing the life expectancy by an additional **twelve** months compared to the current standard treatment. The treatment does not increase the quality of life compared to the standard treatment.

This work is licensed under a Creative Commons Attribution 4.0 International License. Survey created with Qualtrics.

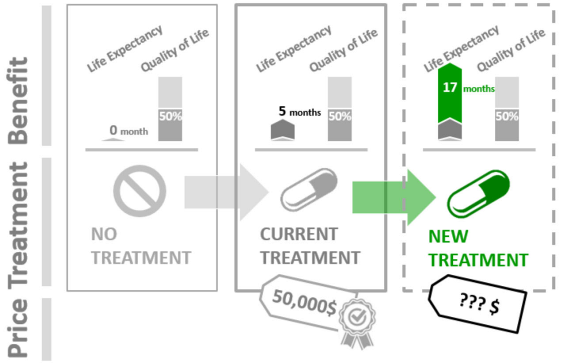

You expect the offer from the pharma company shortly. Before you enter into a negotiation, you should decide on the **absolute maximum price, which you would still consider reasonable and fair for the new product:**

Your maximum price of ___,000 $ leads to the following state of the society:			
in thousand Dollars	**New Benefit**	**Compared to Current State**	**New Asset (Benefit + Initial Asset)**
**Patient**	85	+60	85
**2 Payers**			
**2 Investors**			
**Seller**	120	0	240
**Regulator**	120	0	240

[illegible]

Please select your maximum price by moving the red slider below.
You will see the related consequences in the table above.
If the values do not change, please klick the red slider again.

Your previous decisions:
8 months: ${q://QID116/ChoiceNumericEntryValue/1},000
10 months: ${q://QID117/ChoiceNumericEntryValue/1},000
12 months: ${q://QID118/ChoiceNumericEntryValue/1},000
15 months: ${q://QID119/ChoiceNumericEntryValue/1},000

These page timer metrics will not be displayed to the recipient.
First Click: *0 seconds*
Last Click: *0 seconds*
Page Submit: *0 seconds*
Click Count: *0 clicks*

For the past five rounds: please indicate in descending order the relevance of the stakeholders for your decision.
You can drag and drop the options below to bring them in your preferred order.

Premium Payers
Seller
Health Minister
Patient

This work is licensed under a Creative Commons Attribution 4.0 International License. Survey created with Qualtrics.

Investors

These page timer metrics will not be displayed to the recipient.
First Click: *0 seconds*
Last Click: *0 seconds*
Page Submit: *0 seconds*
Click Count: *0 clicks*

Transition Screen Regulator->Seller

Now let us assume that you have accepted a job offer from the Pharmaceutical Company as their new **Seller**. In consequence, you will ***switch roles*** for the upcoming rounds. You will be responsible to sell new products to the Health Minister of this country.

Before you enter into a negotiation, you should decide on the **absolute *minimum* price, which you would still consider reasonable and fair for your new product**. Bellow this “walk-away price” you would never agree to sell the new pharmaceutical in this country.

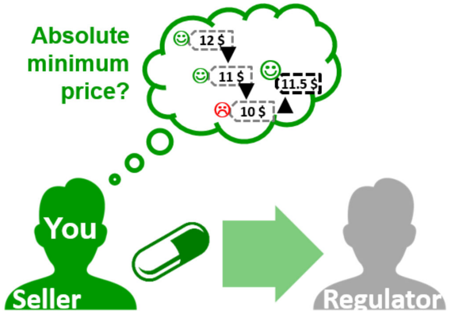

These page timer metrics will not be displayed to the recipient.
First Click: *0 seconds*
Last Click: *0 seconds*
Page Submit: *0 seconds*
Click Count: *0 clicks*

Seller - Decisions

Let us start with the experiment!

These page timer metrics will not be displayed to the recipient.
First Click: *0 seconds*
Last Click: *0 seconds*
Page Submit: *0 seconds*
Click Count: *0 clicks*

Decision situation (1 of 5):

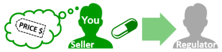

Your company developed a new treatment which prolongs the survival of the patient by **eight** months (compared to no treatment), increasing the life expectancy by an additional **three** months compared to the current standard treatment. The treatment does not increase the quality of life compared to the standard treatment.

This work is licensed under a Creative Commons Attribution 4.0 International License. Survey created with Qualtrics.

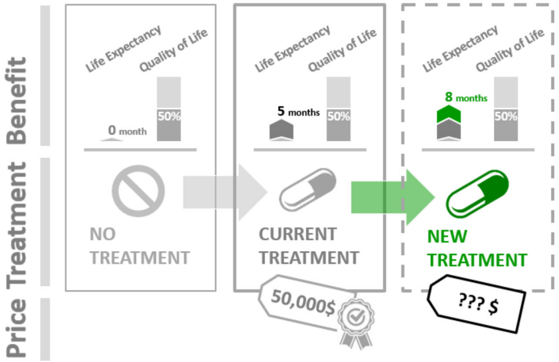

You have to prepare an offer for the Health minister shortly. Before you enter into a negotiation, you should decide on the **absolute minimum price, which you would still consider reasonable and fair for the new product:**

Your minimum price of ___,000 $ leads to the following state of the society:			
in thousand Dollars	**New Benefit**	**Compared to Current State**	**New Asset (Benefit + Initial Asset)**
**Patient**	40	+15	40
**2 Payers**			
**2 Investors**			
**Seller**	120	0	240
**Regulator**	120	0	240

[illegible]

Please select your minimum price by moving the red slider below.
You will see the related consequences in the table above.
If the values do not change, please klick the red slider again.

These page timer metrics will not be displayed to the recipient.
First Click: *0 seconds*
Last Click: *0 seconds*
Page Submit: *0 seconds*
Click Count: *0 clicks*

Decision situation (2 of 5):

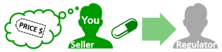

Now your company developed a new treatment which prolongs the survival of the patient by **ten** months (compared to no treatment), increasing the life expectancy by an additional **five** months compared to the current standard treatment. The treatment does not increase the quality of life compared to the standard treatment.

This work is licensed under a Creative Commons Attribution 4.0 International License. Survey created with Qualtrics.

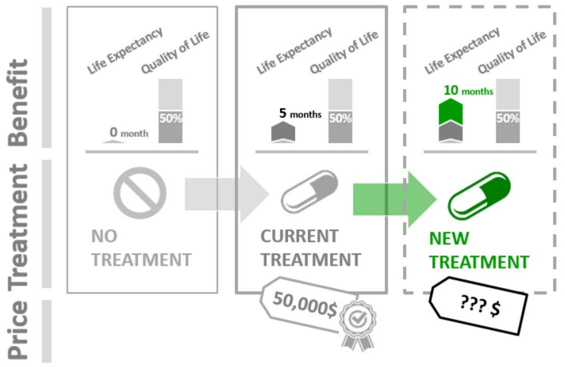

You have to prepare an offer for the Health minister shortly. Before you enter into a negotiation, you should decide on the **absolute minimum price, which you would still consider reasonable and fair for the new product:**

Your minimum price of ___,000 $ leads to the following state of the society:			
in thousand Dollars	**New Benefit**	**Compared to Current State**	**New Asset (Benefit + Initial Asset)**
**Patient**	50	+25	50
**2 Payers**			
**2 Investors**			
**Seller**	120	0	240
**Regulator**	120	0	240

[illegible]

Please select your minimum price by moving the red slider below.
You will see the related consequences in the table above.
If the values do not change, please klick the red slider again.

Your previous decision:
8 months: ${q://QID112/ChoiceNumericEntryValue/1},000

These page timer metrics will not be displayed to the recipient.
First Click: *0 seconds*
Last Click: *0 seconds*
Page Submit: *0 seconds*
Click Count: *0 clicks*

Decision situation (3 of 5):

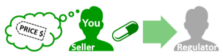

Now your company developed a new treatment which prolongs the survival of the patient by **twelve** months (compared to no treatment), increasing the life expectancy by an additional **seven** months compared to the current standard treatment. The treatment does not increase the quality of life compared to the standard treatment.

This work is licensed under a Creative Commons Attribution 4.0 International License. Survey created with Qualtrics.

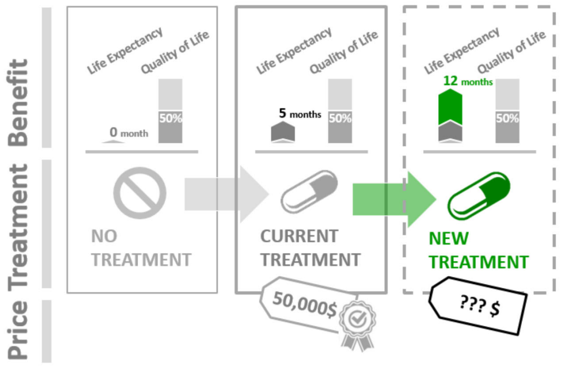

You have to prepare an offer for the Health minister shortly. Before you enter into a negotiation, you should decide on the **absolute minimum price, which you would still consider reasonable and fair for the new product:**

Your maximum price of ___,000 $ leads to the following state of the society:			
in thousand Dollars	**New Benefit**	**Compared to Current State**	**New Asset (Benefit + Initial Asset)**
**Patient**	60	+35	60
**2 Payers**			
**2 Investors**			
**Seller**	120	0	240
**Regulator**	120	0	240

[illegible]

Please select your minimum price by moving the red slider below.
You will see the related consequences in the table above.
If the values do not change, please klick the red slider again.

Your previous decisions:
8 months: ${q://QID112/ChoiceNumericEntryValue/1},000
10 months: ${q://QID109/ChoiceNumericEntryValue/1},000

These page timer metrics will not be displayed to the recipient.
First Click: *0 seconds*
Last Click: *0 seconds*
Page Submit: *0 seconds*
Click Count: *0 clicks*

Decision situation (4 of 5):

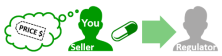

Now your company developed a new treatment which prolongs the survival of the patient by **fifteen** months (compared to no treatment), increasing the life expectancy by an additional **ten** months compared to the current standard treatment. The treatment does not increase the quality of life compared to the standard treatment.

This work is licensed under a Creative Commons Attribution 4.0 International License. Survey created with Qualtrics.

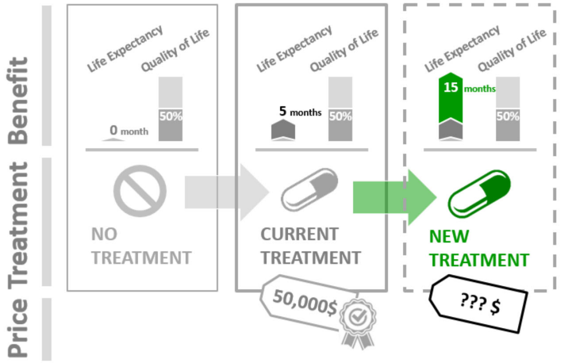

You have to prepare an offer for the Health minister shortly. Before you enter into a negotiation, you should decide on the **absolute minimum price, which you would still consider reasonable and fair for the new product:**

Your minimum price of ___,000 $ leads to the following state of the society:			
in thousand Dollars	**New Benefit**	**Compared to Current State**	**New Asset (Benefit + Initial Asset)**
**Patient**	75	+50	75
**2 Payers**			
**2 Investors**			
**Seller**	120	0	240
**Regulator**	120	0	240

[illegible]

Please select your minimum price by moving the red slider below.
You will see the related consequences in the table above.
If the values do not change, please klick the red slider again.

Your previous decisions:
8 months: ${q://QID112/ChoiceNumericEntryValue/1},000
10 months: ${q://QID109/ChoiceNumericEntryValue/1},000
12 months: ${q://QID113/ChoiceNumericEntryValue/1},000

These page timer metrics will not be displayed to the recipient.
First Click: *0 seconds*
Last Click: *0 seconds*
Page Submit: *0 seconds*
Click Count: *0 clicks*

Decision situation (5 of 5):

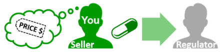

Now your company developed a new treatment which prolongs the survival of the patient by **seventeen** months (compared to no treatment), increasing the life expectancy by an additional **twelve** months compared to the current standard treatment. The treatment does not increase the quality of life compared to the standard treatment.

This work is licensed under a Creative Commons Attribution 4.0 International License. Survey created with Qualtrics.

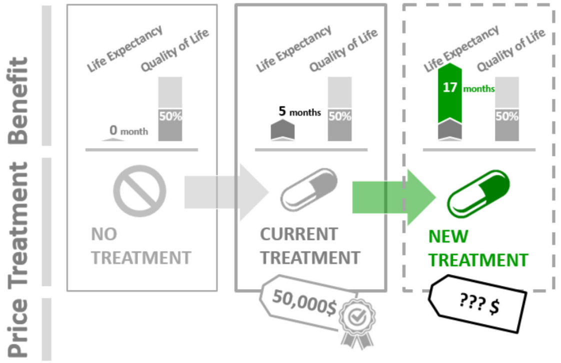

You have to prepare an offer for the Health minister shortly. Before you enter into a negotiation, you should decide on the **absolute minimum price, which you would still consider reasonable and fair for the new product:**

Your minimum price of ___,000 $ leads to the following state of the society:			
in thousand Dollars	**New Benefit**	**Compared to Current State**	**New Asset (Benefit + Initial Asset)**
**Patient**	85	+60	85
**2 Payers**			
**2 Investors**			
**Seller**	120	0	240
**Regulator**	120	0	240

[illegible]

Please select your minimum price by moving the red slider below.
You will see the related consequences in the table above.
If the values do not change, please klick the red slider again.

Your previous decisions:
8 months: ${q://QID112/ChoiceNumericEntryValue/1},000
10 months: ${q://QID109/ChoiceNumericEntryValue/1},000
12 months: ${q://QID113/ChoiceNumericEntryValue/1},000
15 months: ${q://QID114/ChoiceNumericEntryValue/1},000

These page timer metrics will not be displayed to the recipient.
First Click: *0 seconds*
Last Click: *0 seconds*
Page Submit: *0 seconds*
Click Count: *0 clicks*

For the past five rounds: please indicate in descending order the relevance of the stakeholders for your decision.
You can drag and drop the options below to bring them in your preferred order.

Premium Payers

Patient

Health Minister

Seller

This work is licensed under a Creative Commons Attribution 4.0 International License. Survey created with Qualtrics.

Investors

These page timer metrics will not be displayed to the recipient.
First Click: *0 seconds*
Last Click: *0 seconds*
Page Submit: *0 seconds*
Click Count: *0 clicks*

Transition Screen Seller->Regulator

Now let us assume that you have accepted a job offer from the Government to become the new **Health Minister** in this country. In consequence, you will ***switch roles*** for the upcoming rounds. You you will receive offers from a pharma company for the reimbursement of new pharmaceuticals.

Before you enter into a negotiation, you should decide on the **absolute *maximum* price, which you would still consider reasonable and fair for the new product**. Above this "walk-away price" you would never allow the new pharmaceutical to be reimbursed by the public health insurance.

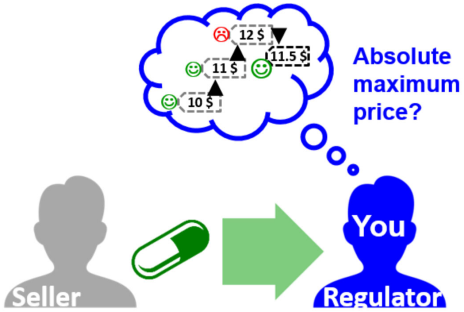

These page timer metrics will not be displayed to the recipient.
First Click: *0 seconds*
Last Click: *0 seconds*
Page Submit: *0 seconds*
Click Count: *0 clicks*

Closing

We know that this was not an easy task. It might have been difficult for you to find a reasonable price in the described situation. Please indicate which information helped you most (most important) and which information was less relevant for your decisions (relevance in descending order).
You can drag and drop the options below to bring them in your preferred order.

SURVIVAL benefit for the patient
QUALITY of life of the patient
Comparison to REAL world prices (newspaper, personal experience, ...)
Comparison to your submitted PRICES in PREVIOUS rounds
Resulting economic BENEFIT for the different stakeholders
Resulting ASSET for the different stakeholders
COMPARISON (change) of the economic BENEFIT to current state

 This work is licensed under a Creative Commons Attribution 4.0 International License. Survey created with Qualtrics.

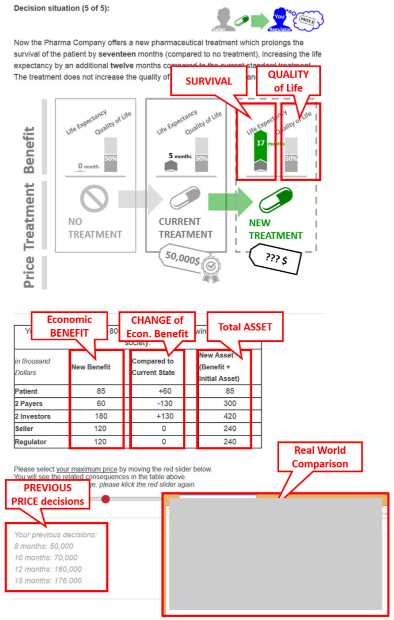

[Screenshot to a newspaper article headline removed for publication.]

These page timer metrics will not be displayed to the recipient.
First Click: *0 seconds*
Last Click: *0 seconds*
Page Submit: *0 seconds*
Click Count: *0 clicks*

Check the category on the right that conforms to the frequency with which you have carried out the following acts in the last year:

	Never	Once	More than once	Often	Very often
1. I have given money to a charity.	○	○	○	○	○
2. I have given money to a stranger who needed it (or asked me for it).	○	○	○	○	○
3. I have donated goods or clothes to a charity.	○	○	○	○	○
4. I have done volunteer work for a charity.	○	○	○	○	○
5. I have donated blood.	○	○	○	○	○
6. I have helped carry a stranger's belongings (books, parcels, etc.).	○	○	○	○	○
7. I have allowed someone to go ahead of me in a lineup (at photocopy machine, in the supermarket).	○	○	○	○	○
8. I have pointed out a clerk's error (in a bank, at the supermarket) in undercharging me for an item.	○	○	○	○	○
9. I have offered my seat on a bus or train to a stranger who was standing.	○	○	○	○	○
10. I have helped an acquaintance to move households.	○	○	○	○	○

These page timer metrics will not be displayed to the recipient.
First Click: *0 seconds*
Last Click: *0 seconds*
Page Submit: *0 seconds*
Click Count: *0 clicks*

 This work is licensed under a Creative Commons Attribution 4.0 International License. Survey created with Qualtrics.

Please answer with yes or no:
Remember: All your responses in this experiment are anonymous.

	yes	no	I don't want to answer
Have you ever been affected by a chronic and/or severe disease (but not now)?	○	○	○
Do you personally suffer from a chronic and/or severe disease (currently under treatment)?	○	○	○
Are people close to you (family or friends) suffering from a severe and/or chronic disease?	○	○	○
Have you ever discussed with a person close to you (family or friend) who suffers from a chronic disease his or her condition?	○	○	○
Have you or people close to you (family or friends) ever needed pharmaceutical treatment for a longer period?	○	○	○

These page timer metrics will not be displayed to the recipient.
First Click: *0 seconds*
Last Click: *0 seconds*
Page Submit: *0 seconds*
Click Count: *0 clicks*

What is your educational background:

What is your current employment status?

In which industry have you been working in your current or most recent position?

These page timer metrics will not be displayed to the recipient.
First Click: *0 seconds*
Last Click: *0 seconds*
Page Submit: *0 seconds*
Click Count: *0 clicks*

Have you ever been employed by a... (multiple answers possible)

- ☐ Health care provider (hospital, clinic, elderly home, or similar) ?
- ☐ Public authority, responsible for health care (e.g. FDA) ?
- ☐ Pharmaceutical company or related?
- ☐ Medical device company?
- ☐ Pharmacy, drugstore or wholesale company focusing on health care?
- ☐ Health insurance or health plan?
- ☐ Patient organization?
- ☐ Research organization focusing on health care?
- ☐ Any other organization involved in or focusing on health care?
- ☐ No (none of above)

These page timer metrics will not be displayed to the recipient.
First Click: *0 seconds*
Last Click: *0 seconds*
Page Submit: *0 seconds*
Click Count: *0 clicks*

Do you have any feedback to the researchers regarding this survey?

This work is licensed under a Creative Commons Attribution 4.0 International License. Survey created with Qualtrics.

These page timer metrics will not be displayed to the recipient.
First Click: *0 seconds*
Last Click: *0 seconds*
Page Submit: *0 seconds*
Click Count: *0 clicks*

Powered by Qualtrics

This work is licensed under a Creative Commons Attribution 4.0 International License. Survey created with Qualtrics.
