# Supplementary material for: Assessing social preferences in reimbursement negotiations for new Pharmaceuticals in Oncology: an experimental design to analyse willingness to pay and willingness to accept
Source: BMC Health Serv Res. 2021 Mar 16;21:234. doi: 10.1186/s12913-021-06231-8 (PMC7968195; doi:10.1186/s12913-021-06231-8)
Supplement: Supplementary file 4 — Additional file 4. Complete instructions for the second run of the experiment. [file 12913_2021_6231_MOESM4_ESM.pdf]

This questionnaire has already been referenced in: Wettstein, D.J., Boes, S.: The impact of reimbursement negotiations on cost and availability of new pharmaceuticals: evidence from an online experiment. Health Econ Rev 10(1), 13 (2020). https://doi.org/10.1186/s13561-020-00267-y

Intro_1

This survey is part of a scientific study interested in understanding preferences for new health technologies. You will be asked to assign monetary values to new medical treatments.

The experiment will take between 20 and 25 minutes. It will start with a detailed description of the decision situation, followed by an introductory training of four rounds. The core part of the experiment is split in two sets of five rounds each. You will receive a fix salary of US$ 2.40 for thorough completion of the experiment plus a potential bonus.
This potential bonus payments will be calculated after the experiment has been closed and processed within 10 days.

The first five rounds require your thorough reflection and honest answers only. In the second set, you will be able to collect a bonus in each round, depending on your decision and the decision of another player.

Your decisions in all rounds will potentially affect other MTurk workers and people in the real world via monetary payoffs.

Your participation is anonymous to the responsible researchers; they will not be able to draw conclusions to you as an individual. The data provided during the survey will be used for scientific purposes only.

Please indicate whether you want to participate in this study:

- Yes
- No

Are you 18+ years old?

- Yes
- No

Are you resident in the USA?

- Yes
- No

These page timer metrics will not be displayed to the recipient.
First Click: *0 seconds*
Last Click: *0 seconds*
Page Submit: *0 seconds*
Click Count: *0 clicks*

Intro_2

Dear participant

Thank you very much in advance for your willingness to contribute to our research!

This study was designed by the University of Lucerne (Switzerland) and carried out in cooperation with the Decision Science Laboratory of the ETH Zurich (Switzerland).

Please read carefully the instructions on the following pages.

These page timer metrics will not be displayed to the recipient.
First Click: *0 seconds*
Last Click: *0 seconds*
Page Submit: *0 seconds*
Click Count: *0 clicks*

May we first ask some basic information about you.

Please select your year of birth.

 This work is licensed under a Creative Commons Attribution 4.0 International License. Survey created with Qualtrics.

Please select your gender.

- Male
- Female
- Other

These page timer metrics will not be displayed to the recipient.
First Click: *0 seconds*
Last Click: *0 seconds*
Page Submit: *0 seconds*
Click Count: *0 clicks*

The objective of this research is to find out the "true" value that you assign to a new health technology in comparison to an existing ("standard") technology.
You are given in each decision situation complete information on how all relevant stakeholders are affected.

Please assume for the following experiment that you belong to a country with in total seven citizens: one *Patient*, one *Regulator (representing the Government)*, one *Seller (representing a pharmaceutical company)*, two *Premium Payers (who finance the public health insurance)* and two *Investors (who financed a new pharmaceutical)*.

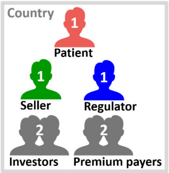

The Patient is suffering from a severe disease: a deadly blood cancer. He/she is under treatment with an existing therapy (current standard of care): a pharmaceutical product with a known benefit to the patient (life expectancy and quality of life).
The Regulator is the responsible "Health Minister" and represents the government. He/she is in charge of regulating prices for pharmaceuticals. Only pharmaceuticals with an official list price are eligible for payment by the public health insurance.
The Seller represents an international pharmaceutical company that developed a new product to treat the patient's disease. He/she is in charge of negotiating with the government an officially reimbursed price.
The Premium Payers finance the public health insurance. Insurance is mandatory and the collected premiums are the only financial source to pay the treatment for any patients in need. However, only treatments approved by the government for reimbursement are covered. If health expenditures are lower than the actual premiums the payers benefit. If the expenditures are higher, they will have to eat up their savings or incur dept.
The Investors have invested their savings in the past into a research-based pharmaceutical company. They expect a return on their investment, which compensates them for the additional risk they took, compared to a "risk-free" investment in a government bond for example.

These page timer metrics will not be displayed to the recipient.
First Click: *0 seconds*
Last Click: *0 seconds*
Page Submit: *0 seconds*
Click Count: *0 clicks*

The **Seller** offers the new treatment at a proposed price, which has to be approved by the government (**Regulator**). If the **Regulator** considers the price as to high, he/she will refuse to approve the product. Vice versa, if the **Seller** is confronted with a counterproposal (reduced price) considered too low, he/she will not introduce the product in this market.

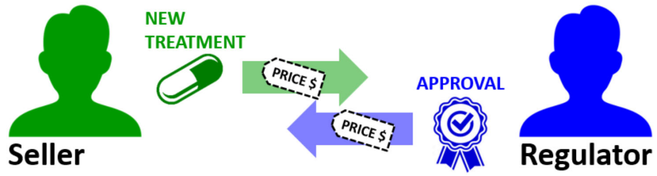

 This work is licensed under a Creative Commons Attribution 4.0 International License. Survey created with Qualtrics.

If both parties agree, the **Patient** will get access to the new treatment with all its benefits (life expectancy, quality of life). The **Investors** will in consequence receive the price (revenue) and the **Payers** will have to pay the price (cost).

As long as the regulator and the seller do not agree, the product will not be available, which means no additional benefit for the patient, no additional costs for the premium payers and no revenue for investors. The regulator and the seller are both employed and receive a fix salary.

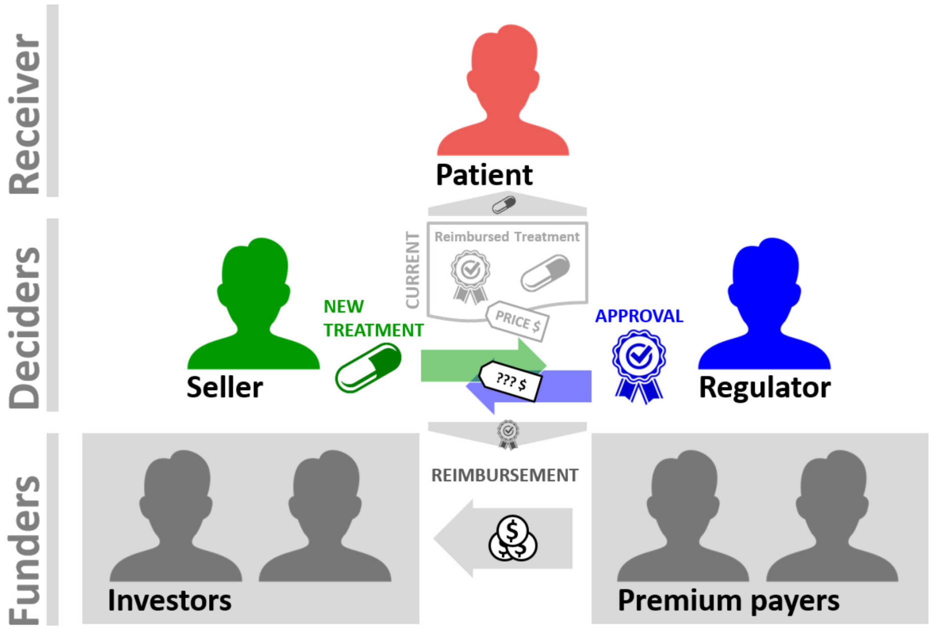

Control question 1: What happens, if the **seller** proposes the new product at a price much higher than the regulator wants to accept? Please select all correct answers below:

- ☐ The company will still sell the new product, since the patient pays for his treatment out-of-pocket in this country.
- ☐ The patient will not get access to the new treatment, since the new product will not be reimbursable by the public health insurance.
- ☐ The seller and the regulator will not receive their fix salary.
- ☐ The premium payers will have to pay for the new treatment at an unreasonable high price.
- ☐ The investors earn more money on their risky investment than they originally expected.

Control question 2: Would your answer be different if the **regulator** askes for a price of the new product lower than the lowest price the seller wants to accept?

Yes, this is different.	No, the effect is the same as in the question above.
○	○

These page timer metrics will not be displayed to the recipient.
First Click: *0 seconds*
Last Click: *0 seconds*
Page Submit: *0 seconds*
Click Count: *0 clicks*

Regulator - 100'000 - Intro & Training

You are the responsible **Regulator** ("Health Minister") in this country. In the following, **you will receive offers from a pharma company** for the reimbursement of new pharmaceuticals. You will see their expected benefit, based on clinical studies.

 This work is licensed under a Creative Commons Attribution 4.0 International License. Survey created with Qualtrics.

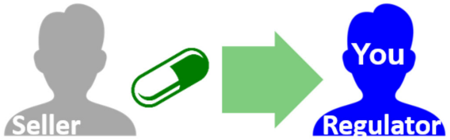

Before you enter into a negotiation, you should decide on the **absolute maximum price, which you would still consider reasonable and fair for the new product**. Above this "walk-away price" you would never allow the new pharmaceutical to be reimbursed by the public health insurance.

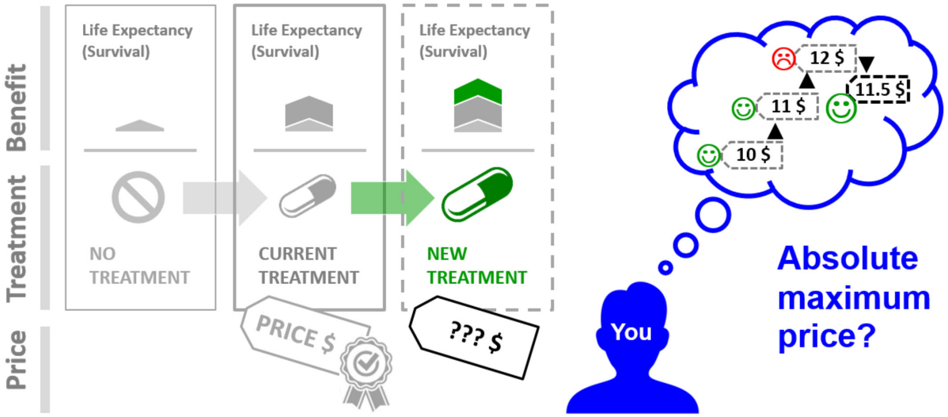

This part of the experiment focuses only on your private valuation of new health technologies. You do not have to reflect on any negotiation tactics or strategies. There is no "correct" answer; your decision should be based solely on your personal preferences.
Be aware that you will not be able to change your decisions after submission.

All prices are expressed in fictive "Dollar" ($) and trade at the end of the experiment at a currency rate of **100,000 $ = 1 US$**.

These page timer metrics will not be displayed to the recipient.
First Click: *0 seconds*
Last Click: *0 seconds*
Page Submit: *0 seconds*
Click Count: *0 clicks*

Control question 3: Let us assume that you are a real coffee lover (if not, you can replace coffee with soda or else). Meaning that you always benefit more from two cups than from one, more from a large cup than from a small one (see picture below). Let us further assume that 2 $ represents the maximum price you still consider reasonable and fair for a **small** cup of 12 oz.

a) Assuming all other things equal and that you have the money available and can afford it: Which price would represent your absolute maximum willingness to pay for a **medium** cup of 16 oz.?

- ○ A price higher than 2 $
- ○ A price of 2 $
- ○ A price lower than 2 $

---

b) Would you buy a large cup of 20 oz. for 2 $?

- ○ Yes
- ○ No

 This work is licensed under a Creative Commons Attribution 4.0 International License. Survey created with Qualtrics.

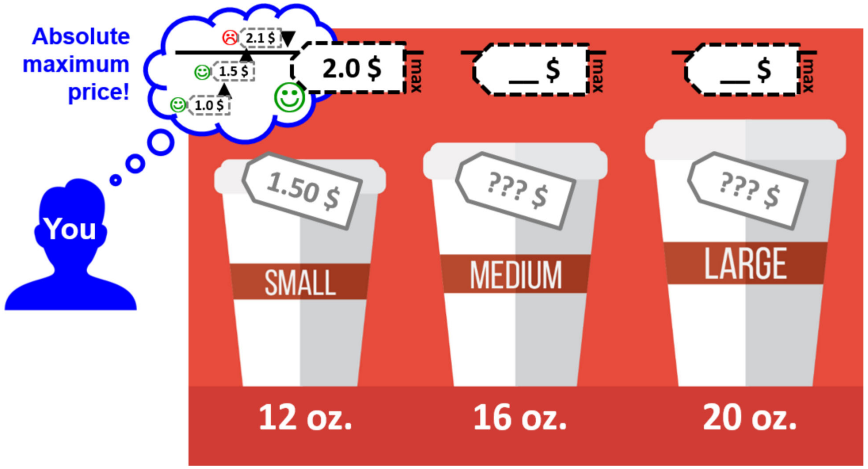

[Coffee cup adjusted from wikiHow: How to Order Coffee. wikiHow Inc.; 2019. The image is licensed under a Creative Commons Attribution-NonCommercial-ShareAlike 3.0 Unported License.]

These page timer metrics will not be displayed to the recipient.
First Click: *0 seconds*
Last Click: *0 seconds*
Page Submit: *0 seconds*
Click Count: *0 clicks*

Your decision will have real consequences on others. After this experiment is closed, you will be randomly paired with another MTurk worker who played the opposite role. The rounds will be implemented, depending on your own and your counterpart's decisions. Whenever an agreement would be possible, following payoffs will be made:

Patient: Benefit converted to US$ will be donated to the Leukemia & Lymphoma Society (LLS) which provides financial support for patients with blood cancer (https://www.lls.org/support/financial-support)
Payers: Positive benefit converted to US$ /10 will be paid to two other MTurk workers (randomly selected)
Investors: Positive benefit converted to US$ /10 will be paid to two other MTurk workers (randomly selected)
Regulator or **Seller**: Benefit converted to US$ will be paid to you and your negotiation partner. The fix salary for you both is guaranteed, regardless whether an agreement was possible (as long as the experiment was completed).

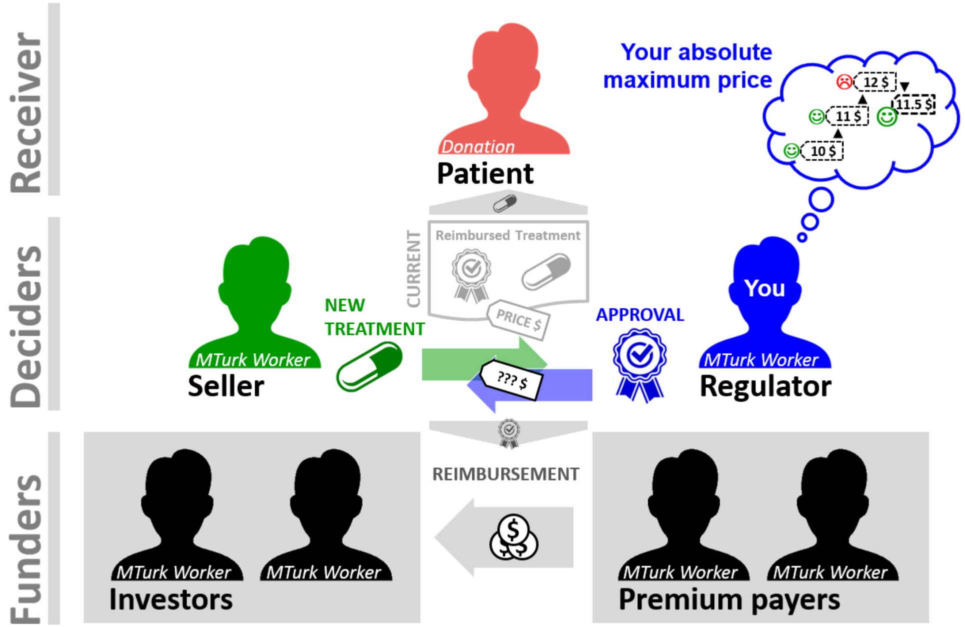

These page timer metrics will not be displayed to the recipient.
First Click: *0 seconds*
Last Click: *0 seconds*
Page Submit: *0 seconds*
Click Count: *0 clicks*

Initial position:

Patients suffer from a deadly, incurable blood cancer. With no treatment, they have a remaining life expectancy below one month. There is one pharmaceutical treatment available, which increases the patient's life expectancy (*survival*) by **five months** at an unchanged quality of life (*QoL*). The QoL is an experience-based, self-reported indicator for the patient's physical functioning, bodily pain, as well as mental, emotional and social functioning etc. It is measured at a scale from 0 to 100%. The lower the score the more disabled the patient. The QoL of the patient under current standard treatment is **50%**.

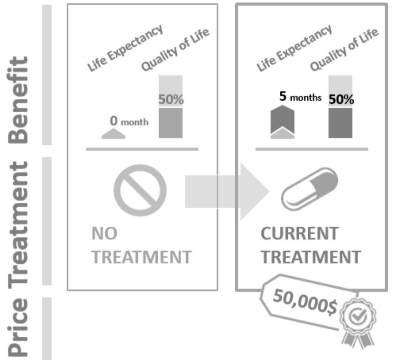

At full QoL (100%) the patient could realize a monthly income of 10,000 $. Due to the lower QoL the patient's work ability (productivity) is reduced proportionally. In consequence the potential income he/she can generate equals 10,000 $ * 50% = 5,000 $ per month. This translates into a total economic benefit for the patient under current standard therapy of 10,000 $ * **50%** * **5** months = 25,000 $.

This work is licensed under a Creative Commons Attribution 4.0 International License. Survey created with Qualtrics.

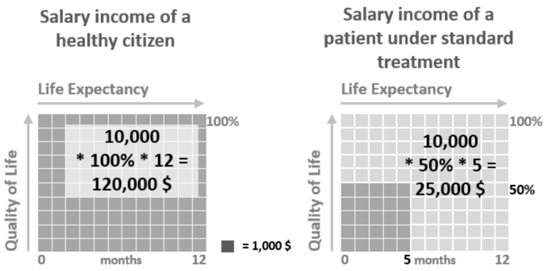

With exception of the patient, all citizens have an initial asset of 120,000 $ (equals to a yearly income at full work ability). In addition to this, the Health minister (you) as well as the representative from the pharma company expect both a fix salary of 120,000 $. The two payers will receive the same income of 120,000 $ but will have to share the health care costs, deducted from their yearly income. The two investors have no fix income but will share the revenue generated with the reimbursed pharmaceuticals.

The current standard treatment costs **50,000 $** per therapy and patient, paid by the health insurance.
In consequence each of the two payers earns currently 95,000 $ (= 120,000 – 50,000 / 2) and each of the two investors 25,000 $ (= 50,000 / 2).

Control question 4: If the patient's survival increases from zero to 4 months at the same quality of life (50%), how much will he/she gain "economically" in cash?

- ◯ Nothing, the benefit of survival cannot be expressed in monetary terms.
- ◯ Nothing, the patient cannot work at a reduced quality of life.
- ◯ 20,000 $
- ◯ 25,000 $
- ◯ 40,000 $

These page timer metrics will not be displayed to the recipient.
First Click: *0 seconds*
Last Click: *0 seconds*
Page Submit: *0 seconds*
Click Count: *0 clicks*

So much for the introduction and theory. Thank you for your patience so far!

Let us start with a short training (4 decisions), before we move to the actual experiment.

Training round 1 (of 4):

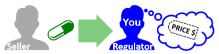

The Pharma Company offers a new pharmaceutical treatment which prolongs the survival of the patient by **six** months (compared to no treatment), increasing the life expectancy by **one** additional month compared to the current standard therapy. The treatment does not increase the quality of life compared to the standard treatment.

This work is licensed under a Creative Commons Attribution 4.0 International License. Survey created with Qualtrics.

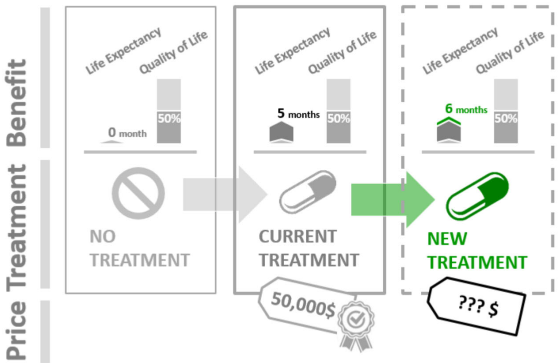

You expect the offer from the pharma company shortly. Before you enter into a negotiation, you should decide on the **absolute maximum price, which you would still consider reasonable and fair for the new product:**

Your maximum price of ___,000 $ leads to the following state of the society:			
in thousand Dollars	**New Benefit**	**Compared to Current State**	**New Asset (Benefit + Initial Asset)**
**Patient**	30	+5	30
**2 Payers**			
**2 Investors**			
**Seller**	120	0	240
**Regulator**	120	0	240

[illegible]

Please select your maximum price by moving the red slider below.
You will see the related consequences in the table above.
If the values do not change, please click the red slider again.

These page timer metrics will not be displayed to the recipient.
First Click: *0 seconds*
Last Click: *0 seconds*
Page Submit: *0 seconds*
Click Count: *0 clicks*

Training round 2 (of 4):

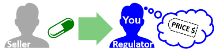

Now the Pharma Company offers a new pharmaceutical treatment which prolongs the survival of the patient by **nine** months (compared to no treatment), increasing the life expectancy by an additional **four** months compared to the current standard therapy. The treatment does not increase the quality of life compared to the standard treatment.

This work is licensed under a Creative Commons Attribution 4.0 International License. Survey created with Qualtrics.

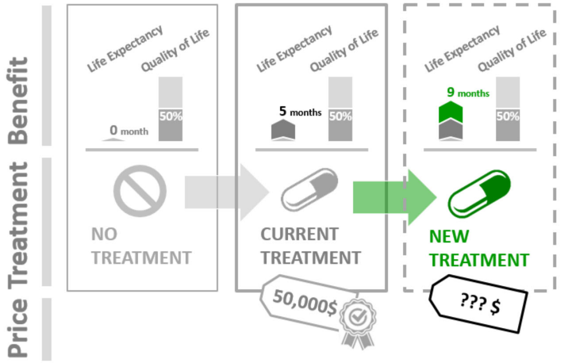

You expect the offer from the pharma company shortly. Before you enter into a negotiation, you should decide on the **absolute maximum price, which you would still consider reasonable and fair for the new product:**

Your maximum price of ___,000 $ leads to the following state of the society:			
in thousand Dollars	**New Benefit**	**Compared to Current State**	**New Asset (Benefit + Initial Asset)**
**Patient**	45	+20	45
**2 Payers**			
**2 Investors**			
**Seller**	120	0	240
**Regulator**	120	0	240

[illegible]

Please select your maximum price by moving the red slider below.
You will see the related consequences in the table above.
If the values do not change, please click the red slider again.

Your previous decision:
6 months: ${q://QID503/ChoiceNumericEntryValue/1},000 $

These page timer metrics will not be displayed to the recipient.
First Click: *0 seconds*
Last Click: *0 seconds*
Page Submit: *0 seconds*
Click Count: *0 clicks*

Training round 3 (of 4):

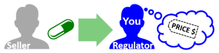

Now the Pharma Company offers a new pharmaceutical treatment which prolongs the survival of the patient by **thirteen** months (compared to no treatment), increasing the life expectancy by an additional **eight** months compared to the current standard treatment. The treatment does not increase the quality of life compared to the standard treatment.

This work is licensed under a Creative Commons Attribution 4.0 International License. Survey created with Qualtrics.

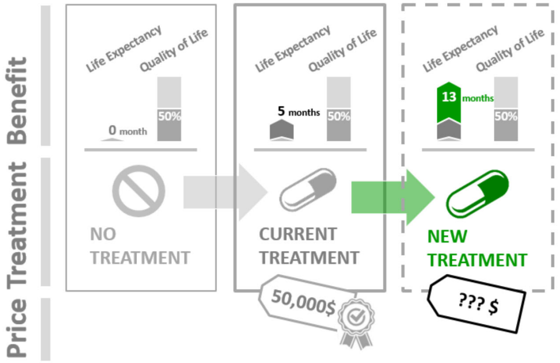

You expect the offer from the pharma company shortly. Before you enter into a negotiation, you should decide on the **absolute maximum price, which you would still consider reasonable and fair for the new product:**

Your maximum price of ___,000 $ leads to the following state of the society:			
in thousand Dollars	**New Benefit**	**Compared to Current State**	**New Asset (Benefit + Initial Asset)**
**Patient**	65	+40	65
**2 Payers**			
**2 Investors**			
**Seller**	120	0	240
**Regulator**	120	0	240

[illegible]

Please select your maximum price by moving the red slider below.
You will see the related consequences in the table above.
If the values do not change, please click the red slider again.

Your previous decisions:
6 months: ${q://QID503/ChoiceNumericEntryValue/1},000 $
9 months: ${q://QID178/ChoiceNumericEntryValue/1},000 $

These page timer metrics will not be displayed to the recipient.
First Click: *0 seconds*
Last Click: *0 seconds*
Page Submit: *0 seconds*
Click Count: *0 clicks*

Training round 4 (of 4):

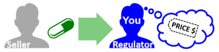

Now the Pharma Company offers a new pharmaceutical treatment which prolongs the survival of the patient by **fourteen** months (compared to no treatment), increasing the life expectancy by an additional **nine** months compared to the current standard treatment. The treatment does not increase the quality of life compared to the standard treatment.

This work is licensed under a Creative Commons Attribution 4.0 International License. Survey created with Qualtrics.

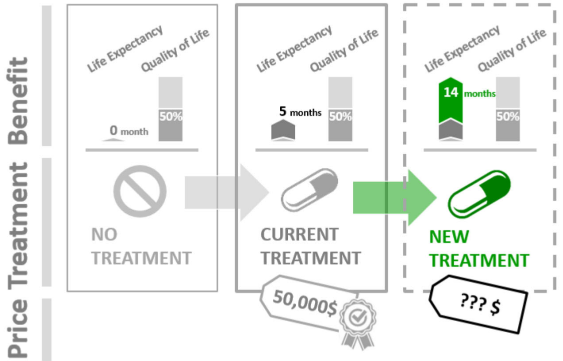

You expect the offer from the pharma company shortly. Before you enter into a negotiation, you should decide on the **absolute maximum price, which you would still consider reasonable and fair for the new product:**

Your maximum price of ___,000 $ leads to the following state of the society:			
in thousand Dollars	New Benefit	Compared to Current State	New Asset (Benefit + Initial Asset)
**Patient**	70	+45	70
**2 Payers**			
**2 Investors**			
**Seller**	120	0	240
**Regulator**	120	0	240

Please select your maximum price by moving the red slider below.
You will see the related consequences in the table above.
If the values do not change, please click the red slider again.

Your previous decisions:
6 months: ${q://QID503/ChoiceNumericEntryValue/1},000 $
9 months: ${q://QID178/ChoiceNumericEntryValue/1},000 $
13 months: ${q://QID182/ChoiceNumericEntryValue/1},000 $

These page timer metrics will not be displayed to the recipient.
First Click: *0 seconds*
Last Click: *0 seconds*
Page Submit: *0 seconds*
Click Count: *0 clicks*

You have successfully completed the training. Thank you very much!

These page timer metrics will not be displayed to the recipient.
First Click: *0 seconds*
Last Click: *0 seconds*
Page Submit: *0 seconds*
Click Count: *0 clicks*

Regulator - 1 - Intro & Training

This work is licensed under a Creative Commons Attribution 4.0 International License. Survey created with Qualtrics.

You are the responsible **Regulator** ("Health Minister") in this country. In the following, **you will receive offers from a pharma company** for the reimbursement of new pharmaceuticals. You will see their expected benefit, based on clinical studies.

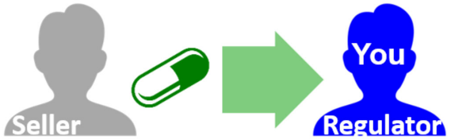

Before you enter into a negotiation, you should decide on the **absolute maximum price, which you would still consider reasonable and fair for the new product**. Above this "walk-away price" you would never allow the new pharmaceutical to be reimbursed by the public health insurance.

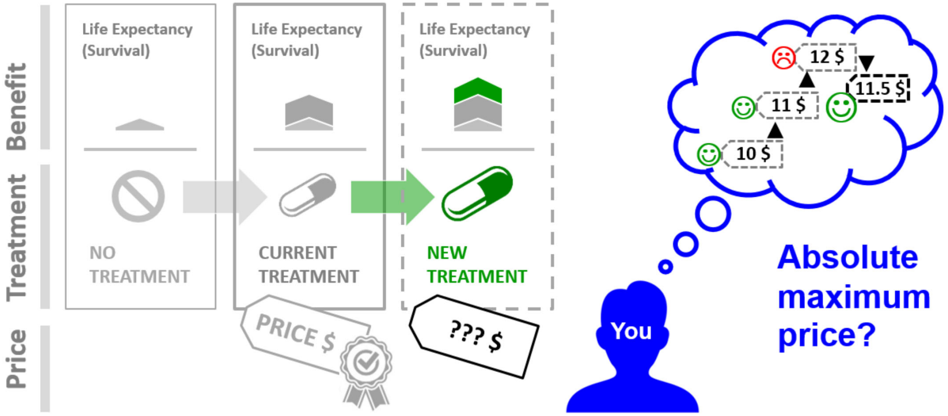

This part of the experiment focuses only on your private valuation of new health technologies. You do not have to reflect on any negotiation tactics or strategies. There is no "correct" answer; your decision should be based solely on your personal preferences.
Be aware that you will not be able to change your decisions after submission.

All prices are expressed in fictive "Dollar" ($) and trade at the end of the experiment at a currency rate of **1 $ = 1 US$**.

These page timer metrics will not be displayed to the recipient.
First Click: *0 seconds*
Last Click: *0 seconds*
Page Submit: *0 seconds*
Click Count: *0 clicks*

Control question 3: Let us assume that you are a real coffee lover (if not, you can replace coffee with soda or else). Meaning that you always benefit more from two cups than from one, more from a large cup than from a small one (see picture below). Let us further assume that 2 $ represents the maximum price you still consider reasonable and fair for a **small** cup of 12 oz.

a) Assuming all other things equal and that you have the money available and can afford it: Which price would represent your absolute maximum willingness to pay for a **medium** cup of 16 oz.?

- A price higher than 2 $
- A price of 2 $
- A price lower than 2 $

b) Would you buy a large cup of 20 oz. for 2 $?

- Yes
- No

This work is licensed under a Creative Commons Attribution 4.0 International License. Survey created with Qualtrics.

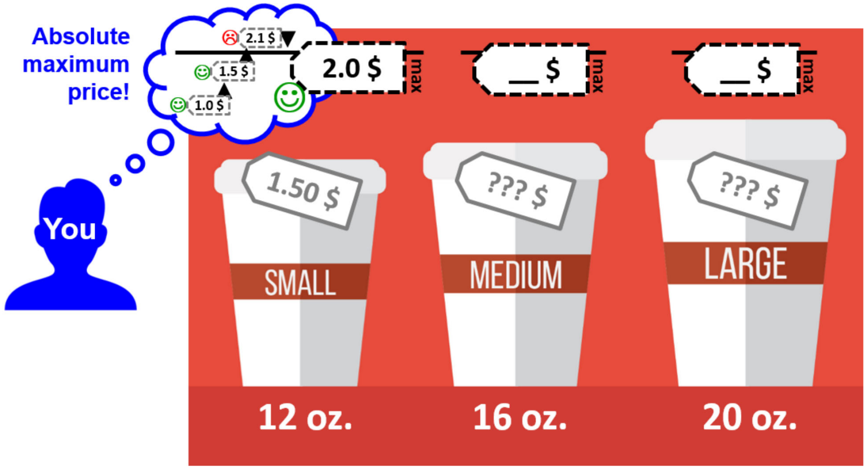

[Coffee cup adjusted from wikiHow: How to Order Coffee. wikiHow Inc.; 2019. The image is licensed under a Creative Commons Attribution-NonCommercial-ShareAlike 3.0 Unported License.]

These page timer metrics will not be displayed to the recipient.
First Click: *0 seconds*
Last Click: *0 seconds*
Page Submit: *0 seconds*
Click Count: *0 clicks*

Your decision will have real consequences on others. After this experiment is closed, you will be randomly paired with another MTurk worker who played the opposite role. The rounds will be implemented, depending on your own and your counterpart's decisions. Whenever an agreement would be possible, following payoffs will be made:

Patient: Benefit converted to US$ will be donated to the Leukemia & Lymphoma Society (LLS) which provides financial support for patients with blood cancer (https://www.lls.org/support/financial-support)
Payers: Positive benefit converted to US$ /10 will be paid to two other MTurk workers (randomly selected)
Investors: Positive benefit converted to US$ /10 will be paid to two other MTurk workers (randomly selected)
Regulator or **Seller**: Benefit converted to US$ will be paid to you and your negotiation partner. The fix salary for you both is guaranteed, regardless whether an agreement was possible (as long as the experiment was completed).

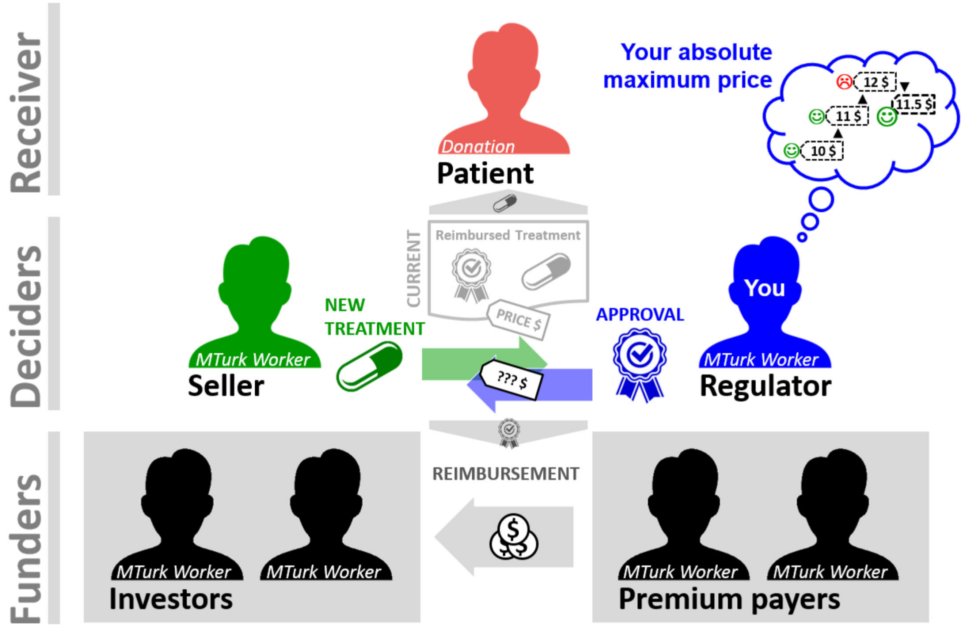

These page timer metrics will not be displayed to the recipient.
First Click: *0 seconds*
Last Click: *0 seconds*
Page Submit: *0 seconds*
Click Count: *0 clicks*

Initial position:

Patients suffer from a deadly, incurable blood cancer. With no treatment, they have a remaining life expectancy below one month. There is one pharmaceutical treatment available, which increases the patient's life expectancy (*survival*) by **five months** at an unchanged quality of life (*QoL*). The QoL is an experience-based, self-reported indicator for the patient's physical functioning, bodily pain, as well as mental, emotional and social functioning etc. It is measured at a scale from 0 to 100%. The lower the score the more disabled the patient. The QoL of the patient under current standard treatment is **50%**.

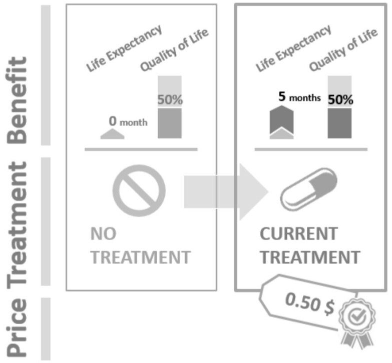

At full QoL (100%) the patient could realize a monthly income of 0.1 $. Due to the lower QoL the patient's work ability (productivity) is reduced proportionally. In consequence the potential income he/she can generate equals 0.1 $ * 50% = 0.05 $ per month. This translates into a total economic benefit for the patient under current standard therapy of 0.1 $ * **50%** * **5** months = 0.25 $.

 This work is licensed under a Creative Commons Attribution 4.0 International License. Survey created with Qualtrics.

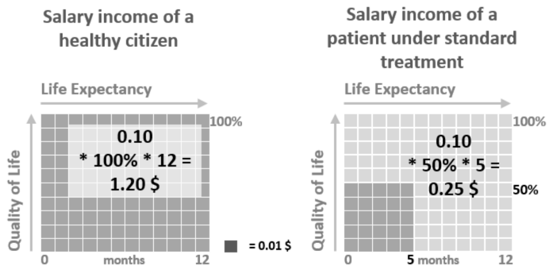

With exception of the patient, all citizens have an initial asset of 1.2 $ (equals to a yearly income at full work ability). In addition to this, the Health minister (you) as well as the representative from the pharma company expect both a fix salary of 1.2 $. The two payers will receive the same income of 1.2 $ but will have to share the health care costs, deducted from their yearly income. The two investors have no fix income but will share the revenue generated with the reimbursed pharmaceuticals.

The current standard treatment costs **0.5 $** per therapy and patient, paid by the health insurance.
In consequence each of the two payers earns currently 0.95 $ (= 1.2 – 0.5 / 2) and each of the two investors 0.25 $ (= 0.5 / 2).

Control question 4: If the patient's survival increases from zero to 4 months at the same quality of life (50%), how much will he/she gain "economically" in cash?

- ◯ Nothing, the benefit of survival cannot be expressed in monetary terms.
- ◯ Nothing, the patient cannot work at a reduced quality of life.
- ◯ 0.20 $
- ◯ 0.25 $
- ◯ 0.40 $

These page timer metrics will not be displayed to the recipient.
First Click: *0 seconds*
Last Click: *0 seconds*
Page Submit: *0 seconds*
Click Count: *0 clicks*

So much for the introduction and theory. Thank you for your patience so far!

Let us start with a short training (4 decisions), before we move to the actual experiment.

Training round 1 (of 4):

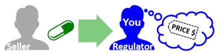

The Pharma Company offers a new pharmaceutical treatment which prolongs the survival of the patient by **six** months (compared to no treatment), increasing the life expectancy by **one** additional month compared to the current standard therapy. The treatment does not increase the quality of life compared to the standard treatment.

This work is licensed under a Creative Commons Attribution 4.0 International License. Survey created with Qualtrics.

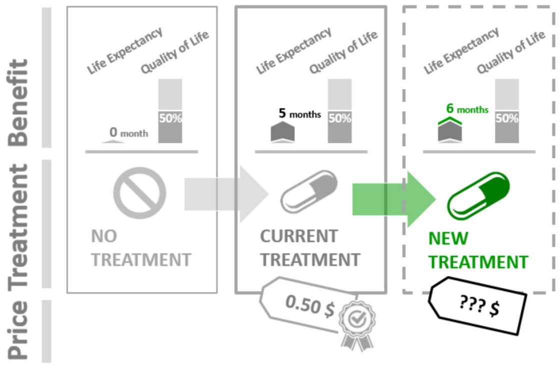

You expect the offer from the pharma company shortly. Before you enter into a negotiation, you should decide on the **absolute maximum price, which you would still consider reasonable and fair for the new product:**

Your maximum price of ___ $ leads to the following state of the society:			
in Dollars	**New Benefit**	**Compared to Current State**	**New Asset (Benefit + Initial Asset)**
**Patient**	0.3	+0.05	0.3
**2 Payers**			
**2 Investors**			
**Seller**	1.2	0	2.4
**Regulator**	1.2	0	2.4

[illegible]

Please select your maximum price by moving the red slider below.
You will see the related consequences in the table above.
If the values do not change, please click the red slider again.

These page timer metrics will not be displayed to the recipient.
First Click: *0 seconds*
Last Click: *0 seconds*
Page Submit: *0 seconds*
Click Count: *0 clicks*

Training round 2 (of 4):

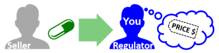

Now the Pharma Company offers a new pharmaceutical treatment which prolongs the survival of the patient by **nine** months (compared to no treatment), increasing the life expectancy by an additional **four** months compared to the current standard therapy. The treatment does not increase the quality of life compared to the standard treatment.

This work is licensed under a Creative Commons Attribution 4.0 International License. Survey created with Qualtrics.

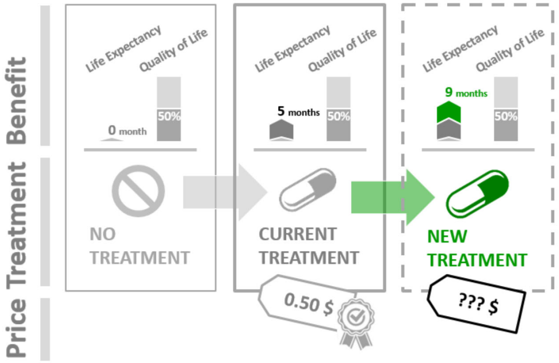

You expect the offer from the pharma company shortly. Before you enter into a negotiation, you should decide on the **absolute maximum price, which you would still consider reasonable and fair for the new product:**

Your maximum price of ___ $ leads to the following state of the society:			
in Dollars	**New Benefit**	**Compared to Current State**	**New Asset (Benefit + Initial Asset)**
**Patient**	0.45	+0.2	0.45
**2 Payers**			
**2 Investors**			
**Seller**	1.2	0	2.4
**Regulator**	1.2	0	2.4

[illegible]

Please select <u>your maximum price</u> by moving the red slider below.
You will see the related consequences in the table above.
If the values do not change, please click the red slider again.

Your previous decision:
6 months: ${q://QID432/ChoiceNumericEntryValue/1} $

These page timer metrics will not be displayed to the recipient.
First Click: *0 seconds*
Last Click: *0 seconds*
Page Submit: *0 seconds*
Click Count: *0 clicks*

Training round 3 (of 4):

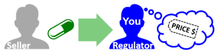

Now the Pharma Company offers a new pharmaceutical treatment which prolongs the survival of the patient by **thirteen** months (compared to no treatment), increasing the life expectancy by an additional **eight** months compared to the current standard treatment. The treatment does not increase the quality of life compared to the standard treatment.

This work is licensed under a Creative Commons Attribution 4.0 International License. Survey created with Qualtrics.

Social Preferences in Reimbursement Negotiations, Additional file 4 Wettstein D., Boes S. 2021

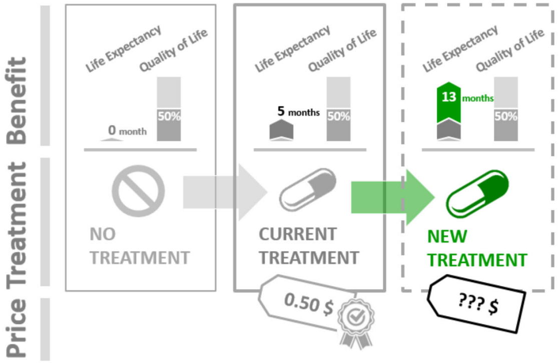

You expect the offer from the pharma company shortly. Before you enter into a negotiation, you should decide on the **absolute maximum price, which you would still consider reasonable and fair for the new product:**

Your maximum price of ___ $ leads to the following state of the society:			
in Dollars	**New Benefit**	**Compared to Current State**	**New Asset (Benefit + Initial Asset)**
**Patient**	0.65	+0.4	0.65
**2 Payers**			
**2 Investors**			
**Seller**	1.2	0	2.4
**Regulator**	1.2	0	2.4

[illegible]

Please select <u>your maximum price</u> by moving the red slider below.
You will see the related consequences in the table above.
If the values do not change, please click the red slider again.

Your previous decisions:
6 months: ${q://QID432/ChoiceNumericEntryValue/1} $
9 months: ${q://QID436/ChoiceNumericEntryValue/1} $

These page timer metrics will not be displayed to the recipient.
First Click: *0 seconds*
Last Click: *0 seconds*
Page Submit: *0 seconds*
Click Count: *0 clicks*

Training round 4 (of 4):

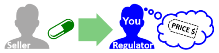

Now the Pharma Company offers a new pharmaceutical treatment which prolongs the survival of the patient by **fourteen** months (compared to no treatment), increasing the life expectancy by an additional **nine** months compared to the current standard treatment. The treatment does not increase the quality of life compared to the standard treatment.

This work is licensed under a Creative Commons Attribution 4.0 International License. Survey created with Qualtrics.

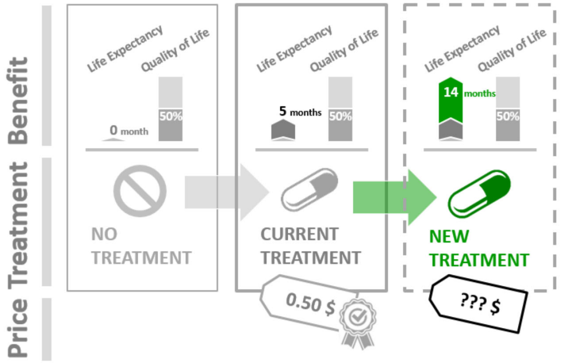

You expect the offer from the pharma company shortly. Before you enter into a negotiation, you should decide on the **absolute maximum price, which you would still consider reasonable and fair for the new product:**

Your maximum price of ___ $ leads to the following state of the society:			
in Dollars	**New Benefit**	**Compared to Current State**	**New Asset (Benefit + Initial Asset)**
**Patient**	0.7	+0.45	0.7
**2 Payers**			
**2 Investors**			
**Seller**	1.2	0	2.4
**Regulator**	1.2	0	2.4

[illegible]

Please select your maximum price by moving the red slider below.
You will see the related consequences in the table above.
If the values do not change, please click the red slider again.

Your previous decisions:
6 months: ${q://QID432/ChoiceNumericEntryValue/1} $
9 months: ${q://QID436/ChoiceNumericEntryValue/1} $
13 months: ${q://QID441/ChoiceNumericEntryValue/1} $

These page timer metrics will not be displayed to the recipient.
First Click: *0 seconds*
Last Click: *0 seconds*
Page Submit: *0 seconds*
Click Count: *0 clicks*

You have successfully completed the training. Thank you very much!

These page timer metrics will not be displayed to the recipient.
First Click: *0 seconds*
Last Click: *0 seconds*
Page Submit: *0 seconds*
Click Count: *0 clicks*

Seller - 100'000 - Intro & Training

This work is licensed under a Creative Commons Attribution 4.0 International License. Survey created with Qualtrics.

You represent an international pharmaceutical company as **Seller**. Your company focuses on research and development (R&D) of new pharmaceuticals. In the following, you will be responsible to **sell new products to the Health Minister** of this country. You will see their expected benefit, based on clinical studies.

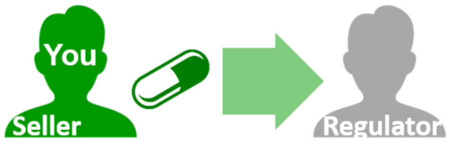

Before you enter into a negotiation, you should decide on the **absolute minimum price, which you would still consider reasonable and fair for your new product**. Bellow this "walk-away price" you would never agree to sell the new pharmaceutical in this country.

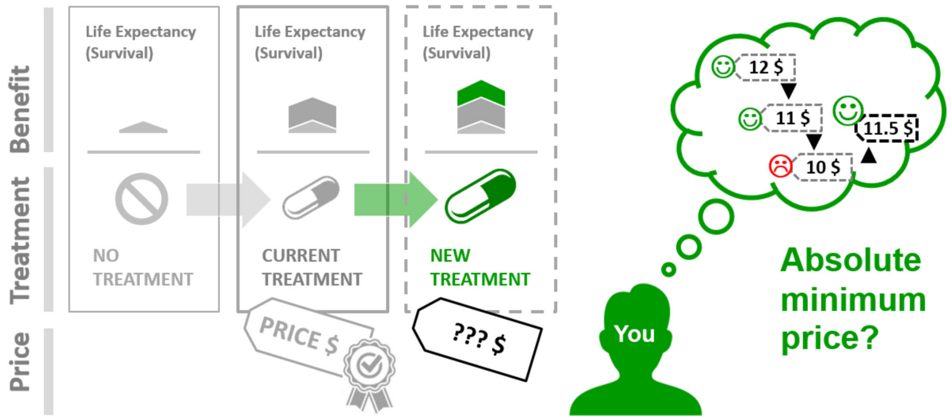

This part of the experiment focuses only on your private valuation of new health technologies. You do not have to reflect on any negotiation tactics or strategies. There is no "correct" answer; your decision should be based solely on your personal preferences.
Be aware that you will not be able to change your decisions after submission.

All prices are expressed in fictive "Dollar" ($) and trade at the end of the experiment at a currency rate of **100,000 $ = 1 US$.**

These page timer metrics will not be displayed to the recipient.
First Click: *0 seconds*
Last Click: *0 seconds*
Page Submit: *0 seconds*
Click Count: *0 clicks*

Control question 3: Let us pretend that you are a coffee shop owner. We further assume that your customers are real coffee lovers. Meaning that they always benefit more from two cups than from one, more from a large cup than from a small one (see picture below). Let us finally assume that 1 $ represents the minimum price you still consider reasonable and fair for a **small** cup of 12 oz.

a) Assuming that increasing your coffee output requires additional effort (investment) from you: Which price would represent your absolute minimum willingness to sell a **medium** cup of 16 oz.?

- A price higher than 1 $
- A price of 1 $
- A price lower than 1 $

b) Would you sell the large cup of 20 oz. for 1 $?

- Yes
- No

This work is licensed under a Creative Commons Attribution 4.0 International License. Survey created with Qualtrics.

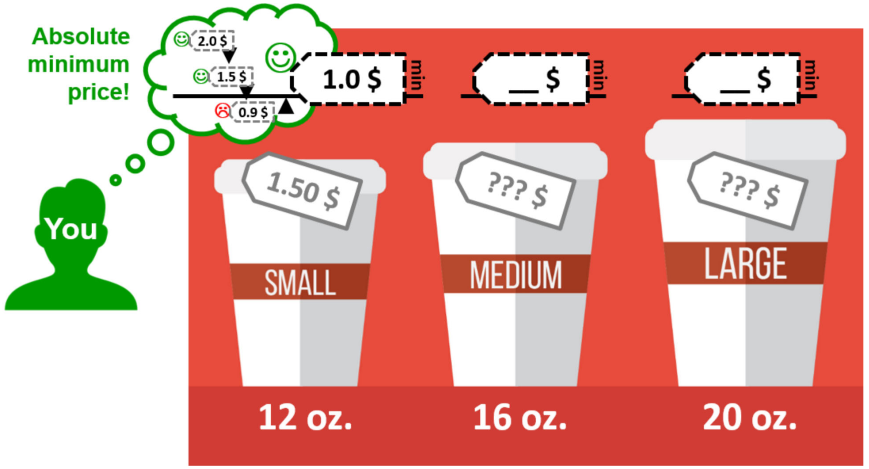

[Coffee cup adjusted from wikiHow: How to Order Coffee. wikiHow Inc.; 2019. The image is licensed under a Creative Commons Attribution-NonCommercial-ShareAlike 3.0 Unported License.]

These page timer metrics will not be displayed to the recipient.
First Click: *0 seconds*
Last Click: *0 seconds*
Page Submit: *0 seconds*
Click Count: *0 clicks*

Your decision will have real consequences on others. After this experiment is closed, you will be randomly paired with another MTurk worker who played the opposite role. The rounds will be implemented, depending on your own and your counterpart's decisions. Whenever an agreement would be possible, following payoffs will be made:

Patient: Benefit converted to US$ will be donated to the Leukemia & Lymphoma Society (LLS) which provides financial support for patients with blood cancer (https://www.lls.org/support/financial-support)
Payers: Positive benefit converted to US$ /10 will be paid to two other MTurk workers (randomly selected)
Investors: Positive benefit converted to US$ /10 will be paid to two other MTurk workers (randomly selected)
Regulator or **Seller**: Benefit converted to US$ will be paid to you and your negotiation partner. The fix salary for you both is guaranteed, regardless whether an agreement was possible (as long as the experiment was completed).

Social Preferences in Reimbursement Negotiations, Additional file 4 Wettstein D., Boes S. 2021

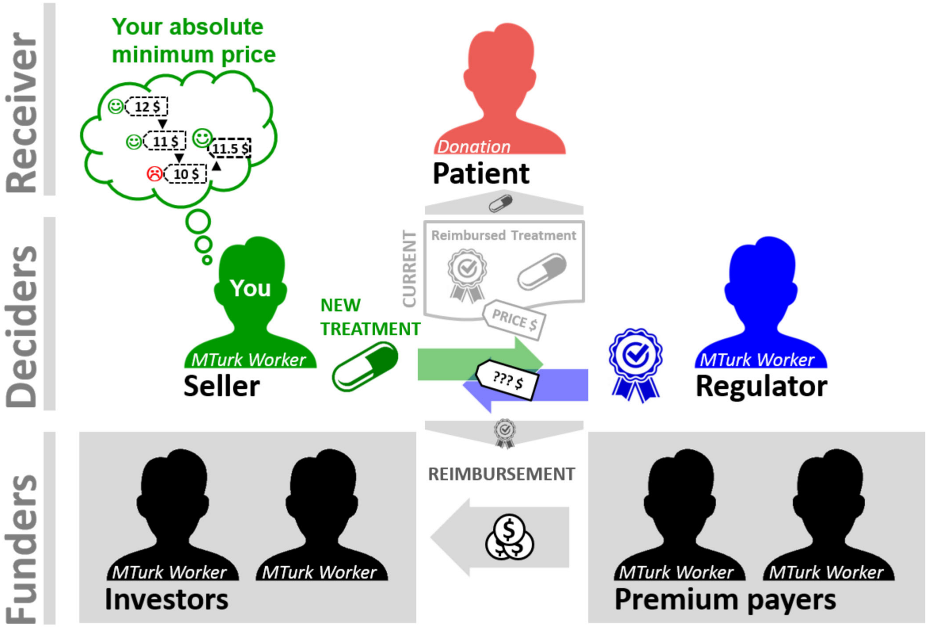

These page timer metrics will not be displayed to the recipient.
First Click: *0 seconds*
Last Click: *0 seconds*
Page Submit: *0 seconds*
Click Count: *0 clicks*

Initial position:

Patients suffer from a deadly, incurable blood cancer. With no treatment, they have a remaining life expectancy below one month. There is one pharmaceutical treatment available, which increases the patient's life expectancy (*survival*) by **five months** at an unchanged quality of life (*QoL*). The QoL is an experience-based, self-reported indicator for the patient's physical functioning, bodily pain, as well as mental, emotional and social functioning etc. It is measured at a scale from 0 to 100%. The lower the score the more disabled the patient. The QoL of the patient under current standard treatment is **50%**.

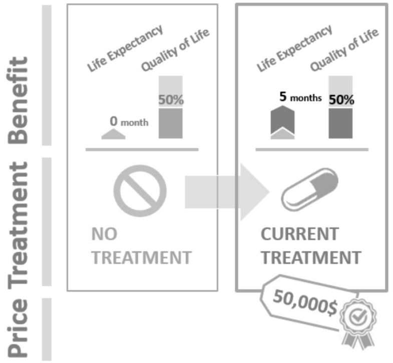

At full QoL (100%) the patient could realize a monthly income of 10,000 $. Due to the lower QoL the patient's work ability (productivity) is reduced proportionally. In consequence the potential income he/she can generate equals 10,000 $ * 50% = 5,000 $ per month. This translates into a total economic benefit for the patient under current standard treatment of 10,000 $ * **50%** * **5** months = 25,000 $.

 This work is licensed under a Creative Commons Attribution 4.0 International License. Survey created with Qualtrics.

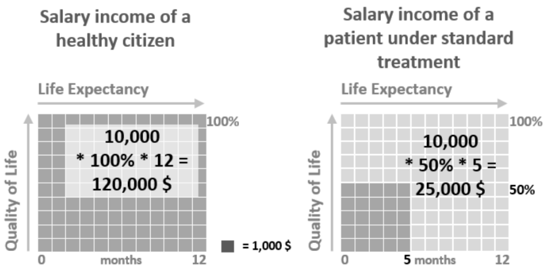

With exception of the patient, all citizens have an initial asset of 120,000 $ (equals to a yearly income at full work ability). In addition to this, the Health minister as well as the representative from the pharma company (you) expect both a fix salary of 120,000 $. The two payers will receive the same income of 120,000 $ but will have to share the health care costs, deducted from their yearly income. The two investors have no fix income but will share the revenue generated with the reimbursed pharmaceuticals.

The current standard treatment costs **50,000 $** per therapy and patient, paid by the health insurance.
In consequence each of the two payers earns currently 95,000 $ (= 120,000 – 50,000 / 2) and each of the two investors 25,000 $ (= 50,000 / 2).

Control question 4: If the patient's survival increases from zero to 4 months at the same quality of life (50%), how much will he/she gain "economically" in cash?

- Nothing, the benefit of survival cannot be expressed in monetary terms.
- Nothing, the patient cannot work at a reduced quality of life.
- 20,000 $
- 25,000 $
- 40,000 $

These page timer metrics will not be displayed to the recipient.
First Click: *0 seconds*
Last Click: *0 seconds*
Page Submit: *0 seconds*
Click Count: *0 clicks*

So much for the introduction and theory. Thank you for your patience so far!

Let us start with a short training (4 decisions), before we move to the actual experiment.

Training round 1 (of 4):

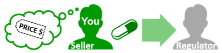

Your company developed a new treatment which prolongs the survival of the patient by **six** months (compared to no treatment), increasing the life expectancy by **one** additional month compared to the current standard treatment. The treatment does not increase the quality of life compared to the standard treatment.

This work is licensed under a Creative Commons Attribution 4.0 International License. Survey created with Qualtrics.

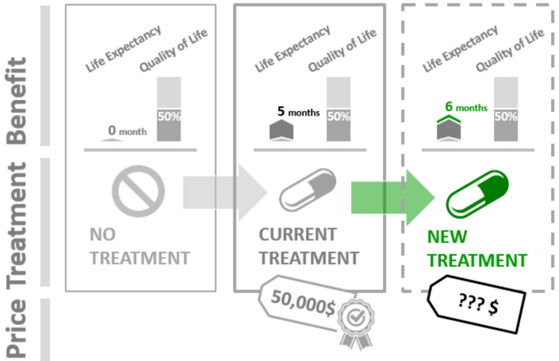

You have to prepare an offer for the Health minister shortly. Before you enter into a negotiation, you should decide on the **absolute minimum price, which you would still consider reasonable and fair for the new product:**

Your minimum price of ___,000 $ leads to the following state of the society:			
in thousand Dollars	**New Benefit**	**Compared to Current State**	**New Asset (Benefit + Initial Asset)**
**Patient**	30	+5	30
**2 Payers**			
**2 Investors**			
**Seller**	120	0	240
**Regulator**	120	0	240

[illegible]

Please select your minimum price by moving the red slider below.
You will see the related consequences in the table above.
If the values do not change, please click the red slider again.

These page timer metrics will not be displayed to the recipient.
First Click: *0 seconds*
Last Click: *0 seconds*
Page Submit: *0 seconds*
Click Count: *0 clicks*

Training round 2 (of 4):

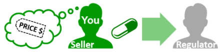

Now your company developed a new treatment which prolongs the survival of the patient by **nine** months (compared to no treatment), increasing the life expectancy by an additional **four** months compared to the current standard treatment. The treatment does not increase the quality of life compared to the standard treatment.

This work is licensed under a Creative Commons Attribution 4.0 International License. Survey created with Qualtrics.

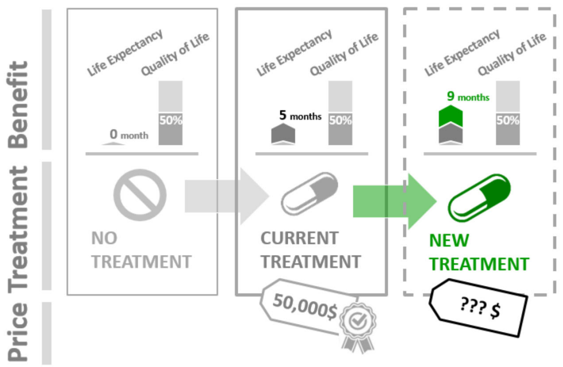

You have to prepare an offer for the Health minister shortly. Before you enter into a negotiation, you should decide on the **absolute minimum price, which you would still consider reasonable and fair for the new product:**

Your minimum price of ___,000 $ leads to the following state of the society:			
in thousand Dollars	**New Benefit**	**Compared to Current State**	**New Asset (Benefit + Initial Asset)**
**Patient**	45	+20	45
**2 Payers**			
**2 Investors**			
**Seller**	120	0	240
**Regulator**	120	0	240

[illegible]

Please select your minimum price by moving the red slider below.
You will see the related consequences in the table above.
If the values do not change, please click the red slider again.

Your previous decision:
6 months: ${q://QID202/ChoiceNumericEntryValue/1},000 $

These page timer metrics will not be displayed to the recipient.
First Click: *0 seconds*
Last Click: *0 seconds*
Page Submit: *0 seconds*
Click Count: *0 clicks*

Training round 3 (of 4):

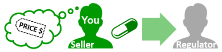

Now your company developed a new treatment which prolongs the survival of the patient by **thirteen** months (compared to no treatment), increasing the life expectancy by an additional **eight** months compared to the current standard treatment. The treatment does not increase the quality of life compared to the standard treatment.

This work is licensed under a Creative Commons Attribution 4.0 International License. Survey created with Qualtrics.

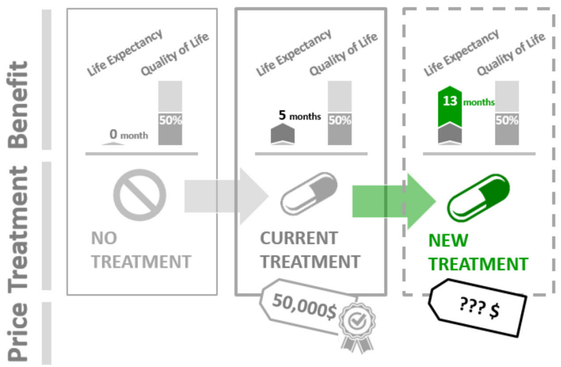

You have to prepare an offer for the Health minister shortly. Before you enter into a negotiation, you should decide on the **absolute minimum price, which you would still consider reasonable and fair for the new product:**

Your maximum price of ___,000 $ leads to the following state of the society:			
in thousand Dollars	**New Benefit**	**Compared to Current State**	**New Asset (Benefit + Initial Asset)**
**Patient**	65	+40	65
**2 Payers**			
**2 Investors**			
**Seller**	120	0	240
**Regulator**	120	0	240

[illegible]

Please select your minimum price by moving the red slider below.
You will see the related consequences in the table above.
If the values do not change, please click the red slider again.

Your previous decision:
6 months: ${q://QID202/ChoiceNumericEntryValue/1},000 $
9 months: ${q://QID206/ChoiceNumericEntryValue/1},000 $

These page timer metrics will not be displayed to the recipient.
First Click: *0 seconds*
Last Click: *0 seconds*
Page Submit: *0 seconds*
Click Count: *0 clicks*

Training round 4 (of 4):

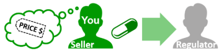

Now your company developed a new treatment which prolongs the survival of the patient by **fourteen** months (compared to no treatment), increasing the life expectancy by an additional **nine** months compared to the current standard treatment. The treatment does not increase the quality of life compared to the standard treatment.

This work is licensed under a Creative Commons Attribution 4.0 International License. Survey created with Qualtrics.

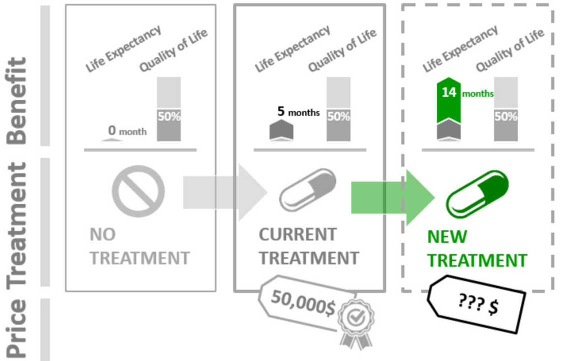

You have to prepare an offer for the Health minister shortly. Before you enter into a negotiation, you should decide on the **absolute minimum price, which you would still consider reasonable and fair for the new product:**

Your minimum price of ___,000 $ leads to the following state of the society:			
in thousand Dollars	New Benefit	Compared to Current State	New Asset (Benefit + Initial Asset)
**Patient**	70	+45	70
**2 Payers**			
**2 Investors**			
**Seller**	120	0	240
**Regulator**	120	0	240

Please select <u>your minimum price</u> by moving the red slider below.
You will see the related consequences in the table above.
If the values do not change, please click the red slider again.

Your previous decision:
6 months: ${q://QID202/ChoiceNumericEntryValue/1},000 $
9 months: ${q://QID206/ChoiceNumericEntryValue/1},000 $
13 months: ${q://QID210/ChoiceNumericEntryValue/1},000 $

These page timer metrics will not be displayed to the recipient.
First Click: *0 seconds*
Last Click: *0 seconds*
Page Submit: *0 seconds*
Click Count: *0 clicks*

You have successfully completed the training. Thank you very much!

These page timer metrics will not be displayed to the recipient.
First Click: *0 seconds*
Last Click: *0 seconds*
Page Submit: *0 seconds*
Click Count: *0 clicks*

Seller - 1 - Intro & Training

This work is licensed under a Creative Commons Attribution 4.0 International License. Survey created with Qualtrics.

You represent an international pharmaceutical company as **Seller**. Your company focuses on research and development (R&D) of new pharmaceuticals. In the following, you will be responsible to **sell new products to the Health Minister** of this country. You will see their expected benefit, based on clinical studies.

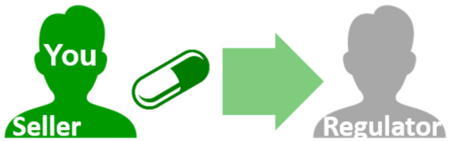

Before you enter into a negotiation, you should decide on the **absolute minimum price, which you would still consider reasonable and fair for your new product**. Bellow this "walk-away price" you would never agree to sell the new pharmaceutical in this country.

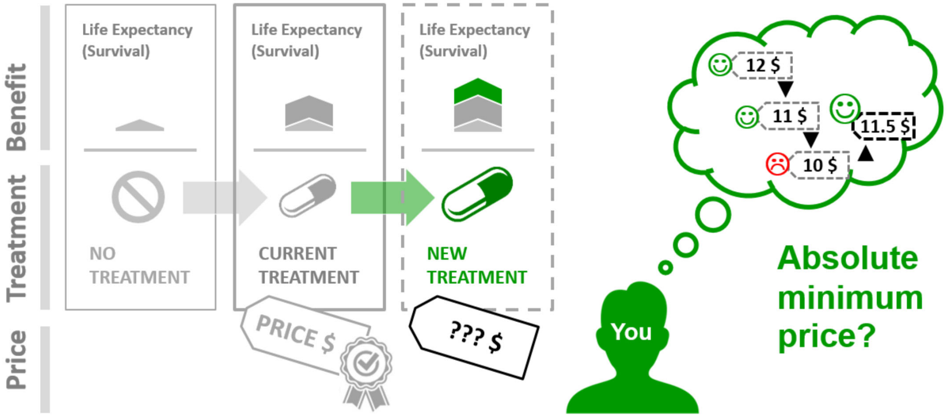

This part of the experiment focuses only on your private valuation of new health technologies. You do not have to reflect on any negotiation tactics or strategies. There is no "correct" answer; your decision should be based solely on your personal preferences.
Be aware that you will not be able to change your decisions after submission.

All prices are expressed in fictive "Dollar" ($) and trade at the end of the experiment at a currency rate of **1 $ = 1 US$.**

These page timer metrics will not be displayed to the recipient.
First Click: *0 seconds*
Last Click: *0 seconds*
Page Submit: *0 seconds*
Click Count: *0 clicks*

<u>Control question 3</u>: Let us pretend that you are a coffee shop owner. We further assume that your customers are real coffee lovers. Meaning that they always benefit more from two cups than from one, more from a large cup than from a small one (see picture below). Let us finally assume that 1 $ represents the minimum price you still consider reasonable and fair for a **small** cup of 12 oz.

a) Assuming that increasing your coffee output requires additional effort (investment) from you: Which price would represent your absolute minimum willingness to sell a **medium** cup of 16 oz.?

- A price higher than 1 $
- A price of 1 $
- A price lower than 1 $

b) Would you sell the large cup of 20 oz. for 1 $?

- Yes
- No

This work is licensed under a Creative Commons Attribution 4.0 International License. Survey created with Qualtrics.

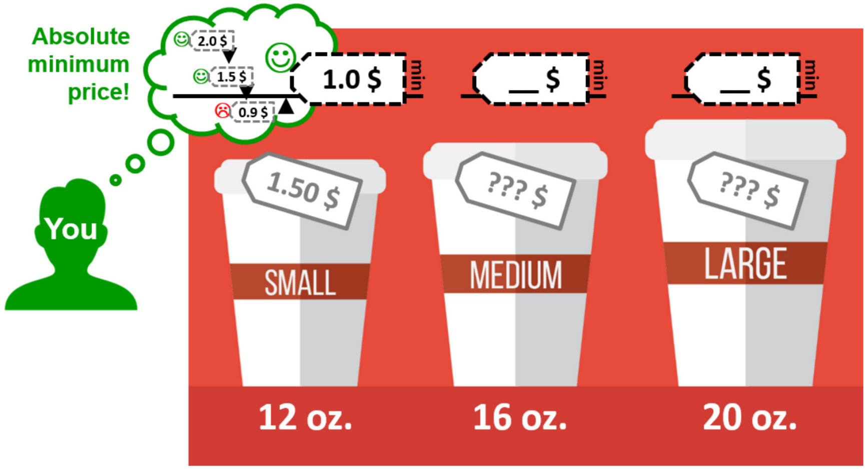

[Coffee cup adjusted from wikiHow: How to Order Coffee. wikiHow Inc.; 2019. The image is licensed under a Creative Commons Attribution-NonCommercial-ShareAlike 3.0 Unported License.]

These page timer metrics will not be displayed to the recipient.
First Click: *0 seconds*
Last Click: *0 seconds*
Page Submit: *0 seconds*
Click Count: *0 clicks*

Your decision will have real consequences on others. After this experiment is closed, you will be randomly paired with another MTurk worker who played the opposite role. The rounds will be implemented, depending on your own and your counterpart's decisions. Whenever an agreement would be possible, following payoffs will be made:

Patient: Benefit converted to US$ will be donated to the Leukemia & Lymphoma Society (LLS) which provides financial support for patients with blood cancer (https://www.lls.org/support/financial-support)
Payers: Positive benefit converted to US$ /10 will be paid to two other MTurk workers (randomly selected)
Investors: Positive benefit converted to US$ /10 will be paid to two other MTurk workers (randomly selected)
Regulator or **Seller**: Benefit converted to US$ will be paid to you and your negotiation partner. The fix salary for you both is guaranteed, regardless whether an agreement was possible (as long as the experiment was completed).

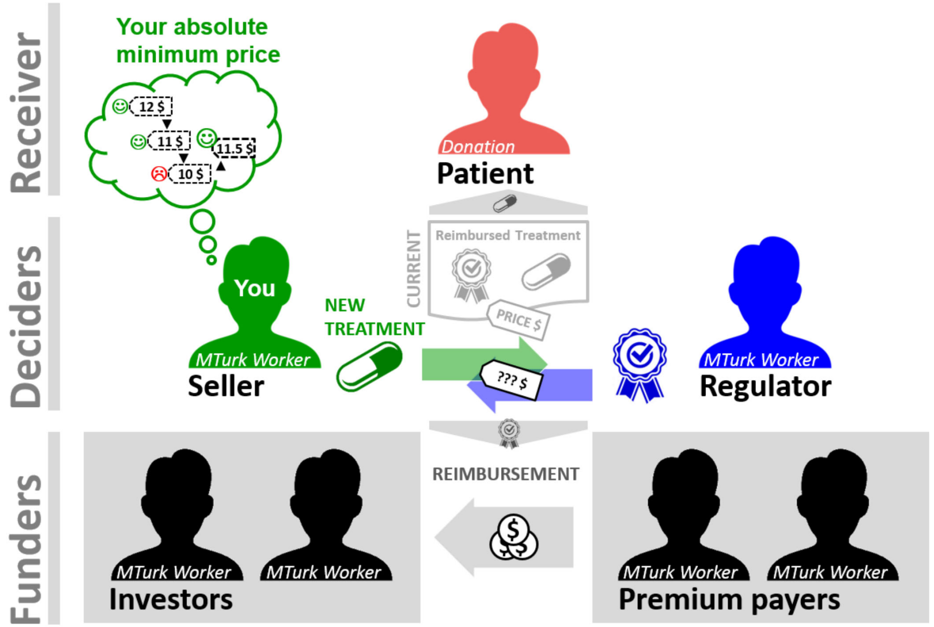

These page timer metrics will not be displayed to the recipient.
First Click: *0 seconds*
Last Click: *0 seconds*
Page Submit: *0 seconds*
Click Count: *0 clicks*

Initial position:

Patients suffer from a deadly, incurable blood cancer. With no treatment, they have a remaining life expectancy below one month. There is one pharmaceutical treatment available, which increases the patient's life expectancy (*survival*) by **five months** at an unchanged quality of life (*QoL*). The QoL is an experience-based, self-reported indicator for the patient's physical functioning, bodily pain, as well as mental, emotional and social functioning etc. It is measured at a scale from 0 to 100%. The lower the score the more disabled the patient. The QoL of the patient under current standard treatment is **50%**.

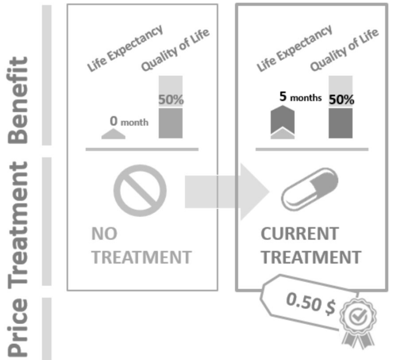

At full QoL (100%) the patient could realize a monthly income of 0.1 $. Due to the lower QoL the patient's work ability (productivity) is reduced proportionally. In consequence the potential income he/she can generate equals 0.1 $ * 50% = 0.05 $ per month. This translates into a total economic benefit for the patient under current standard treatment of 0.1 $ * **50%** * **5** months = 0.25 $.

 This work is licensed under a Creative Commons Attribution 4.0 International License. Survey created with Qualtrics.

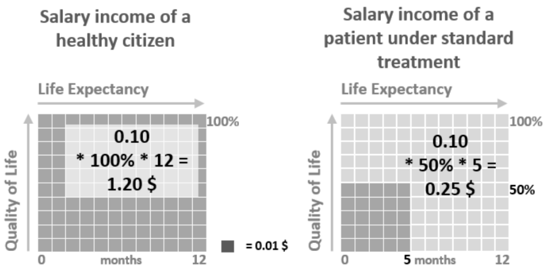

With exception of the patient, all citizens have an initial asset of 1.2 $ (equals to a yearly income at full work ability). In addition to this, the Health minister as well as the representative from the pharma company (you) expect both a fix salary of 1.2 $. The two payers will receive the same income of 1.2 $ but will have to share the health care costs, deducted from their yearly income. The two investors have no fix income but will share the revenue generated with the reimbursed pharmaceuticals.

The current standard treatment costs **0.5 $** per therapy and patient, paid by the health insurance.
In consequence each of the two payers earns currently 0.95 $ (= 1.2 – 0.5 / 2) and each of the two investors 0.25 $ (= 0.5 / 2).

Control question 4: If the patient's survival increases from zero to 4 months at the same quality of life (50%), how much will he/she gain "economically" in cash?

- Nothing, the benefit of survival cannot be expressed in monetary terms.
- Nothing, the patient cannot work at a reduced quality of life.
- 0.20 $
- 0.25 $
- 0.40 $

These page timer metrics will not be displayed to the recipient.
First Click: *0 seconds*
Last Click: *0 seconds*
Page Submit: *0 seconds*
Click Count: *0 clicks*

So much for the introduction and theory. Thank you for your patience so far!

Let us start with a short training (4 decisions), before we move to the actual experiment.

Training round 1 (of 4):

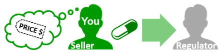

Your company developed a new treatment which prolongs the survival of the patient by **six** months (compared to no treatment), increasing the life expectancy by **one** additional month compared to the current standard treatment. The treatment does not increase the quality of life compared to the standard treatment.

This work is licensed under a Creative Commons Attribution 4.0 International License. Survey created with Qualtrics.

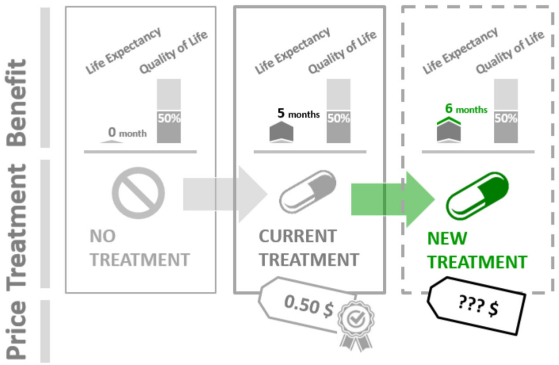

You have to prepare an offer for the Health minister shortly. Before you enter into a negotiation, you should decide on the **absolute minimum price, which you would still consider reasonable and fair for the new product:**

Your minimum price of ___ $
leads to the following state of the society:

in Dollars	**New Benefit**	**Compared to Current State**	**New Asset (Benefit + Initial Asset)**
**Patient**	0.3	+0.05	0.3
**2 Payers**			
**2 Investors**			
**Seller**	1.2	0	2.4
**Regulator**	1.2	0	2.4

[illegible]

Please select your minimum price by moving the red slider below.
You will see the related consequences in the table above.
If the values do not change, please click the red slider again.

These page timer metrics will not be displayed to the recipient.
First Click: *0 seconds*
Last Click: *0 seconds*
Page Submit: *0 seconds*
Click Count: *0 clicks*

Training round 2 (of 4):

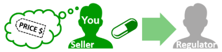

Now your company developed a new treatment which prolongs the survival of the patient by **nine** months (compared to no treatment), increasing the life expectancy by an additional **four** months compared to the current standard treatment. The treatment does not increase the quality of life compared to the standard treatment.

This work is licensed under a Creative Commons Attribution 4.0 International License. Survey created with Qualtrics.

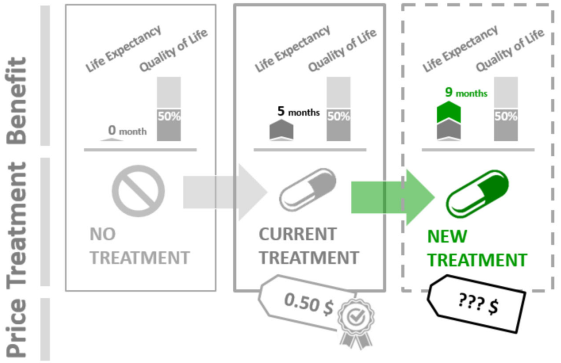

You have to prepare an offer for the Health minister shortly. Before you enter into a negotiation, you should decide on the **absolute minimum price, which you would still consider reasonable and fair for the new product:**

Your minimum price of ___ $ leads to the following state of the society:			
in Dollars	**New Benefit**	**Compared to Current State**	**New Asset (Benefit + Initial Asset)**
**Patient**	0.45	+0.2	0.45
**2 Payers**			
**2 Investors**			
**Seller**	1.2	0	2.4
**Regulator**	1.2	0	2.4

[illegible]

Please select your minimum price by moving the red slider below.
You will see the related consequences in the table above.
If the values do not change, please click the red slider again.

Your previous decision:

6 months: ${q://QID461/ChoiceNumericEntryValue/1} $

These page timer metrics will not be displayed to the recipient.
First Click: *0 seconds*
Last Click: *0 seconds*
Page Submit: *0 seconds*
Click Count: *0 clicks*

Training round 3 (of 4):

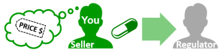

Now your company developed a new treatment which prolongs the survival of the patient by **thirteen** months (compared to no treatment), increasing the life expectancy by an additional **eight** months compared to the current standard treatment. The treatment does not increase the quality of life compared to the standard treatment.

This work is licensed under a Creative Commons Attribution 4.0 International License. Survey created with Qualtrics.

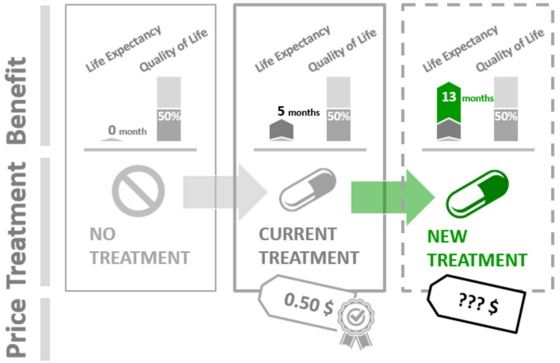

You have to prepare an offer for the Health minister shortly. Before you enter into a negotiation, you should decide on the **absolute minimum price, which you would still consider reasonable and fair for the new product:**

Your maximum price of ___ $ leads to the following state of the society:			
in Dollars	**New Benefit**	**Compared to Current State**	**New Asset (Benefit + Initial Asset)**
**Patient**	0.65	+0.4	0.65
**2 Payers**			
**2 Investors**			
**Seller**	1.2	0	2.4
**Regulator**	1.2	0	2.4

[illegible]

Please select your minimum price by moving the red slider below.
You will see the related consequences in the table above.
If the values do not change, please click the red slider again.

Your previous decision:

6 months: ${q://QID461/ChoiceNumericEntryValue/1} $

9 months: ${q://QID465/ChoiceNumericEntryValue/1} $

These page timer metrics will not be displayed to the recipient.
First Click: *0 seconds*
Last Click: *0 seconds*
Page Submit: *0 seconds*
Click Count: *0 clicks*

Training round 4 (of 4):

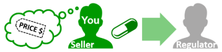

Now your company developed a new treatment which prolongs the survival of the patient by **fourteen** months (compared to no treatment), increasing the life expectancy by an additional **nine** months compared to the current standard treatment. The treatment does not increase the quality of life compared to the standard treatment.

This work is licensed under a Creative Commons Attribution 4.0 International License. Survey created with Qualtrics.

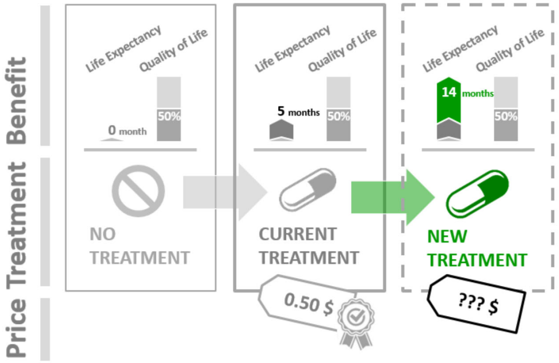

You have to prepare an offer for the Health minister shortly. Before you enter into a negotiation, you should decide on the **absolute minimum price, which you would still consider reasonable and fair for the new product:**

Your minimum price of ___ $ leads to the following state of the society:			
in Dollars	**New Benefit**	**Compared to Current State**	**New Asset (Benefit + Initial Asset)**
**Patient**	0.7	+0.45	0.7
**2 Payers**			
**2 Investors**			
**Seller**	1.2	0	2.4
**Regulator**	1.2	0	2.4

[illegible]

Please select <u>your minimum price</u> by moving the red slider below.
You will see the related consequences in the table above.
If the values do not change, please click the red slider again.

Your previous decision:
6 months: ${q://QID461/ChoiceNumericEntryValue/1} $
9 months: ${q://QID465/ChoiceNumericEntryValue/1} $
13 months: ${q://QID470/ChoiceNumericEntryValue/1} $

These page timer metrics will not be displayed to the recipient.
First Click: *0 seconds*
Last Click: *0 seconds*
Page Submit: *0 seconds*
Click Count: *0 clicks*

You have successfully completed the training. Thank you very much!

These page timer metrics will not be displayed to the recipient.
First Click: *0 seconds*
Last Click: *0 seconds*
Page Submit: *0 seconds*
Click Count: *0 clicks*

Regulator - 100'000 - Reservation Price

This work is licensed under a Creative Commons Attribution 4.0 International License. Survey created with Qualtrics.

Let us start with the experiment!

These page timer metrics will not be displayed to the recipient.
First Click: *0 seconds*
Last Click: *0 seconds*
Page Submit: *0 seconds*
Click Count: *0 clicks*

Decision situation (1 of 5):

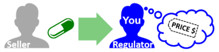

The Pharma Company offers a new pharmaceutical treatment which prolongs the survival of the patient by **eight** months (compared to no treatment), increasing the life expectancy by an additional **three** months compared to the current standard therapy. The treatment does not increase the quality of life compared to the standard treatment.

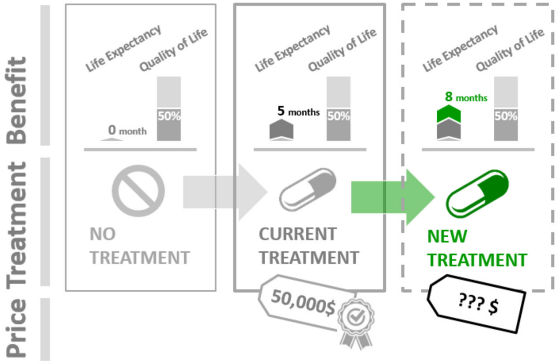

You expect the offer from the pharma company shortly. Before you enter into a negotiation, you should decide on the **absolute maximum price, which you would still consider reasonable and fair for the new product:**

Your maximum price of ___,000 $ leads to the following state of the society:			
in thousand Dollars	**New Benefit**	**Compared to Current State**	**New Asset (Benefit + Initial Asset)**
**Patient**	40	+15	40
**2 Payers**			
**2 Investors**			
**Seller**	120	0	240
**Regulator**	120	0	240

[illegible]

Please select <u>your maximum price</u> by moving the red slider below.
You will see the related consequences in the table above.
If the values do not change, please click the red slider again.

These page timer metrics will not be displayed to the recipient.
First Click: *0 seconds*
Last Click: *0 seconds*
Page Submit: *0 seconds*
Click Count: *0 clicks*

This work is licensed under a Creative Commons Attribution 4.0 International License. Survey created with Qualtrics.

Decision situation (2 of 5):

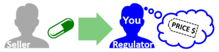

Now the Pharma Company offers a new pharmaceutical treatment which prolongs the survival of the patient by **ten** months (compared to no treatment), increasing the life expectancy by an additional **five** months compared to the current standard treatment. The treatment does not increase the quality of life compared to the standard treatment.

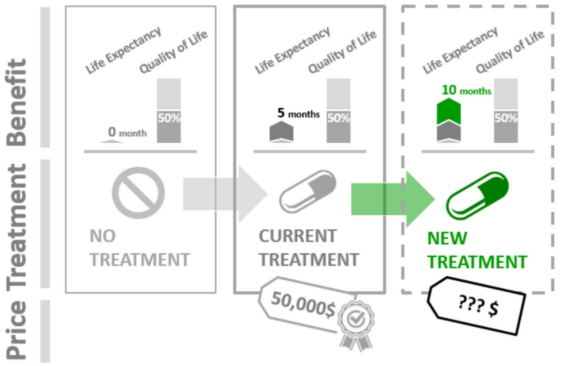

You expect the offer from the pharma company shortly. Before you enter into a negotiation, you should decide on the **absolute maximum price, which you would still consider reasonable and fair for the new product:**

Your maximum price of ___,000 $ leads to the following state of the society:			
in thousand Dollars	**New Benefit**	**Compared to Current State**	**New Asset (Benefit + Initial Asset)**
**Patient**	50	+25	50
**2 Payers**			
**2 Investors**			
**Seller**	120	0	240
**Regulator**	120	0	240

Please select <u>your maximum price</u> by moving the red slider below.
You will see the related consequences in the table above.
If the values do not change, please click the red slider again.

Your previous decision:
8 months: ${q://QID116/ChoiceNumericEntryValue/1},000 $

These page timer metrics will not be displayed to the recipient.
First Click: *0 seconds*
Last Click: *0 seconds*
Page Submit: *0 seconds*
Click Count: *0 clicks*

Decision situation (3 of 5):

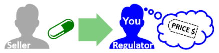

Now the Pharma Company offers a new pharmaceutical treatment which prolongs the survival of the patient by **twelve** months (compared to no treatment),

 This work is licensed under a Creative Commons Attribution 4.0 International License. Survey created with Qualtrics.

increasing the life expectancy by an additional **seven** months compared to the current standard treatment. The treatment does not increase the quality of life compared to the standard treatment.

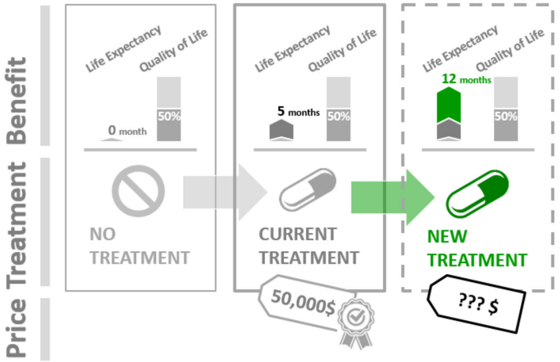

You expect the offer from the pharma company shortly. Before you enter into a negotiation, you should decide on the **absolute maximum price, which you would still consider reasonable and fair for the new product:**

Your maximum price of ___,000 $ leads to the following state of the society:			
in thousand Dollars	**New Benefit**	**Compared to Current State**	**New Asset (Benefit + Initial Asset)**
**Patient**	60	+35	60
**2 Payers**			
**2 Investors**			
**Seller**	120	0	240
**Regulator**	120	0	240

Please select <u>your maximum price</u> by moving the red slider below.
You will see the related consequences in the table above.
If the values do not change, please click the red slider again.

Your previous decisions:
8 months: ${q://QID116/ChoiceNumericEntryValue/1},000 $
10 months: ${q://QID117/ChoiceNumericEntryValue/1},000 $

These page timer metrics will not be displayed to the recipient.
First Click: *0 seconds*
Last Click: *0 seconds*
Page Submit: *0 seconds*
Click Count: *0 clicks*

Decision situation (4 of 5):

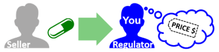

Now the Pharma Company offers a new pharmaceutical treatment which prolongs the survival of the patient by **fifteen** months (compared to no treatment), increasing the life expectancy by an additional **ten** months compared to the current standard treatment. The treatment does not increase the quality of life compared to the standard treatment.

 This work is licensed under a Creative Commons Attribution 4.0 International License. Survey created with Qualtrics.

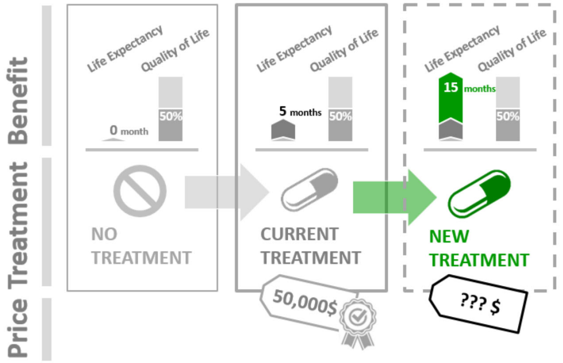

You expect the offer from the pharma company shortly. Before you enter into a negotiation, you should decide on the **absolute maximum price, which you would still consider reasonable and fair for the new product:**

Your maximum price of ___,000 $ leads to the following state of the society:			
in thousand Dollars	**New Benefit**	**Compared to Current State**	**New Asset (Benefit + Initial Asset)**
**Patient**	75	+50	75
**2 Payers**			
**2 Investors**			
**Seller**	120	0	240
**Regulator**	120	0	240

[illegible]

Please select your maximum price by moving the red slider below.
You will see the related consequences in the table above.
If the values do not change, please click the red slider again.

Your previous decisions:
8 months: ${q://QID116/ChoiceNumericEntryValue/1},000 $
10 months: ${q://QID117/ChoiceNumericEntryValue/1},000 $
12 months: ${q://QID118/ChoiceNumericEntryValue/1},000 $

These page timer metrics will not be displayed to the recipient.
First Click: *0 seconds*
Last Click: *0 seconds*
Page Submit: *0 seconds*
Click Count: *0 clicks*

Decision situation (5 of 5):

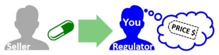

Now the Pharma Company offers a new pharmaceutical treatment which prolongs the survival of the patient by **seventeen** months (compared to no treatment), increasing the life expectancy by an additional **twelve** months compared to the current standard treatment. The treatment does not increase the quality of life compared to the standard treatment.

This work is licensed under a Creative Commons Attribution 4.0 International License. Survey created with Qualtrics.

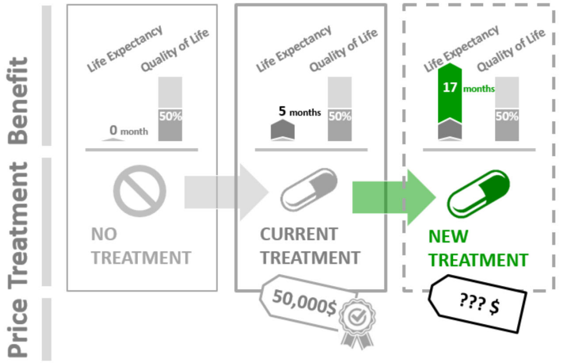

You expect the offer from the pharma company shortly. Before you enter into a negotiation, you should decide on the **absolute maximum price, which you would still consider reasonable and fair for the new product:**

Your maximum price of ___,000 $ leads to the following state of the society:			
in thousand Dollars	**New Benefit**	**Compared to Current State**	**New Asset (Benefit + Initial Asset)**
**Patient**	85	+60	85
**2 Payers**			
**2 Investors**			
**Seller**	120	0	240
**Regulator**	120	0	240

[illegible]

Please select your maximum price by moving the red slider below.
You will see the related consequences in the table above.
If the values do not change, please click the red slider again.

Your previous decisions:
8 months: ${q://QID116/ChoiceNumericEntryValue/1},000 $
10 months: ${q://QID117/ChoiceNumericEntryValue/1},000 $
12 months: ${q://QID118/ChoiceNumericEntryValue/1},000 $
15 months: ${q://QID119/ChoiceNumericEntryValue/1},000 $

These page timer metrics will not be displayed to the recipient.
First Click: *0 seconds*
Last Click: *0 seconds*
Page Submit: *0 seconds*
Click Count: *0 clicks*

Decision situation (1 of 1):

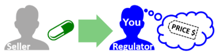

Now the Pharma Company offers a new treatment which prolongs the survival of the patient by minus **one month** (compared to no treatment), decreasing the life expectancy below **zero**. This obviously makes no sense. We just need to test, whether participants read the relevant information. If you did so, please click on the green pill below and submit without entering a price.

This work is licensed under a Creative Commons Attribution 4.0 International License. Survey created with Qualtrics.

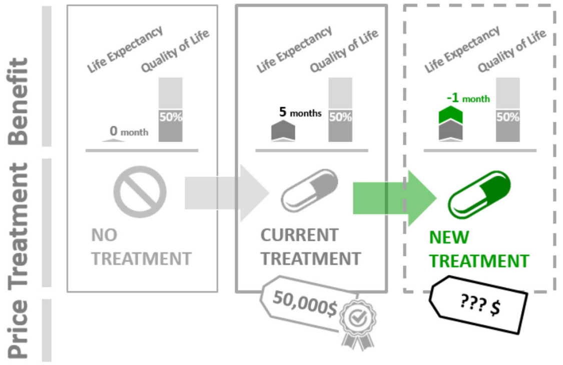

You expect the offer from the pharma company shortly. Before you enter into a negotiation, you should decide on the **absolute maximum price, which you would still consider reasonable and fair for the new product:**

Your maximum price of ___,000 $ leads to the following state of the society:			
in thousand Dollars	**New Benefit**	**Compared to Current State**	**New Asset (Benefit + Initial Asset)**
**Patient**	-5	-30	-30
**2 Payers**			
**2 Investors**			
**Seller**	120	0	240
**Regulator**	120	0	240

Please select your maximum price by moving the red slider below.
You will see the related consequences in the table above.
If the values do not change, please click the red slider again.

These page timer metrics will not be displayed to the recipient.
First Click: *0 seconds*
Last Click: *0 seconds*
Page Submit: *0 seconds*
Click Count: *0 clicks*

For the past rounds: please indicate in descending order the relevance of the stakeholders for your decision.
You can drag and drop the options below to bring them in your preferred order.

Negotiation partner

Investors

Own role

Premium payers

Patient

 This work is licensed under a Creative Commons Attribution 4.0 International License. Survey created with Qualtrics.

These page timer metrics will not be displayed to the recipient.
First Click: *0 seconds*
Last Click: *0 seconds*
Page Submit: *0 seconds*
Click Count: *0 clicks*

Transition to Price Offer

First set done! You did great – thank you!!
That was the introspective part of the experiment. Now let us switch to a more interactive mode...

Regulator - 100'000 - Price Offer

We will revisit the five new pharmaceutical products of round 1 to 5. But now you can state an actual price expectation to the seller. The seller himself will do the same. Your counterpart is another participant, randomly selected after the experiment.

If your expectation is **higher or equal** to the price offer of the seller, you will both agree. In consequence, the patient will get access to the new treatment with all its benefits (increased life expectancy, economic benefit). The investors will receive the price (revenue) and the payers will have to pay the price (cost). If no agreement is reached, the product will not be available in this country.

You and your counterpart will both receive **an additional bonus for reaching an agreement: you can keep the difference between** your successful price statement and your absolute maximum price (stated in the first part of the experiment). Hence, the lower your price statement compared to your absolute maximum price, the higher your bonus – if the offer is not below the seller's price statement. Then again, the higher your price expectation, the higher potentially the chance of reaching an agreement with the seller.

Below the decision table you will see for each round your stated maximum prices from the first part of the experiment.

Ok? Let us start!

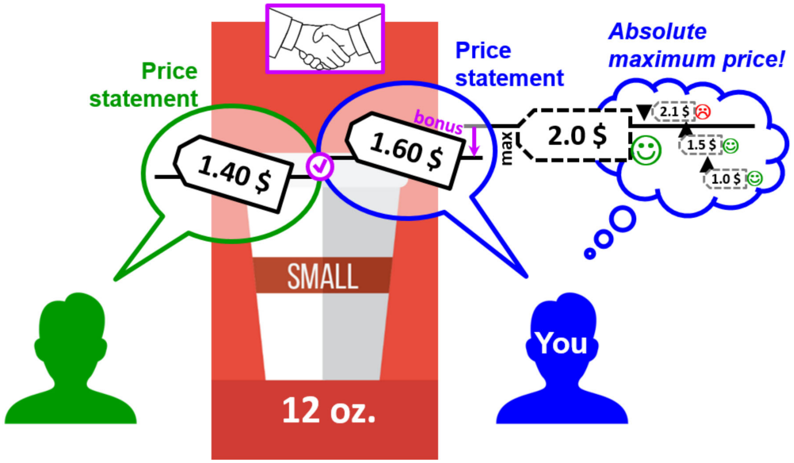

[Coffee cup adjusted from wikiHow: How to Order Coffee. wikiHow Inc.; 2019. The image is licensed under a Creative Commons Attribution-NonCommercial-ShareAlike 3.0 Unported License.]

Decision situation (1 of 5):

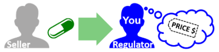

The Pharma Company offers a new pharmaceutical treatment which prolongs the survival of the patient by **eight** months (compared to no treatment), increasing the life expectancy by an additional **three** months compared to the current standard therapy. The treatment does not increase the quality of life compared to the standard treatment.

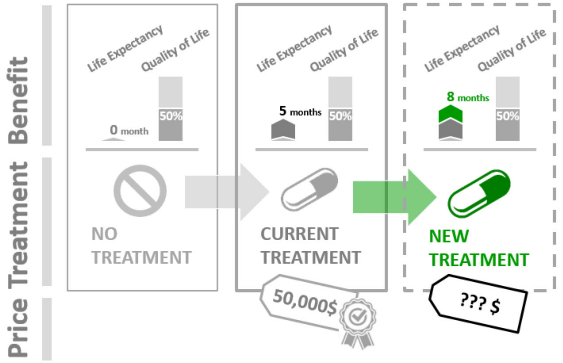

Please chose your price statement to the seller for this product (the maximum prices that you have defined before are displayed below the decision table).

Your maximum price of ___,000 $ leads to the following state of the society:			
in thousand Dollars	**New Benefit**	**Compared to Current State**	**New Asset (Benefit + Initial Asset)**
**Patient**	40	+15	40
**2 Payers**			
**2 Investors**			
**Seller**	120	0	240
**Regulator**	120	0	240

[illegible]

Please state your price by moving the red slider below. You will see the related consequences in the table above. *If the values do not change, please click the red slider again.*

Your maximum prices form the first five rounds:
8 months: ${q://QID116/ChoiceNumericEntryValue/1},000 $
10 months: ${q://QID117/ChoiceNumericEntryValue/1},000 $
12 months: ${q://QID118/ChoiceNumericEntryValue/1},000 $
15 months: ${q://QID119/ChoiceNumericEntryValue/1},000 $
17 months: ${q://QID120/ChoiceNumericEntryValue/1},000 $

These page timer metrics will not be displayed to the recipient.
First Click: *0 seconds*
Last Click: *0 seconds*
Page Submit: *0 seconds*
Click Count: *0 clicks*

Decision situation (2 of 5):

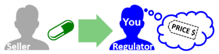

Now the Pharma Company offers a new pharmaceutical treatment which prolongs the survival of the patient by **ten** months (compared to no treatment), increasing the life expectancy by an additional **five** months compared to the current standard treatment. The treatment does not increase the quality of life compared to the standard treatment.

This work is licensed under a Creative Commons Attribution 4.0 International License. Survey created with Qualtrics.

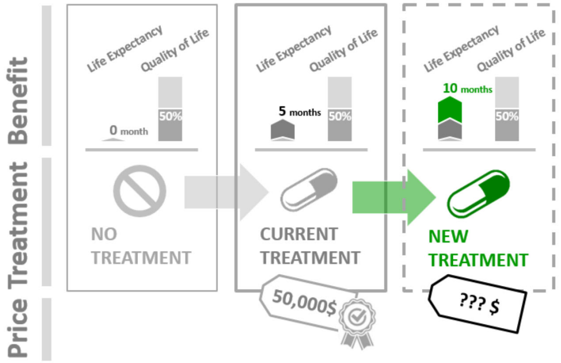

Please chose your price statement to the seller for this product (the maximum prices that you have defined before are displayed below the decision table).

Your maximum price of ___,000 $ leads to the following state of the society:			
in thousand Dollars	**New Benefit**	**Compared to Current State**	**New Asset (Benefit + Initial Asset)**
**Patient**	50	+25	50
**2 Payers**			
**2 Investors**			
**Seller**	120	0	240
**Regulator**	120	0	240

[illegible]

Please state your price by moving the red slider below. You will see the related consequences in the table above. *If the values do not change, please click the red slider again.*

Your maximum prices form the first five rounds:
8 months: ${q://QID116/ChoiceNumericEntryValue/1},000 $
10 months: ${q://QID117/ChoiceNumericEntryValue/1},000 $
12 months: ${q://QID118/ChoiceNumericEntryValue/1},000 $
15 months: ${q://QID119/ChoiceNumericEntryValue/1},000 $
17 months: ${q://QID120/ChoiceNumericEntryValue/1},000 $

These page timer metrics will not be displayed to the recipient.
First Click: *0 seconds*
Last Click: *0 seconds*
Page Submit: *0 seconds*
Click Count: *0 clicks*

Decision situation (3 of 5):

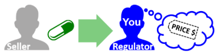

Now the Pharma Company offers a new pharmaceutical treatment which prolongs the survival of the patient by **twelve** months (compared to no treatment), increasing the life expectancy by an additional **seven** months compared to the current standard treatment. The treatment does not increase the quality of life compared to the standard treatment.

This work is licensed under a Creative Commons Attribution 4.0 International License. Survey created with Qualtrics.

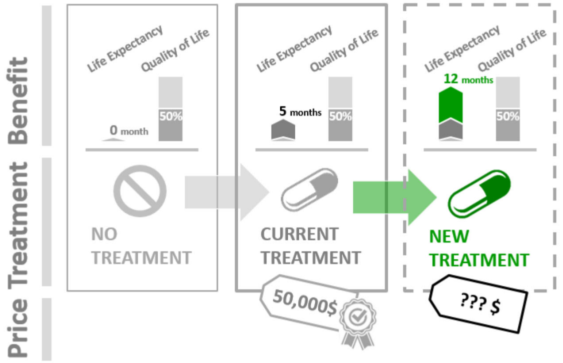

Please chose your price statement to the seller for this product (the maximum prices that you have defined before are displayed below the decision table).

Your maximum price of ___,000 $ leads to the following state of the society:			
in thousand Dollars	**New Benefit**	**Compared to Current State**	**New Asset (Benefit + Initial Asset)**
**Patient**	60	+35	60
**2 Payers**			
**2 Investors**			
**Seller**	120	0	240
**Regulator**	120	0	240

[illegible]

Please <u>state your price</u> by moving the red slider below. You will see the related consequences in the table above.
If the values do not change, please click the red slider again.

Your maximum prices form the first five rounds:
8 months: ${q://QID116/ChoiceNumericEntryValue/1},000 $
10 months: ${q://QID117/ChoiceNumericEntryValue/1},000 $
12 months: ${q://QID118/ChoiceNumericEntryValue/1},000 $
15 months: ${q://QID119/ChoiceNumericEntryValue/1},000 $
17 months: ${q://QID120/ChoiceNumericEntryValue/1},000 $

These page timer metrics will not be displayed to the recipient.
First Click: *0 seconds*
Last Click: *0 seconds*
Page Submit: *0 seconds*
Click Count: *0 clicks*

Decision situation (4 of 5):

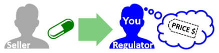

Now the Pharma Company offers a new pharmaceutical treatment which prolongs the survival of the patient by **fifteen** months (compared to no treatment), increasing the life expectancy by an additional **ten** months compared to the current standard treatment. The treatment does not increase the quality of life compared to the standard treatment.

This work is licensed under a Creative Commons Attribution 4.0 International License. Survey created with Qualtrics.

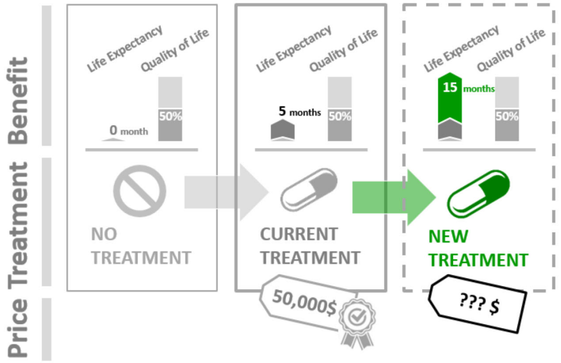

Please chose your price statement to the seller for this product (the maximum prices that you have defined before are displayed below the decision table).

Your maximum price of ___,000 $ leads to the following state of the society:			
in thousand Dollars	**New Benefit**	**Compared to Current State**	**New Asset (Benefit + Initial Asset)**
**Patient**	75	+50	75
**2 Payers**			
**2 Investors**			
**Seller**	120	0	240
**Regulator**	120	0	240

[illegible]

Please <u>state your price</u> by moving the red slider below. You will see the related consequences in the table above.
If the values do not change, please click the red slider again.

Your maximum prices form the first five rounds:
8 months: ${q://QID116/ChoiceNumericEntryValue/1},000 $
10 months: ${q://QID117/ChoiceNumericEntryValue/1},000 $
12 months: ${q://QID118/ChoiceNumericEntryValue/1},000 $
15 months: ${q://QID119/ChoiceNumericEntryValue/1},000 $
17 months: ${q://QID120/ChoiceNumericEntryValue/1},000 $

These page timer metrics will not be displayed to the recipient.
First Click: *0 seconds*
Last Click: *0 seconds*
Page Submit: *0 seconds*
Click Count: *0 clicks*

Decision situation (5 of 5):

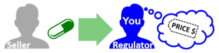

Now the Pharma Company offers a new pharmaceutical treatment which prolongs the survival of the patient by **seventeen** months (compared to no treatment), increasing the life expectancy by an additional **twelve** months compared to the current standard treatment. The treatment does not increase the quality of life compared to the standard treatment.

This work is licensed under a Creative Commons Attribution 4.0 International License. Survey created with Qualtrics.

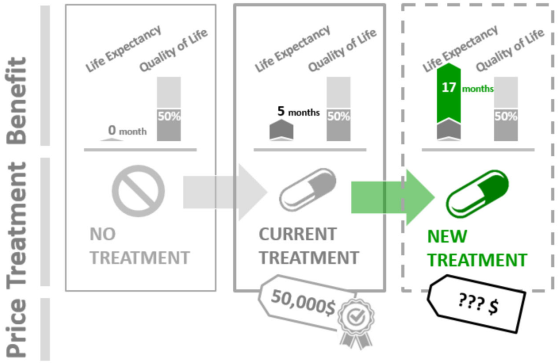

Please chose your price statement to the seller for this product (the maximum prices that you have defined before are displayed below the decision table).

Your maximum price of ___,000 $ leads to the following state of the society:			
in thousand Dollars	**New Benefit**	**Compared to Current State**	**New Asset (Benefit + Initial Asset)**
**Patient**	85	+60	85
**2 Payers**			
**2 Investors**			
**Seller**	120	0	240
**Regulator**	120	0	240

[illegible]

Please state your price by moving the red slider below. You will see the related consequences in the table above.
If the values do not change, please click the red slider again.

Your maximum prices form the first five rounds:
8 months: ${q://QID116/ChoiceNumericEntryValue/1},000 $
10 months: ${q://QID117/ChoiceNumericEntryValue/1},000 $
12 months: ${q://QID118/ChoiceNumericEntryValue/1},000 $
15 months: ${q://QID119/ChoiceNumericEntryValue/1},000 $
17 months: ${q://QID120/ChoiceNumericEntryValue/1},000 $

These page timer metrics will not be displayed to the recipient.
First Click: *0 seconds*
Last Click: *0 seconds*
Page Submit: *0 seconds*
Click Count: *0 clicks*

For the past five rounds: please indicate in descending order the relevance of the stakeholders for your decision.
You can drag and drop the options below to bring them in your preferred order.

Negotiation partner
Premium payers
Own role
Patient

This work is licensed under a Creative Commons Attribution 4.0 International License. Survey created with Qualtrics.

Investors

These page timer metrics will not be displayed to the recipient.
First Click: *0 seconds*
Last Click: *0 seconds*
Page Submit: *0 seconds*
Click Count: *0 clicks*

Closing ALL

Great! We are almost done. May we please ask you a few final questions?

For each of the following statements, please indicate the likelihood that you would engage in the described activity or behavior if you were to find yourself in that situation. Provide a rating from "extremely unlikely" to "extremely likely", using the slider:

	Extremely unlikely	Moderately unlikely	Somewhat unlikely	Not sure	Somewhat likely	Moderately likely	Extremely likely
Investing 10% of your annual income in a moderate growth mutual fund.	○	○	○	○	○	○	○
Betting a day's income at a high-stake poker game.	○	○	○	○	○	○	○
Passing off somebody else's work as your own.	○	○	○	○	○	○	○
Having an affair with a married man/woman.	○	○	○	○	○	○	○
Investing 5% of your annual income in a very speculative stock.	○	○	○	○	○	○	○
Investing 10% of your annual income in a new business venture.	○	○	○	○	○	○	○
Leaving your young children alone at home while running an errand.	○	○	○	○	○	○	○
Not returning a wallet you found that contains $200.	○	○	○	○	○	○	○

These page timer metrics will not be displayed to the recipient.
First Click: *0 seconds*
Last Click: *0 seconds*
Page Submit: *0 seconds*
Click Count: *0 clicks*

Please answer with yes or no:
Remember: All your responses in this experiment are anonymous.

	yes	no	I don't want to answer
Have you ever been affected by a chronic and/or severe disease (but not now)?	○	○	○
Do you personally suffer from a chronic and/or severe disease (currently under treatment)?	○	○	○
Are people close to you (family or friends) suffering from a severe and/or chronic disease?	○	○	○
Have you ever discussed with a person close to you (family or friend) who suffers from a chronic disease his or her condition?	○	○	○
Have you or people close to you (family or friends) ever needed pharmaceutical treatment for a longer period?	○	○	○

These page timer metrics will not be displayed to the recipient.
First Click: *0 seconds*
Last Click: *0 seconds*
Page Submit: *0 seconds*
Click Count: *0 clicks*

What is your educational background:

This work is licensed under a Creative Commons Attribution 4.0 International License. Survey created with Qualtrics.

What is your current employment status?

Have you ever been employed by a... (multiple answers possible)

- ☐ Health care provider (hospital, clinic, elderly home, or similar) ?
- ☐ Public authority, responsible for health care (e.g. FDA) ?
- ☐ Pharmaceutical company or related?
- ☐ Medical device company?
- ☐ Pharmacy, drugstore or wholesale company focusing on health care?
- ☐ Health insurance or health plan?
- ☐ Patient organization?
- ☐ Research organization focusing on health care?
- ☐ Any other organization involved in or focusing on health care?
- ☐ No (none of above)

These page timer metrics will not be displayed to the recipient.
First Click: *0 seconds*
Last Click: *0 seconds*
Page Submit: *0 seconds*
Click Count: *0 clicks*

Do you have any feedback to the researchers regarding this survey?

Expect your bonus payments within the next 10 days.

These page timer metrics will not be displayed to the recipient.
First Click: *0 seconds*
Last Click: *0 seconds*
Page Submit: *0 seconds*
Click Count: *0 clicks*

Regulator - 1 - Reservation Price

Let us start with the experiment!

These page timer metrics will not be displayed to the recipient.
First Click: *0 seconds*
Last Click: *0 seconds*
Page Submit: *0 seconds*
Click Count: *0 clicks*

Decision situation (1 of 5):

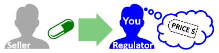

The Pharma Company offers a new pharmaceutical treatment which prolongs the survival of the patient by **eight** months (compared to no treatment), increasing the life expectancy by an additional **three** months compared to the current standard therapy. The treatment does not increase the quality of life compared to the standard treatment.

This work is licensed under a Creative Commons Attribution 4.0 International License. Survey created with Qualtrics.

Social Preferences in Reimbursement Negotiations, Additional file 4 Wettstein D., Boes S. 2021

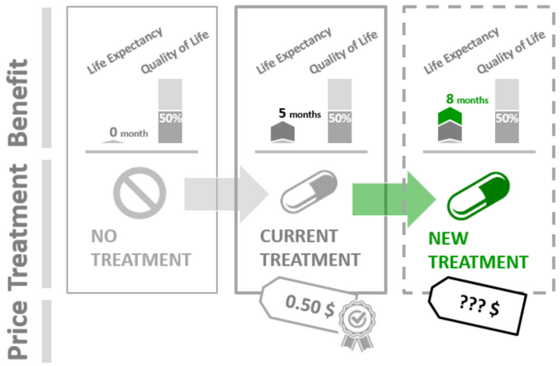

You expect the offer from the pharma company shortly. Before you enter into a negotiation, you should decide on the **absolute maximum price, which you would still consider reasonable and fair for the new product:**

Your maximum price of ___ $ leads to the following state of the society:			
in Dollars	**New Benefit**	**Compared to Current State**	**New Asset (Benefit + Initial Asset)**
**Patient**	0.4	+0.15	0.4
**2 Payers**			
**2 Investors**			
**Seller**	1.2	0	2.4
**Regulator**	1.2	0	2.4

[illegible]

Please select your maximum price by moving the red slider below.
You will see the related consequences in the table above.
If the values do not change, please click the red slider again.

These page timer metrics will not be displayed to the recipient.
First Click: *0 seconds*
Last Click: *0 seconds*
Page Submit: *0 seconds*
Click Count: *0 clicks*

Decision situation (2 of 5):

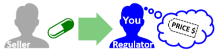

Now the Pharma Company offers a new pharmaceutical treatment which prolongs the survival of the patient by **ten** months (compared to no treatment), increasing the life expectancy by an additional **five** months compared to the current standard treatment. The treatment does not increase the quality of life compared to the standard treatment.

This work is licensed under a Creative Commons Attribution 4.0 International License. Survey created with Qualtrics.

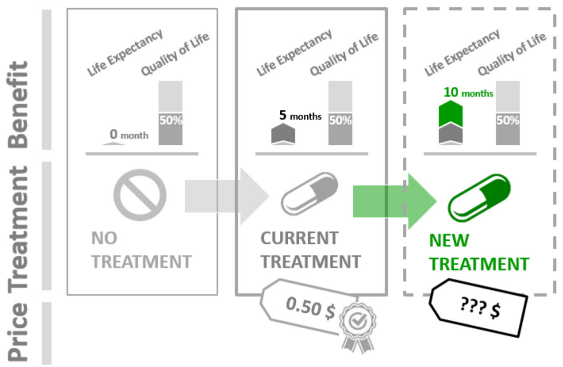

You expect the offer from the pharma company shortly. Before you enter into a negotiation, you should decide on the **absolute maximum price, which you would still consider reasonable and fair for the new product:**

Your maximum price of ___ $ leads to the following state of the society:			
in Dollars	**New Benefit**	**Compared to Current State**	**New Asset (Benefit + Initial Asset)**
**Patient**	0.5	+0.25	0.5
**2 Payers**			
**2 Investors**			
**Seller**	1.2	0	2.4
**Regulator**	1.2	0	2.4

Please select your maximum price by moving the red slider below.
You will see the related consequences in the table above.
If the values do not change, please click the red slider again.

Your previous decision:
8 months: ${q://QID313/ChoiceNumericEntryValue/1} $

These page timer metrics will not be displayed to the recipient.
First Click: *0 seconds*
Last Click: *0 seconds*
Page Submit: *0 seconds*
Click Count: *0 clicks*

Decision situation (3 of 5):

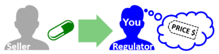

Now the Pharma Company offers a new pharmaceutical treatment which prolongs the survival of the patient by **twelve** months (compared to no treatment), increasing the life expectancy by an additional **seven** months compared to the current standard treatment. The treatment does not increase the quality of life compared to the standard treatment.

This work is licensed under a Creative Commons Attribution 4.0 International License. Survey created with Qualtrics.

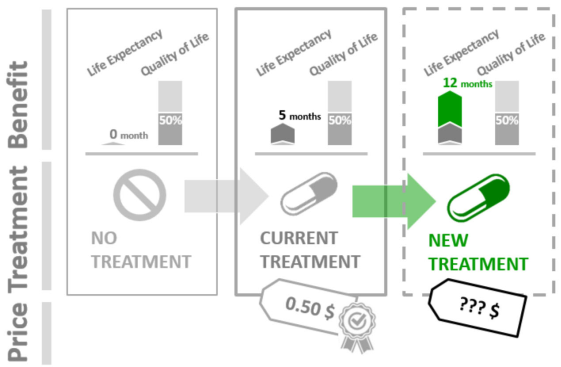

You expect the offer from the pharma company shortly. Before you enter into a negotiation, you should decide on the **absolute maximum price, which you would still consider reasonable and fair for the new product:**

Your maximum price of ___ $ leads to the following state of the society:			
in Dollars	**New Benefit**	**Compared to Current State**	**New Asset (Benefit + Initial Asset)**
**Patient**	0.6	+0.35	0.6
**2 Payers**			
**2 Investors**			
**Seller**	1.2	0	2.4
**Regulator**	1.2	0	2.4

Please select your maximum price by moving the red slider below.
You will see the related consequences in the table above.
If the values do not change, please click the red slider again.

Your previous decisions:
8 months: ${q://QID313/ChoiceNumericEntryValue/1} $
10 months: ${q://QID317/ChoiceNumericEntryValue/1} $

These page timer metrics will not be displayed to the recipient.
First Click: *0 seconds*
Last Click: *0 seconds*
Page Submit: *0 seconds*
Click Count: *0 clicks*

Decision situation (4 of 5):

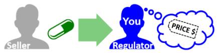

Now the Pharma Company offers a new pharmaceutical treatment which prolongs the survival of the patient by **fifteen** months (compared to no treatment), increasing the life expectancy by an additional **ten** months compared to the current standard treatment. The treatment does not increase the quality of life compared to the standard treatment.

This work is licensed under a Creative Commons Attribution 4.0 International License. Survey created with Qualtrics.

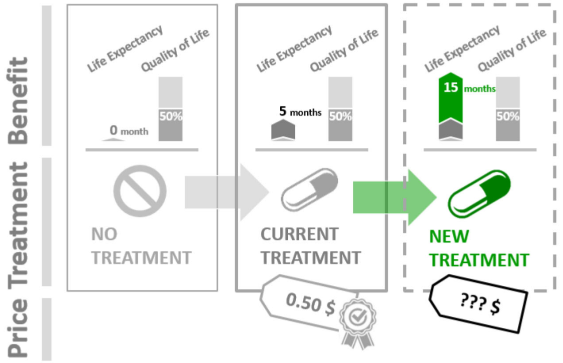

You expect the offer from the pharma company shortly. Before you enter into a negotiation, you should decide on the **absolute maximum price, which you would still consider reasonable and fair for the new product:**

Your maximum price of ___ $ leads to the following state of the society:			
in Dollars	**New Benefit**	**Compared to Current State**	**New Asset (Benefit + Initial Asset)**
**Patient**	0.75	+0.5	0.75
**2 Payers**			
**2 Investors**			
**Seller**	1.2	0	2.4
**Regulator**	1.2	0	2.4

[illegible]

Please select <u>your maximum price</u> by moving the red slider below.
You will see the related consequences in the table above.
If the values do not change, please click the red slider again.

Your previous decisions:
8 months: ${q://QID313/ChoiceNumericEntryValue/1} $
10 months: ${q://QID317/ChoiceNumericEntryValue/1} $
12 months: ${q://QID322/ChoiceNumericEntryValue/1} $

These page timer metrics will not be displayed to the recipient.
First Click: *0 seconds*
Last Click: *0 seconds*
Page Submit: *0 seconds*
Click Count: *0 clicks*

Decision situation (5 of 5):

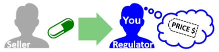

Now the Pharma Company offers a new pharmaceutical treatment which prolongs the survival of the patient by **seventeen** months (compared to no treatment), increasing the life expectancy by an additional **twelve** months compared to the current standard treatment. The treatment does not increase the quality of life compared to the standard treatment.

This work is licensed under a Creative Commons Attribution 4.0 International License. Survey created with Qualtrics.

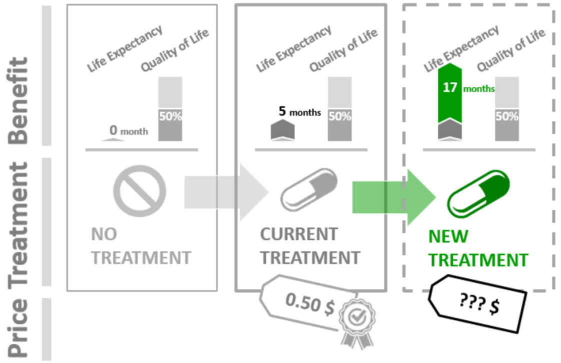

You expect the offer from the pharma company shortly. Before you enter into a negotiation, you should decide on the **absolute maximum price, which you would still consider reasonable and fair for the new product:**

Your maximum price of ___ $ leads to the following state of the society:			
in Dollars	**New Benefit**	**Compared to Current State**	**New Asset (Benefit + Initial Asset)**
**Patient**	0.85	+0.6	0.85
**2 Payers**			
**2 Investors**			
**Seller**	1.2	0	2.4
**Regulator**	1.2	0	2.4

[illegible]

Please select <u>your maximum price</u> by moving the red slider below.
You will see the related consequences in the table above.
If the values do not change, please click the red slider again.

Your previous decisions:
8 months: ${q://QID313/ChoiceNumericEntryValue/1} $
10 months: ${q://QID317/ChoiceNumericEntryValue/1} $
12 months: ${q://QID322/ChoiceNumericEntryValue/1} $
15 months: ${q://QID327/ChoiceNumericEntryValue/1} $

These page timer metrics will not be displayed to the recipient.
First Click: *0 seconds*
Last Click: *0 seconds*
Page Submit: *0 seconds*
Click Count: *0 clicks*

Decision situation (1 of 1):

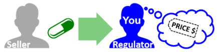

Now the Pharma Company offers a new treatment which prolongs the survival of the patient by minus **one month** (compared to no treatment), decreasing the life expectancy below **zero**. This obviously makes no sense. We just need to test, whether participants read the relevant information. If you did so, please click on the green pill below and submit without entering a price.

This work is licensed under a Creative Commons Attribution 4.0 International License. Survey created with Qualtrics.

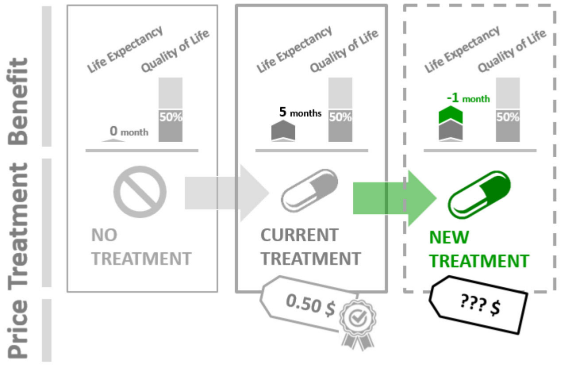

You expect the offer from the pharma company shortly. Before you enter into a negotiation, you should decide on the **absolute maximum price, which you would still consider reasonable and fair for the new product:**

Your maximum price of ___ $ leads to the following state of the society:			
in Dollars	**New Benefit**	**Compared to Current State**	**New Asset (Benefit + Initial Asset)**
**Patient**	-0.05	-0.30	-0.30
**2 Payers**			
**2 Investors**			
**Seller**	1.2	0	2.4
**Regulator**	1.2	0	2.4

[illegible]

Please select your maximum price by moving the red slider below.
You will see the related consequences in the table above.
If the values do not change, please click the red slider again.

These page timer metrics will not be displayed to the recipient.
First Click: *0 seconds*
Last Click: *0 seconds*
Page Submit: *0 seconds*
Click Count: *0 clicks*

For the past rounds: please indicate in descending order the relevance of the stakeholders for your decision.
You can drag and drop the options below to bring them in your preferred order.

Premium payers
Investors
Own role
Negotiation partner
Patient

This work is licensed under a Creative Commons Attribution 4.0 International License. Survey created with Qualtrics.

These page timer metrics will not be displayed to the recipient.
First Click: *0 seconds*
Last Click: *0 seconds*
Page Submit: *0 seconds*
Click Count: *0 clicks*

Regulator - 1 - Price Offer

We will revisit the five new pharmaceutical products of round 1 to 5. But now you can state an actual price expectation to the seller. The seller himself will do the same. Your counterpart is another participant, randomly selected after the experiment.

If your expectation is **higher or equal** to the price offer of the seller, you will both agree. In consequence, the patient will get access to the new treatment with all its benefits (increased life expectancy, economic benefit). The investors will receive the price (revenue) and the payers will have to pay the price (cost). If no agreement is reached, the product will not be available in this country.

You and your counterpart will both receive **an additional bonus for reaching an agreement: you can keep the difference** between your successful price statement and your absolute maximum price (stated in the first part of the experiment). Hence, the lower your price statement compared to your absolute maximum price, the higher your bonus – if the offer is not below the seller's price statement. Then again, the higher your price expectation, the higher potentially the chance of reaching an agreement with the seller.

Below the decision table you will see for each round your stated maximum prices from the first part of the experiment.

Ok? Let us start!

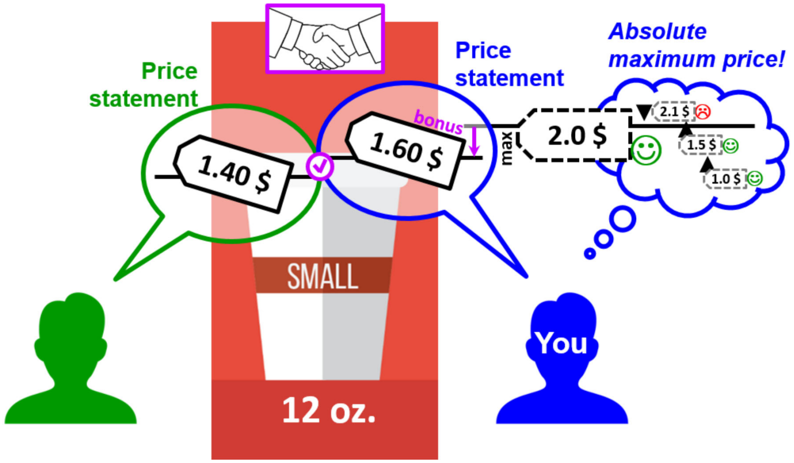

[Coffee cup adjusted from wikiHow: How to Order Coffee. wikiHow Inc.; 2019. The image is licensed under a Creative Commons Attribution-NonCommercial-ShareAlike 3.0 Unported License.]

Decision situation (1 of 5):

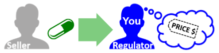

The Pharma Company offers a new pharmaceutical treatment which prolongs the survival of the patient by **eight** months (compared to no treatment), increasing the life expectancy by an additional **three** months compared to the current standard therapy. The treatment does not increase the quality of life compared to the standard treatment.

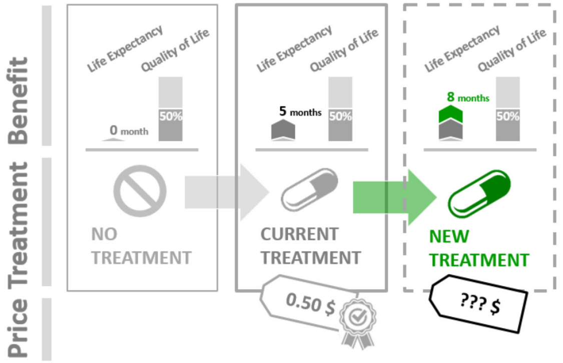

Please chose your price statement to the seller for this product (the maximum prices that you have defined before are displayed below the decision table).

Your maximum price of ___ $<br>leads to the following state of the society:			
in Dollars	**New Benefit**	**Compared to Current State**	**New Asset (Benefit + Initial Asset)**
**Patient**	0.4	+0.15	0.4
**2 Payers**			
**2 Investors**			
**Seller**	1.2	0	2.4
**Regulator**	1.2	0	2.4

[illegible]

Please state your price by moving the red slider below. You will see the related consequences in the table above.
If the values do not change, please click the red slider again.

Your maximum prices form the first five rounds:
8 months: ${q://QID313/ChoiceNumericEntryValue/1} $
10 months: ${q://QID317/ChoiceNumericEntryValue/1} $
12 months: ${q://QID322/ChoiceNumericEntryValue/1} $
15 months: ${q://QID327/ChoiceNumericEntryValue/1} $
17 months: ${q://QID332/ChoiceNumericEntryValue/1} $

These page timer metrics will not be displayed to the recipient.
First Click: *0 seconds*
Last Click: *0 seconds*
Page Submit: *0 seconds*
Click Count: *0 clicks*

Decision situation (2 of 5):

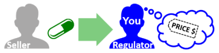

Now the Pharma Company offers a new pharmaceutical treatment which prolongs the survival of the patient by **ten** months (compared to no treatment), increasing the life expectancy by an additional **five** months compared to the current standard treatment. The treatment does not increase the quality of life compared to the standard treatment.

This work is licensed under a Creative Commons Attribution 4.0 International License. Survey created with Qualtrics.

Social Preferences in Reimbursement Negotiations, Additional file 4 Wettstein D., Boes S. 2021

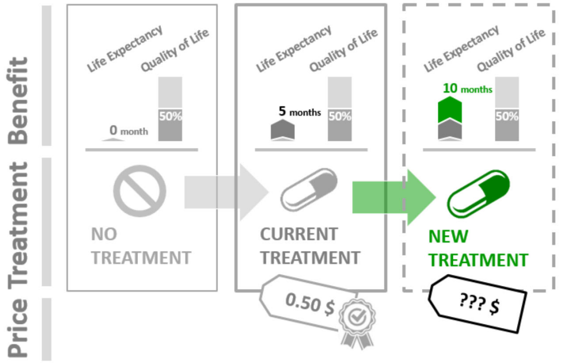

Please chose your price statement to the seller for this product (the maximum prices that you have defined before are displayed below the decision table).

Your maximum price of ___ $<br>leads to the following state of the society:			
in Dollars	**New Benefit**	**Compared to Current State**	**New Asset (Benefit + Initial Asset)**
**Patient**	0.5	+0.25	0.5
**2 Payers**			
**2 Investors**			
**Seller**	1.2	0	2.4
**Regulator**	1.2	0	2.4

[illegible]

Please state your price by moving the red slider below. You will see the related consequences in the table above.
If the values do not change, please click the red slider again.

Your maximum prices form the first five rounds:
8 months: ${q://QID313/ChoiceNumericEntryValue/1} $
10 months: ${q://QID317/ChoiceNumericEntryValue/1} $
12 months: ${q://QID322/ChoiceNumericEntryValue/1} $
15 months: ${q://QID327/ChoiceNumericEntryValue/1} $
17 months: ${q://QID332/ChoiceNumericEntryValue/1} $

These page timer metrics will not be displayed to the recipient.
First Click: *0 seconds*
Last Click: *0 seconds*
Page Submit: *0 seconds*
Click Count: *0 clicks*

Decision situation (3 of 5):

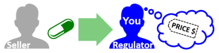

Now the Pharma Company offers a new pharmaceutical treatment which prolongs the survival of the patient by **twelve** months (compared to no treatment), increasing the life expectancy by an additional **seven** months compared to the current standard treatment. The treatment does not increase the quality of life compared to the standard treatment.

This work is licensed under a Creative Commons Attribution 4.0 International License. Survey created with Qualtrics.

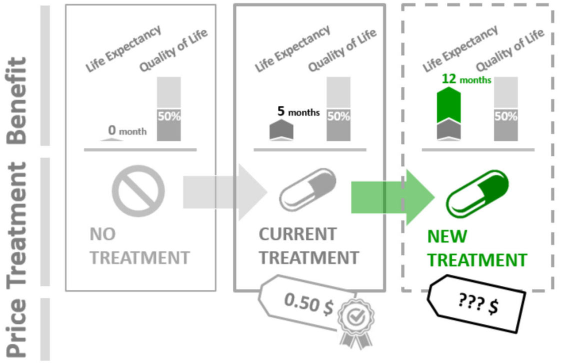

Please chose your price statement to the seller for this product (the maximum prices that you have defined before are displayed below the decision table).

Your maximum price of ___ $ leads to the following state of the society:			
in Dollars	**New Benefit**	**Compared to Current State**	**New Asset (Benefit + Initial Asset)**
**Patient**	0.6	+0.35	0.6
**2 Payers**			
**2 Investors**			
**Seller**	1.2	0	2.4
**Regulator**	1.2	0	2.4

[illegible]

Please state your price by moving the red slider below. You will see the related consequences in the table above.
If the values do not change, please click the red slider again.

Your maximum prices form the first five rounds:
8 months: ${q://QID313/ChoiceNumericEntryValue/1} $
10 months: ${q://QID317/ChoiceNumericEntryValue/1} $
12 months: ${q://QID322/ChoiceNumericEntryValue/1} $
15 months: ${q://QID327/ChoiceNumericEntryValue/1} $
17 months: ${q://QID332/ChoiceNumericEntryValue/1} $

These page timer metrics will not be displayed to the recipient.
First Click: *0 seconds*
Last Click: *0 seconds*
Page Submit: *0 seconds*
Click Count: *0 clicks*

Decision situation (4 of 5):

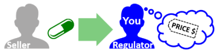

Now the Pharma Company offers a new pharmaceutical treatment which prolongs the survival of the patient by **fifteen** months (compared to no treatment), increasing the life expectancy by an additional **ten** months compared to the current standard treatment. The treatment does not increase the quality of life compared to the standard treatment.

This work is licensed under a Creative Commons Attribution 4.0 International License. Survey created with Qualtrics.

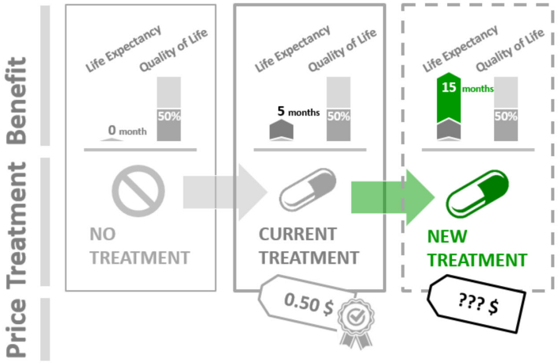

Please chose your price statement to the seller for this product (the maximum prices that you have defined before are displayed below the decision table).

Your maximum price of ___ $ leads to the following state of the society:			
in Dollars	**New Benefit**	**Compared to Current State**	**New Asset (Benefit + Initial Asset)**
**Patient**	0.75	+0.5	0.75
**2 Payers**			
**2 Investors**			
**Seller**	1.2	0	2.4
**Regulator**	1.2	0	2.4

[illegible]

Please state your price by moving the red slider below. You will see the related consequences in the table above.
If the values do not change, please click the red slider again.

Your maximum prices form the first five rounds:
8 months: ${q://QID313/ChoiceNumericEntryValue/1} $
10 months: ${q://QID317/ChoiceNumericEntryValue/1} $
12 months: ${q://QID322/ChoiceNumericEntryValue/1} $
15 months: ${q://QID327/ChoiceNumericEntryValue/1} $
17 months: ${q://QID332/ChoiceNumericEntryValue/1} $

These page timer metrics will not be displayed to the recipient.
First Click: *0 seconds*
Last Click: *0 seconds*
Page Submit: *0 seconds*
Click Count: *0 clicks*

Decision situation (5 of 5):

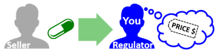

Now the Pharma Company offers a new pharmaceutical treatment which prolongs the survival of the patient by **seventeen** months (compared to no treatment), increasing the life expectancy by an additional **twelve** months compared to the current standard treatment. The treatment does not increase the quality of life compared to the standard treatment.

This work is licensed under a Creative Commons Attribution 4.0 International License. Survey created with Qualtrics.

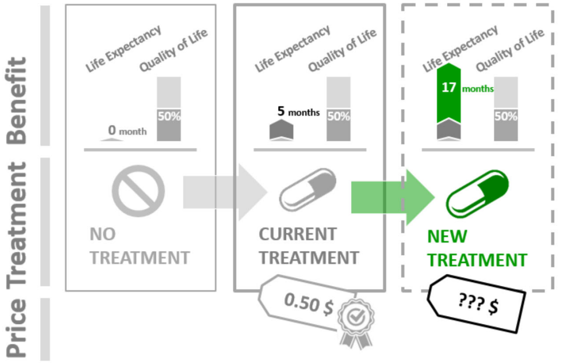

Please chose your price statement to the seller for this product (the maximum prices that you have defined before are displayed below the decision table).

Your maximum price of ___ $ leads to the following state of the society:			
in Dollars	New Benefit	**Compared to Current State**	**New Asset (Benefit + Initial Asset)**
**Patient**	0.85	+0.6	0.85
**2 Payers**			
**2 Investors**			
**Seller**	1.2	0	2.4
**Regulator**	1.2	0	2.4

[illegible]

Please state your price by moving the red slider below. You will see the related consequences in the table above. *If the values do not change, please click the red slider again.*

Your maximum prices form the first five rounds:
8 months: ${q://QID313/ChoiceNumericEntryValue/1} $
10 months: ${q://QID317/ChoiceNumericEntryValue/1} $
12 months: ${q://QID322/ChoiceNumericEntryValue/1} $
15 months: ${q://QID327/ChoiceNumericEntryValue/1} $
17 months: ${q://QID332/ChoiceNumericEntryValue/1} $

These page timer metrics will not be displayed to the recipient.
First Click: *0 seconds*
Last Click: *0 seconds*
Page Submit: *0 seconds*
Click Count: *0 clicks*

For the past five rounds: please indicate in descending order the relevance of the stakeholders for your decision.
You can drag and drop the options below to bring them in your preferred order.

Premium payers
Investors
Patient
Own role

This work is licensed under a Creative Commons Attribution 4.0 International License. Survey created with Qualtrics.

Negotiation partner

These page timer metrics will not be displayed to the recipient.
First Click: *0 seconds*
Last Click: *0 seconds*
Page Submit: *0 seconds*
Click Count: *0 clicks*

Seller - 100'000 - Reservation Price

Let us start with the experiment!

These page timer metrics will not be displayed to the recipient.
First Click: *0 seconds*
Last Click: *0 seconds*
Page Submit: *0 seconds*
Click Count: *0 clicks*

Decision situation (1 of 5):

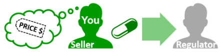

Your company developed a new treatment which prolongs the survival of the patient by **eight** months (compared to no treatment), increasing the life expectancy by an additional **three** months compared to the current standard treatment. The treatment does not increase the quality of life compared to the standard treatment.

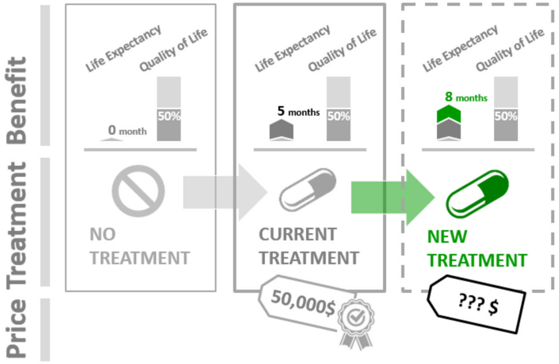

You have to prepare an offer for the Health minister shortly. Before you enter into a negotiation, you should decide on the **absolute minimum price, which you would still consider reasonable and fair for the new product:**

This work is licensed under a Creative Commons Attribution 4.0 International License. Survey created with Qualtrics.

Your minimum price of ___,000 $ leads to the following state of the society:			
in thousand Dollars	**New Benefit**	**Compared to Current State**	**New Asset (Benefit + Initial Asset)**
**Patient**	40	+15	40
**2 Payers**			
**2 Investors**			
**Seller**	120	0	240
**Regulator**	120	0	240

[illegible]

Please select <u>your minimum price</u> by moving the red slider below.
You will see the related consequences in the table above.
If the values do not change, please click the red slider again.

These page timer metrics will not be displayed to the recipient.
First Click: *0 seconds*
Last Click: *0 seconds*
Page Submit: *0 seconds*
Click Count: *0 clicks*

Decision situation (2 of 5):

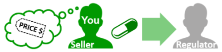

Now your company developed a new treatment which prolongs the survival of the patient by **ten** months (compared to no treatment), increasing the life expectancy by an additional **five** months compared to the current standard treatment. The treatment does not increase the quality of life compared to the standard treatment.

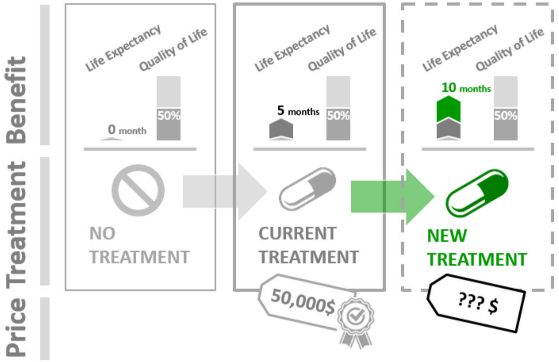

You have to prepare an offer for the Health minister shortly. Before you enter into a negotiation, you should decide on the **absolute minimum price, which you would still consider reasonable and fair for the new product:**

This work is licensed under a Creative Commons Attribution 4.0 International License. Survey created with Qualtrics.

Your minimum price of ___,000 $ leads to the following state of the society:			
in thousand Dollars	**New Benefit**	**Compared to Current State**	**New Asset (Benefit + Initial Asset)**
**Patient**	50	+25	50
**2 Payers**			
**2 Investors**			
**Seller**	120	0	240
**Regulator**	120	0	240

[illegible]

Please select <u>your minimum price</u> by moving the red slider below.
You will see the related consequences in the table above.
If the values do not change, please click the red slider again.

Your previous decision:
8 months: ${q://QID112/ChoiceNumericEntryValue/1},000 $

These page timer metrics will not be displayed to the recipient.
First Click: *0 seconds*
Last Click: *0 seconds*
Page Submit: *0 seconds*
Click Count: *0 clicks*

Decision situation (3 of 5):

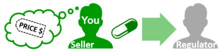

Now your company developed a new treatment which prolongs the survival of the patient by **twelve** months (compared to no treatment), increasing the life expectancy by an additional **seven** months compared to the current standard treatment. The treatment does not increase the quality of life compared to the standard treatment.

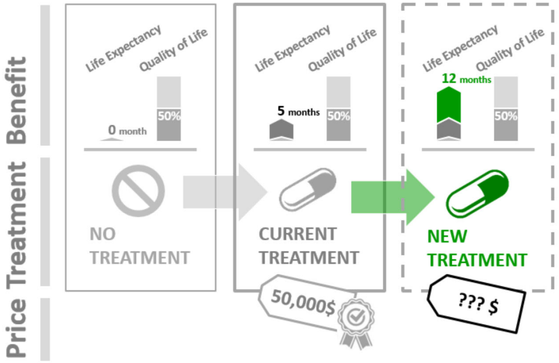

You have to prepare an offer for the Health minister shortly. Before you enter into a negotiation, you should decide on the **absolute minimum price, which you would still consider reasonable and fair for the new product:**

This work is licensed under a Creative Commons Attribution 4.0 International License. Survey created with Qualtrics.

Your maximum price of ___,000 $ leads to the following state of the society:			
in thousand Dollars	New Benefit	Compared to Current State	New Asset (Benefit + Initial Asset)
**Patient**	60	+35	60
**2 Payers**			
**2 Investors**			
**Seller**	120	0	240
**Regulator**	120	0	240

[illegible]

Please select your minimum price by moving the red slider below.
You will see the related consequences in the table above.
If the values do not change, please click the red slider again.

Your previous decisions:
8 months: ${q://QID112/ChoiceNumericEntryValue/1},000 $
10 months: ${q://QID109/ChoiceNumericEntryValue/1},000 $

These page timer metrics will not be displayed to the recipient.
First Click: *0 seconds*
Last Click: *0 seconds*
Page Submit: *0 seconds*
Click Count: *0 clicks*

Decision situation (4 of 5):

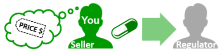

Now your company developed a new treatment which prolongs the survival of the patient by **fifteen** months (compared to no treatment), increasing the life expectancy by an additional **ten** months compared to the current standard treatment. The treatment does not increase the quality of life compared to the standard treatment.

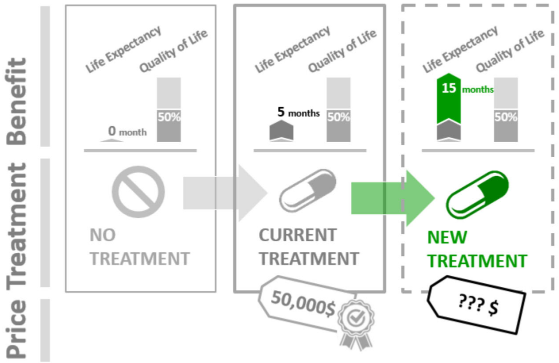

You have to prepare an offer for the Health minister shortly. Before you enter into a negotiation, you should decide on the **absolute minimum price, which you would still consider reasonable and fair for the new product:**

This work is licensed under a Creative Commons Attribution 4.0 International License. Survey created with Qualtrics.

Your minimum price of ___,000 $ leads to the following state of the society:			
in thousand Dollars	New Benefit	Compared to Current State	New Asset (Benefit + Initial Asset)
**Patient**	75	+50	75
**2 Payers**			
**2 Investors**			
**Seller**	120	0	240
**Regulator**	120	0	240

[illegible]

Please select your minimum price by moving the red slider below.
You will see the related consequences in the table above.
If the values do not change, please click the red slider again.

Your previous decisions:
8 months: ${q://QID112/ChoiceNumericEntryValue/1},000 $
10 months: ${q://QID109/ChoiceNumericEntryValue/1},000 $
12 months: ${q://QID113/ChoiceNumericEntryValue/1},000 $

These page timer metrics will not be displayed to the recipient.
First Click: *0 seconds*
Last Click: *0 seconds*
Page Submit: *0 seconds*
Click Count: *0 clicks*

Decision situation (5 of 5):

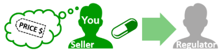

Now your company developed a new treatment which prolongs the survival of the patient by **seventeen** months (compared to no treatment), increasing the life expectancy by an additional **twelve** months compared to the current standard treatment. The treatment does not increase the quality of life compared to the standard treatment.

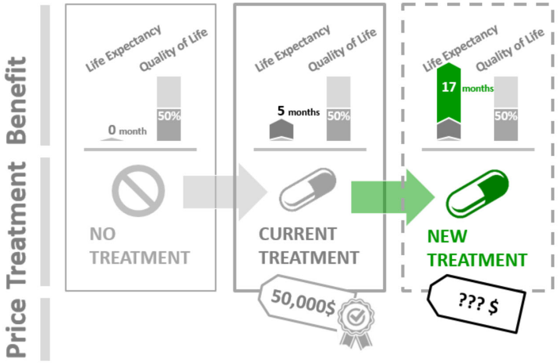

You have to prepare an offer for the Health minister shortly. Before you enter into a negotiation, you should decide on the **absolute minimum price, which you would still consider reasonable and fair for the new product:**

This work is licensed under a Creative Commons Attribution 4.0 International License. Survey created with Qualtrics.

Your minimum price of ___,000 $ leads to the following state of the society:			
in thousand Dollars	**New Benefit**	**Compared to Current State**	**New Asset (Benefit + Initial Asset)**
**Patient**	85	+60	85
**2 Payers**			
**2 Investors**			
**Seller**	120	0	240
**Regulator**	120	0	240

[illegible]

Please select your minimum price by moving the red slider below. You will see the related consequences in the table above. *If the values do not change, please click the red slider again.*

Your previous decisions:
8 months: ${q://QID112/ChoiceNumericEntryValue/1},000 $
10 months: ${q://QID109/ChoiceNumericEntryValue/1},000 $
12 months: ${q://QID113/ChoiceNumericEntryValue/1},000 $
15 months: ${q://QID114/ChoiceNumericEntryValue/1},000 $

These page timer metrics will not be displayed to the recipient.
First Click: *0 seconds*
Last Click: *0 seconds*
Page Submit: *0 seconds*
Click Count: *0 clicks*

Decision situation (1 of 1):

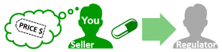

Now your Company developed a new treatment which prolongs the survival of the patient by minus **one month** (compared to no treatment), decreasing the life expectancy below **zero**. This obviously makes no sense. We just need to test, whether participants read the relevant information. If you did so, please click on the green pill below and submit without entering a price.

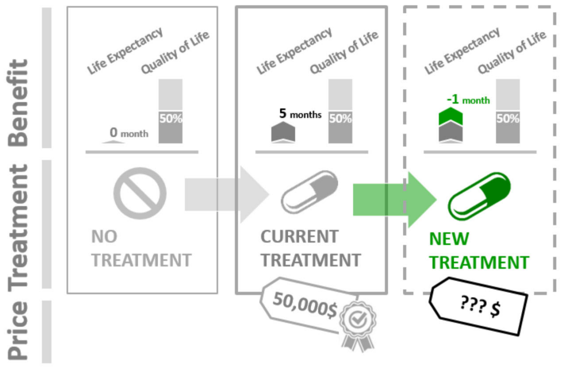

You have to prepare an offer for the Health minister shortly. Before you enter into a negotiation, you should decide on the **absolute minimum price, which you would still consider reasonable and fair for the new product:**

This work is licensed under a Creative Commons Attribution 4.0 International License. Survey created with Qualtrics.

Your minimum price of ___,000 $ leads to the following state of the society:			
in thousand Dollars	**New Benefit**	**Compared to Current State**	**New Asset (Benefit + Initial Asset)**
**Patient**	-5	-30	-30
**2 Payers**			
**2 Investors**			
**Seller**	120	0	240
**Regulator**	120	0	240

[illegible]

Please select your minimum price by moving the red slider below. You will see the related consequences in the table above. *If the values do not change, please click the red slider again.*

These page timer metrics will not be displayed to the recipient.
First Click: *0 seconds*
Last Click: *0 seconds*
Page Submit: *0 seconds*
Click Count: *0 clicks*

For the past rounds: please indicate in descending order the relevance of the stakeholders for your decision.
You can drag and drop the options below to bring them in your preferred order.

Patient

Premium payers

Own role

Negotiation partner

Investors

These page timer metrics will not be displayed to the recipient.
First Click: *0 seconds*
Last Click: *0 seconds*
Page Submit: *0 seconds*
Click Count: *0 clicks*

Seller - 100'000 - Price Offer

We will revisit the five new pharmaceutical products of round 1 to 5. But now you can place an actual price offer to the regulator. The regulator himself will do the same and state his price expectation. Your counterpart is another participant, randomly selected after the experiment.

If your offer is **lower or equal** to the price expectation of the regulator, you will both agree. In consequence, the patient will get access to the new treatment with all its benefits (increased life expectancy, economic benefit). The investors will receive the price (revenue) and the payers will have to pay the price (cost). If no agreement is reached, the product will not be available in this country.

You and your counterpart will both receive **an additional bonus for reaching an agreement: you can keep the difference** between your successful price offer and your absolute minimum price (stated in the first part of the experiment). Hence, the higher your price offer compared to your absolute minimum price, the higher your bonus – if the offer is not beyond the regulator's price statement. Then again, the lower your price offer, the higher potentially the chance of reaching an agreement with the regulator.

Below the decision table you will see for each round your stated minimum prices from the first part of the experiment.

Ok? Let us start!

This work is licensed under a Creative Commons Attribution 4.0 International License. Survey created with Qualtrics.

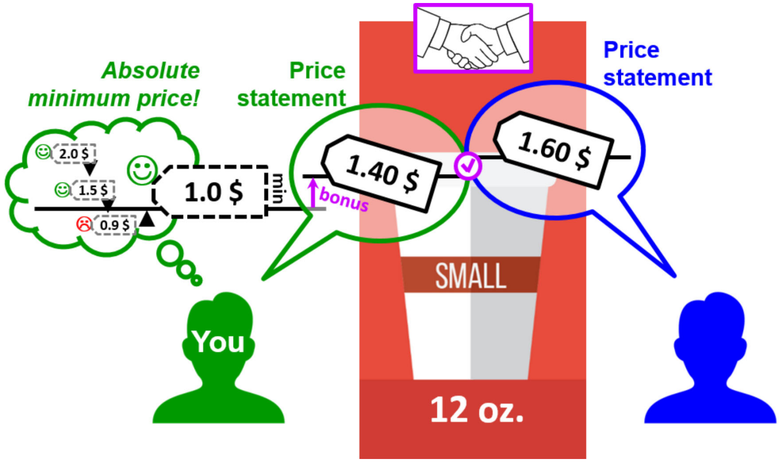

[Coffee cup adjusted from wikiHow: How to Order Coffee. wikiHow Inc.; 2019. The image is licensed under a Creative Commons Attribution-NonCommercial-ShareAlike 3.0 Unported License.]

Decision situation (1 of 5):

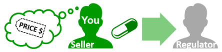

Your company developed a new treatment which prolongs the survival of the patient by **eight** months (compared to no treatment), increasing the life expectancy by an additional **three** months compared to the current standard treatment. The treatment does not increase the quality of life compared to the standard treatment.

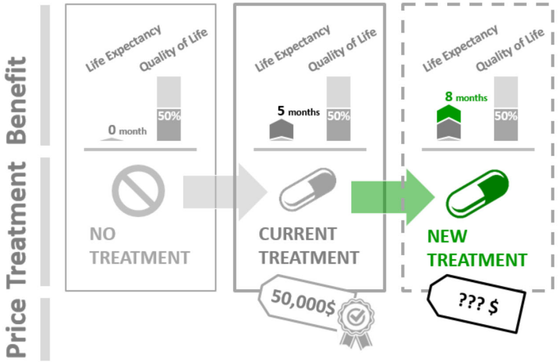

Please chose your price statement to the regulator for this product (the minimum prices that you have defined before are displayed below the decision table).

Your minimum price of ___,000 $ leads to the following state of the society:			
in thousand Dollars	**New Benefit**	**Compared to Current State**	**New Asset (Benefit + Initial Asset)**
**Patient**	40	+15	40
**2 Payers**			
**2 Investors**			
**Seller**	120	0	240
**Regulator**	120	0	240

[illegible]

Please state your price by moving the red slider below. You will see the related consequences in the table above. *If the values do not change, please click the red slider again.*

Your minimum prices form the first five rounds:
8 months: ${q://QID112/ChoiceNumericEntryValue/1},000 $
10 months: ${q://QID109/ChoiceNumericEntryValue/1},000 $
12 months: ${q://QID113/ChoiceNumericEntryValue/1},000 $
15 months: ${q://QID114/ChoiceNumericEntryValue/1},000 $
17 months: ${q://QID115/ChoiceNumericEntryValue/1},000 $

These page timer metrics will not be displayed to the recipient.
First Click: *0 seconds*
Last Click: *0 seconds*
Page Submit: *0 seconds*
Click Count: *0 clicks*

Decision situation (2 of 5):

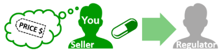

Now your company developed a new treatment which prolongs the survival of the patient by **ten** months (compared to no treatment), increasing the life expectancy by an additional **five** months compared to the current standard treatment. The treatment does not increase the quality of life compared to the standard treatment.

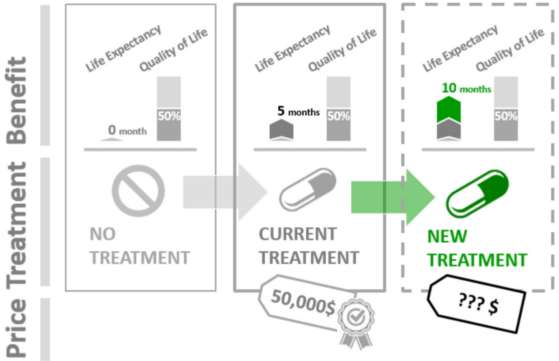

Please chose your price statement to the regulator for this product (the minimum prices that you have defined before are displayed below the decision table).

This work is licensed under a Creative Commons Attribution 4.0 International License. Survey created with Qualtrics.

Your minimum price of ___,000 $ leads to the following state of the society:			
in thousand Dollars	**New Benefit**	**Compared to Current State**	**New Asset (Benefit + Initial Asset)**
**Patient**	50	+25	50
**2 Payers**			
**2 Investors**			
**Seller**	120	0	240
**Regulator**	120	0	240

[illegible]

Please state your price by moving the red slider below. You will see the related consequences in the table above. *If the values do not change, please click the red slider again.*

Your minimum prices form the first five rounds:
8 months: ${q://QID112/ChoiceNumericEntryValue/1},000 $
10 months: ${q://QID109/ChoiceNumericEntryValue/1},000 $
12 months: ${q://QID113/ChoiceNumericEntryValue/1},000 $
15 months: ${q://QID114/ChoiceNumericEntryValue/1},000 $
17 months: ${q://QID115/ChoiceNumericEntryValue/1},000 $

These page timer metrics will not be displayed to the recipient.
First Click: *0 seconds*
Last Click: *0 seconds*
Page Submit: *0 seconds*
Click Count: *0 clicks*

Decision situation (3 of 5):

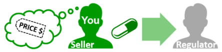

Now your company developed a new treatment which prolongs the survival of the patient by **twelve** months (compared to no treatment), increasing the life expectancy by an additional **seven** months compared to the current standard treatment. The treatment does not increase the quality of life compared to the standard treatment.

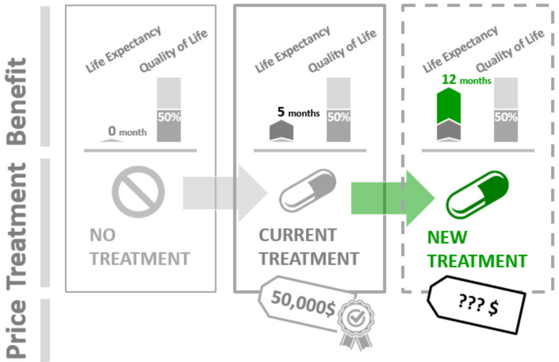

Please chose your price statement to the regulator for this product (the minimum prices that you have defined before are displayed below the decision table).

This work is licensed under a Creative Commons Attribution 4.0 International License. Survey created with Qualtrics.

Your maximum price of ___,000 $ leads to the following state of the society:			
in thousand Dollars	**New Benefit**	**Compared to Current State**	**New Asset (Benefit + Initial Asset)**
**Patient**	60	+35	60
**2 Payers**			
**2 Investors**			
**Seller**	120	0	240
**Regulator**	120	0	240

[illegible]

Please state your price by moving the red slider below. You will see the related consequences in the table above. *If the values do not change, please click the red slider again.*

Your minimum prices form the first five rounds:
8 months: ${q://QID112/ChoiceNumericEntryValue/1},000 $
10 months: ${q://QID109/ChoiceNumericEntryValue/1},000 $
12 months: ${q://QID113/ChoiceNumericEntryValue/1},000 $
15 months: ${q://QID114/ChoiceNumericEntryValue/1},000 $
17 months: ${q://QID115/ChoiceNumericEntryValue/1},000 $

These page timer metrics will not be displayed to the recipient.
First Click: *0 seconds*
Last Click: *0 seconds*
Page Submit: *0 seconds*
Click Count: *0 clicks*

Decision situation (4 of 5):

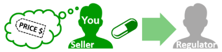

Now your company developed a new treatment which prolongs the survival of the patient by **fifteen** months (compared to no treatment), increasing the life expectancy by an additional **ten** months compared to the current standard treatment. The treatment does not increase the quality of life compared to the standard treatment.

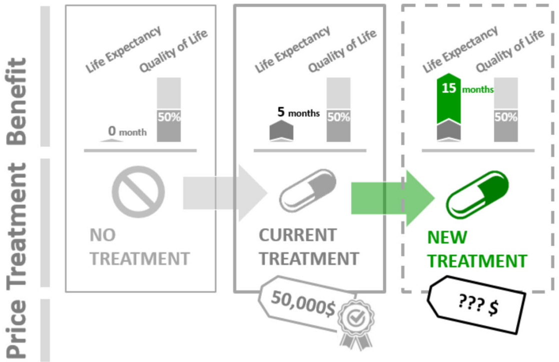

Please chose your price statement to the regulator for this product (the minimum prices that you have defined before are displayed below the decision table).

This work is licensed under a Creative Commons Attribution 4.0 International License. Survey created with Qualtrics.

Your minimum price of ___,000 $ leads to the following state of the society:			
in thousand Dollars	**New Benefit**	**Compared to Current State**	**New Asset (Benefit + Initial Asset)**
**Patient**	75	+50	75
**2 Payers**			
**2 Investors**			
**Seller**	120	0	240
**Regulator**	120	0	240

[illegible]

Please state your price by moving the red slider below. You will see the related consequences in the table above.
If the values do not change, please click the red slider again.

Your minimum prices form the first five rounds:
8 months: ${q://QID112/ChoiceNumericEntryValue/1},000 $
10 months: ${q://QID109/ChoiceNumericEntryValue/1},000 $
12 months: ${q://QID113/ChoiceNumericEntryValue/1},000 $
15 months: ${q://QID114/ChoiceNumericEntryValue/1},000 $
17 months: ${q://QID115/ChoiceNumericEntryValue/1},000 $

These page timer metrics will not be displayed to the recipient.
First Click: *0 seconds*
Last Click: *0 seconds*
Page Submit: *0 seconds*
Click Count: *0 clicks*

Decision situation (5 of 5):

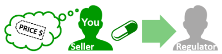

Now your company developed a new treatment which prolongs the survival of the patient by **seventeen** months (compared to no treatment), increasing the life expectancy by an additional **twelve** months compared to the current standard treatment. The treatment does not increase the quality of life compared to the standard treatment.

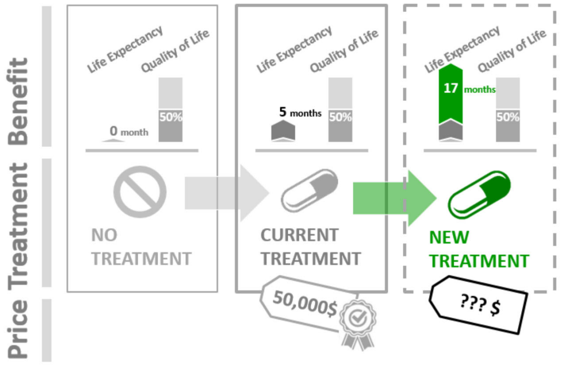

Please chose your price statement to the regulator for this product (the minimum prices that you have defined before are displayed below the decision table).

This work is licensed under a Creative Commons Attribution 4.0 International License. Survey created with Qualtrics.

Your minimum price of ___,000 $ leads to the following state of the society:			
in thousand Dollars	**New Benefit**	**Compared to Current State**	**New Asset (Benefit + Initial Asset)**
**Patient**	85	+60	85
**2 Payers**			
**2 Investors**			
**Seller**	120	0	240
**Regulator**	120	0	240

[illegible]

Please state your price by moving the red slider below. You will see the related consequences in the table above.
If the values do not change, please click the red slider again.

Your minimum prices form the first five rounds:
8 months: ${q://QID112/ChoiceNumericEntryValue/1},000 $
10 months: ${q://QID109/ChoiceNumericEntryValue/1},000 $
12 months: ${q://QID113/ChoiceNumericEntryValue/1},000 $
15 months: ${q://QID114/ChoiceNumericEntryValue/1},000 $
17 months: ${q://QID115/ChoiceNumericEntryValue/1},000 $

These page timer metrics will not be displayed to the recipient.
First Click: *0 seconds*
Last Click: *0 seconds*
Page Submit: *0 seconds*
Click Count: *0 clicks*

For the past five rounds: please indicate in descending order the relevance of the stakeholders for your decision.
You can drag and drop the options below to bring them in your preferred order.

Negotiation partner
Investors
Premium payers
Patient
Own role

These page timer metrics will not be displayed to the recipient.
First Click: *0 seconds*
Last Click: *0 seconds*
Page Submit: *0 seconds*
Click Count: *0 clicks*

Seller - 1 - Reservation Price

Let us start with the experiment!

These page timer metrics will not be displayed to the recipient.
First Click: *0 seconds*
Last Click: *0 seconds*
Page Submit: *0 seconds*
Click Count: *0 clicks*

Decision situation (1 of 5):

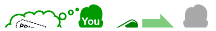

This work is licensed under a Creative Commons Attribution 4.0 International License. Survey created with Qualtrics.

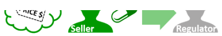

Your company developed a new treatment which prolongs the survival of the patient by **eight** months (compared to no treatment), increasing the life expectancy by an additional **three** months compared to the current standard treatment. The treatment does not increase the quality of life compared to the standard treatment.

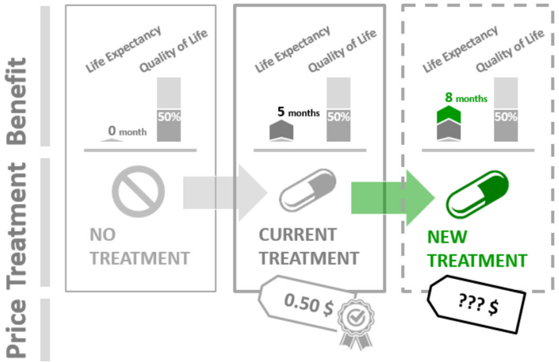

You have to prepare an offer for the Health minister shortly. Before you enter into a negotiation, you should decide on the **absolute minimum price, which you would still consider reasonable and fair for the new product:**

Your minimum price of ___ $ leads to the following state of the society:			
in Dollars	**New Benefit**	**Compared to Current State**	**New Asset (Benefit + Initial Asset)**
**Patient**	0.4	+0.15	0.4
**2 Payers**			
**2 Investors**			
**Seller**	1.2	0	2.4
**Regulator**	1.2	0	2.4

Please select your minimum price by moving the red slider below.
You will see the related consequences in the table above.
If the values do not change, please click the red slider again.

These page timer metrics will not be displayed to the recipient.
First Click: *0 seconds*
Last Click: *0 seconds*
Page Submit: *0 seconds*
Click Count: *0 clicks*

Decision situation (2 of 5):

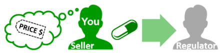

Now your company developed a new treatment which prolongs the survival of the patient by **ten** months (compared to no treatment), increasing the life expectancy by an additional **five** months compared to the current standard treatment. The treatment does not increase the quality of life compared to the standard treatment.

This work is licensed under a Creative Commons Attribution 4.0 International License. Survey created with Qualtrics.

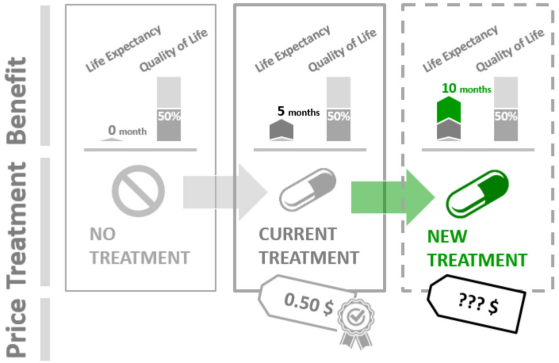

You have to prepare an offer for the Health minister shortly. Before you enter into a negotiation, you should decide on the **absolute minimum price, which you would still consider reasonable and fair for the new product:**

Your minimum price of ___ $ leads to the following state of the society:			
in Dollars	**New Benefit**	**Compared to Current State**	**New Asset (Benefit + Initial Asset)**
**Patient**	0.5	+0.25	0.5
**2 Payers**			
**2 Investors**			
**Seller**	1.2	0	2.4
**Regulator**	1.2	0	2.4

Please select your minimum price by moving the red slider below.
You will see the related consequences in the table above.
If the values do not change, please click the red slider again.

Your previous decision:
8 months: ${q://QID369/ChoiceNumericEntryValue/1} $

These page timer metrics will not be displayed to the recipient.
First Click: *0 seconds*
Last Click: *0 seconds*
Page Submit: *0 seconds*
Click Count: *0 clicks*

Decision situation (3 of 5):

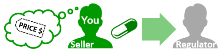

Now your company developed a new treatment which prolongs the survival of the patient by **twelve** months (compared to no treatment), increasing the life expectancy by an additional **seven** months compared to the current standard treatment. The treatment does not increase the quality of life compared to the standard treatment.

This work is licensed under a Creative Commons Attribution 4.0 International License. Survey created with Qualtrics.

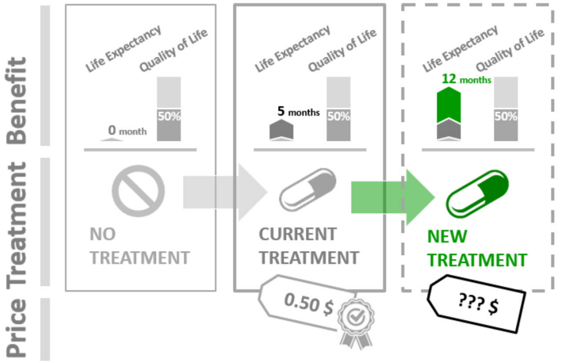

You have to prepare an offer for the Health minister shortly. Before you enter into a negotiation, you should decide on the **absolute minimum price, which you would still consider reasonable and fair for the new product:**

Your maximum price of ___ $<br>leads to the following state of the society:			
in Dollars	**New Benefit**	**Compared to Current State**	**New Asset (Benefit + Initial Asset)**
**Patient**	0.6	+0.35	0.6
**2 Payers**			
**2 Investors**			
**Seller**	1.2	0	2.4
**Regulator**	1.2	0	2.4

[illegible]

Please select your minimum price by moving the red slider below.
You will see the related consequences in the table above.
If the values do not change, please click the red slider again.

Your previous decisions:
8 months: ${q://QID369/ChoiceNumericEntryValue/1} $
10 months: ${q://QID373/ChoiceNumericEntryValue/1} $

These page timer metrics will not be displayed to the recipient.
First Click: *0 seconds*
Last Click: *0 seconds*
Page Submit: *0 seconds*
Click Count: *0 clicks*

Decision situation (4 of 5):

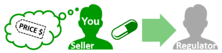

Now your company developed a new treatment which prolongs the survival of the patient by **fifteen** months (compared to no treatment), increasing the life expectancy by an additional **ten** months compared to the current standard treatment. The treatment does not increase the quality of life compared to the standard treatment.

This work is licensed under a Creative Commons Attribution 4.0 International License. Survey created with Qualtrics.

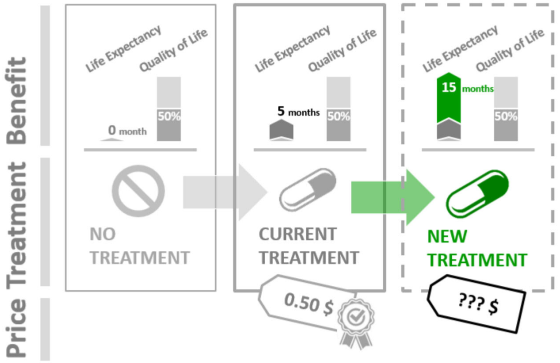

You have to prepare an offer for the Health minister shortly. Before you enter into a negotiation, you should decide on the **absolute minimum price, which you would still consider reasonable and fair for the new product:**

Your minimum price of ___ $<br>leads to the following state of the society:			
in Dollars	**New Benefit**	**Compared to Current State**	**New Asset (Benefit + Initial Asset)**
**Patient**	0.75	+0.5	0.75
**2 Payers**			
**2 Investors**			
**Seller**	1.2	0	2.4
**Regulator**	1.2	0	2.4

[illegible]

Please select your minimum price by moving the red slider below.
You will see the related consequences in the table above.
If the values do not change, please click the red slider again.

Your previous decisions:
8 months: ${q://QID369/ChoiceNumericEntryValue/1} $
10 months: ${q://QID373/ChoiceNumericEntryValue/1} $
12 months: ${q://QID378/ChoiceNumericEntryValue/1} $

These page timer metrics will not be displayed to the recipient.
First Click: *0 seconds*
Last Click: *0 seconds*
Page Submit: *0 seconds*
Click Count: *0 clicks*

Decision situation (5 of 5):

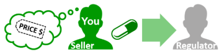

Now your company developed a new treatment which prolongs the survival of the patient by **seventeen** months (compared to no treatment), increasing the life expectancy by an additional **twelve** months compared to the current standard treatment. The treatment does not increase the quality of life compared to the standard treatment.

This work is licensed under a Creative Commons Attribution 4.0 International License. Survey created with Qualtrics.

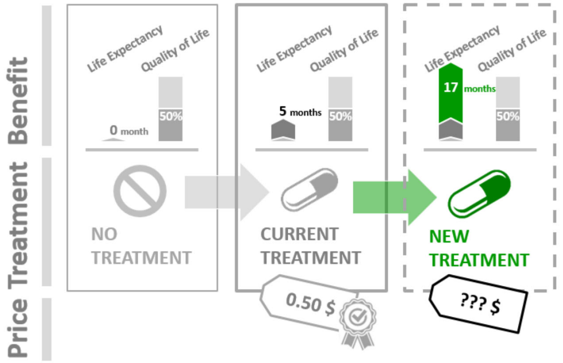

You have to prepare an offer for the Health minister shortly. Before you enter into a negotiation, you should decide on the **absolute minimum price, which you would still consider reasonable and fair for the new product:**

Your minimum price of ___ $ leads to the following state of the society:			
in Dollars	**New Benefit**	**Compared to Current State**	**New Asset (Benefit + Initial Asset)**
**Patient**	0.85	+0.6	0.85
**2 Payers**			
**2 Investors**			
**Seller**	1.2	0	2.4
**Regulator**	1.2	0	2.4

[illegible]

Please select your minimum price by moving the red slider below.
You will see the related consequences in the table above.
If the values do not change, please click the red slider again.

Your previous decisions:
8 months: ${q://QID369/ChoiceNumericEntryValue/1} $
10 months: ${q://QID373/ChoiceNumericEntryValue/1} $
12 months: ${q://QID378/ChoiceNumericEntryValue/1} $
15 months: ${q://QID383/ChoiceNumericEntryValue/1} $

These page timer metrics will not be displayed to the recipient.
First Click: *0 seconds*
Last Click: *0 seconds*
Page Submit: *0 seconds*
Click Count: *0 clicks*

Decision situation (1 of 1):

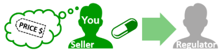

Now your Company developed a new treatment which prolongs the survival of the patient by minus **one month** (compared to no treatment), decreasing the life expectancy below **zero**. This obviously makes no sense. We just need to test, whether participants read the relevant information. If you did so, please click on the green pill below and submit without entering a price.

This work is licensed under a Creative Commons Attribution 4.0 International License. Survey created with Qualtrics.

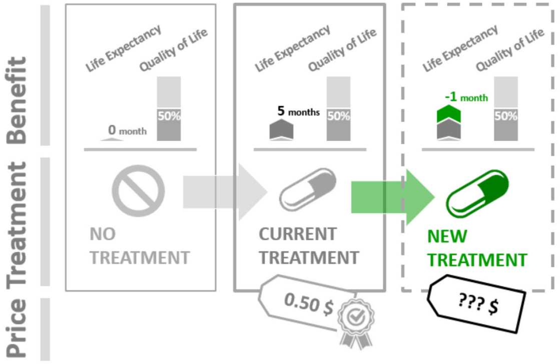

You have to prepare an offer for the Health minister shortly. Before you enter into a negotiation, you should decide on the **absolute minimum price, which you would still consider reasonable and fair for the new product:**

Your minimum price of ___ $ leads to the following state of the society:			
in Dollars	**New Benefit**	**Compared to Current State**	**New Asset (Benefit + Initial Asset)**
**Patient**	-0.05	-0.30	-0.30
**2 Payers**			
**2 Investors**			
**Seller**	1.2	0	2.4
**Regulator**	1.2	0	2.4

[illegible]

Please select your minimum price by moving the red slider below.
You will see the related consequences in the table above.
If the values do not change, please click the red slider again.

These page timer metrics will not be displayed to the recipient.
First Click: *0 seconds*
Last Click: *0 seconds*
Page Submit: *0 seconds*
Click Count: *0 clicks*

For the past rounds: please indicate in descending order the relevance of the stakeholders for your decision.
You can drag and drop the options below to bring them in your preferred order.

Negotiation partner
Patient
Own role
Investors
Premium payers

This work is licensed under a Creative Commons Attribution 4.0 International License. Survey created with Qualtrics.

These page timer metrics will not be displayed to the recipient.
First Click: *0 seconds*
Last Click: *0 seconds*
Page Submit: *0 seconds*
Click Count: *0 clicks*

Seller - 1 - Price Offer

We will revisit the five new pharmaceutical products of round 1 to 5. But now you can place an actual price offer to the regulator. The regulator himself will do the same and state his price expectation. Your counterpart is another participant, randomly selected after the experiment.

If your offer is **lower or equal** to the price expectation of the regulator, you will both agree. In consequence, the patient will get access to the new treatment with all its benefits (increased life expectancy, economic benefit). The investors will receive the price (revenue) and the payers will have to pay the price (cost). If no agreement is reached, the product will not be available in this country.

You and your counterpart will both receive **an additional bonus for reaching an agreement: you can keep the difference** between your successful price offer and your absolute minimum price (stated in the first part of the experiment). Hence, the higher your price offer compared to your absolute minimum price, the higher your bonus – if the offer is not beyond the regulator's price statement. Then again, the lower your price offer, the higher potentially the chance of reaching an agreement with the regulator.

Below the decision table you will see for each round your stated minimum prices from the first part of the experiment.

Ok? Let us start!

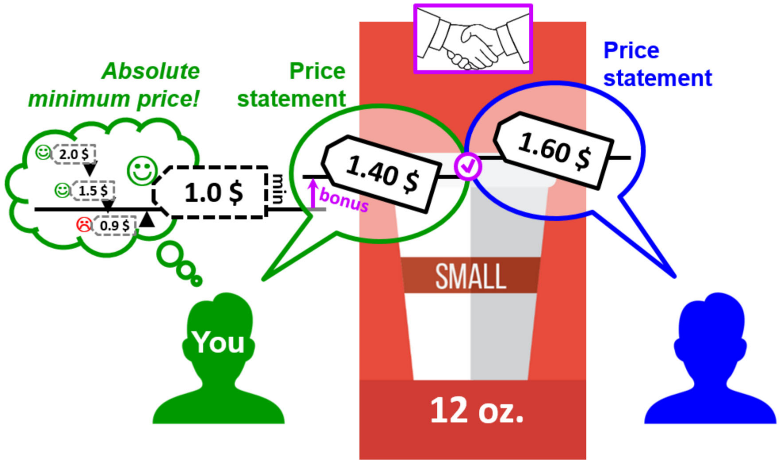

[Coffee cup adjusted from wikiHow: How to Order Coffee. wikiHow Inc.; 2019. The image is licensed under a Creative Commons Attribution-NonCommercial-ShareAlike 3.0 Unported License.]

Decision situation (1 of 5):

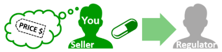

Your company developed a new treatment which prolongs the survival of the patient by **eight** months (compared to no treatment), increasing the life expectancy by an additional **three** months compared to the current standard treatment. The treatment does not increase the quality of life compared to the standard treatment.

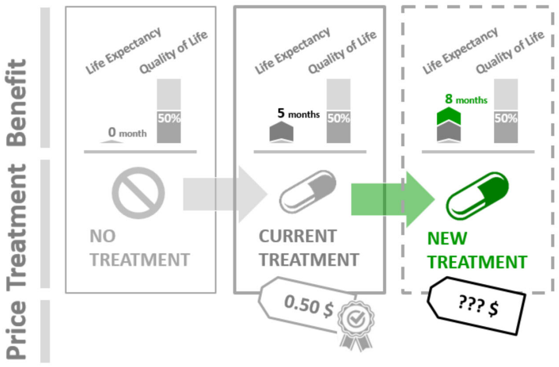

Please chose your price statement to the regulator for this product (the minimum prices that you have defined before are displayed below the decision table).

Your minimum price of ___ $ leads to the following state of the society:			
in Dollars	**New Benefit**	**Compared to Current State**	**New Asset (Benefit + Initial Asset)**
**Patient**	0.4	+0.15	0.4
**2 Payers**			
**2 Investors**			
**Seller**	1.2	0	2.4
**Regulator**	1.2	0	2.4

[illegible]

Please state your price by moving the red slider below. You will see the related consequences in the table above. *If the values do not change, please click the red slider again.*

Your minimum prices form the first five rounds:
8 months: ${q://QID369/ChoiceNumericEntryValue/1} $
10 months: ${q://QID373/ChoiceNumericEntryValue/1} $
12 months: ${q://QID378/ChoiceNumericEntryValue/1} $
15 months: ${q://QID383/ChoiceNumericEntryValue/1} $
17 months: ${q://QID388/ChoiceNumericEntryValue/1} $

These page timer metrics will not be displayed to the recipient.
First Click: *0 seconds*
Last Click: *0 seconds*
Page Submit: *0 seconds*
Click Count: *0 clicks*

Decision situation (2 of 5):

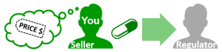

Now your company developed a new treatment which prolongs the survival of the patient by **ten** months (compared to no treatment), increasing the life expectancy by an additional **five** months compared to the current standard treatment. The treatment does not increase the quality of life compared to the standard treatment.

 This work is licensed under a Creative Commons Attribution 4.0 International License. Survey created with Qualtrics.

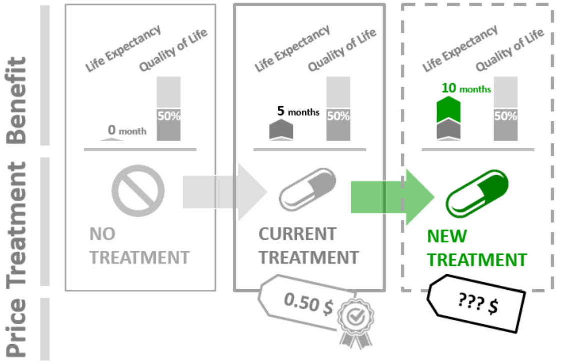

Please chose your price statement to the regulator for this product (the minimum prices that you have defined before are displayed below the decision table).

Your minimum price of ___ $ leads to the following state of the society:			
in Dollars	**New Benefit**	**Compared to Current State**	**New Asset (Benefit + Initial Asset)**
**Patient**	0.5	+0.25	0.5
**2 Payers**			
**2 Investors**			
**Seller**	1.2	0	2.4
**Regulator**	1.2	0	2.4

[illegible]

Please state your price by moving the red slider below. You will see the related consequences in the table above.
If the values do not change, please click the red slider again.

Your minimum prices form the first five rounds:
8 months: ${q://QID369/ChoiceNumericEntryValue/1} $
10 months: ${q://QID373/ChoiceNumericEntryValue/1} $
12 months: ${q://QID378/ChoiceNumericEntryValue/1} $
15 months: ${q://QID383/ChoiceNumericEntryValue/1} $
17 months: ${q://QID388/ChoiceNumericEntryValue/1} $

These page timer metrics will not be displayed to the recipient.
First Click: *0 seconds*
Last Click: *0 seconds*
Page Submit: *0 seconds*
Click Count: *0 clicks*

Decision situation (3 of 5):

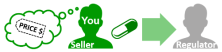

Now your company developed a new treatment which prolongs the survival of the patient by **twelve** months (compared to no treatment), increasing the life expectancy by an additional **seven** months compared to the current standard treatment. The treatment does not increase the quality of life compared to the standard treatment.

This work is licensed under a Creative Commons Attribution 4.0 International License. Survey created with Qualtrics.

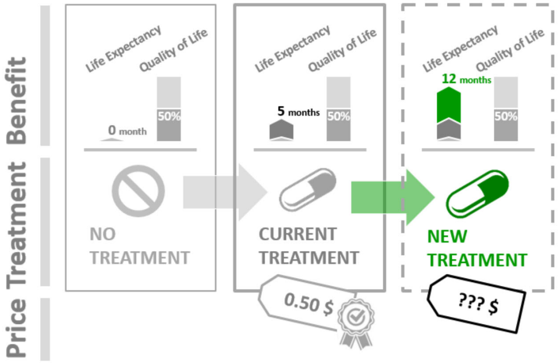

Please chose your price statement to the regulator for this product (the minimum prices that you have defined before are displayed below the decision table).

Your maximum price of ___ $ leads to the following state of the society:			
in Dollars	**New Benefit**	**Compared to Current State**	**New Asset (Benefit + Initial Asset)**
**Patient**	0.6	+0.35	0.6
**2 Payers**			
**2 Investors**			
**Seller**	1.2	0	2.4
**Regulator**	1.2	0	2.4

[illegible]

Please state your price by moving the red slider below. You will see the related consequences in the table above.
If the values do not change, please click the red slider again.

Your minimum prices form the first five rounds:
8 months: ${q://QID369/ChoiceNumericEntryValue/1} $
10 months: ${q://QID373/ChoiceNumericEntryValue/1} $
12 months: ${q://QID378/ChoiceNumericEntryValue/1} $
15 months: ${q://QID383/ChoiceNumericEntryValue/1} $
17 months: ${q://QID388/ChoiceNumericEntryValue/1} $

These page timer metrics will not be displayed to the recipient.
First Click: *0 seconds*
Last Click: *0 seconds*
Page Submit: *0 seconds*
Click Count: *0 clicks*

Decision situation (4 of 5):

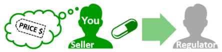

Now your company developed a new treatment which prolongs the survival of the patient by **fifteen** months (compared to no treatment), increasing the life expectancy by an additional **ten** months compared to the current standard treatment. The treatment does not increase the quality of life compared to the standard treatment.

This work is licensed under a Creative Commons Attribution 4.0 International License. Survey created with Qualtrics.

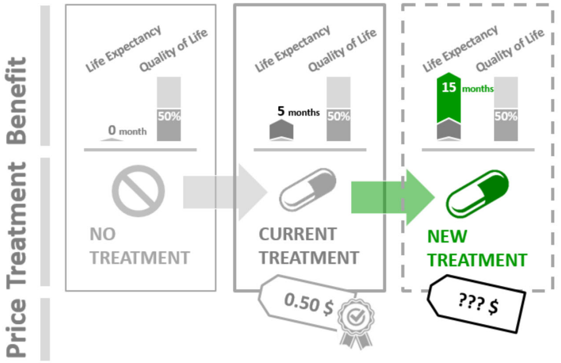

Please chose your price statement to the regulator for this product (the minimum prices that you have defined before are displayed below the decision table).

Your minimum price of ___ $ leads to the following state of the society:			
in Dollars	**New Benefit**	**Compared to Current State**	**New Asset (Benefit + Initial Asset)**
**Patient**	0.75	+0.5	0.75
**2 Payers**			
**2 Investors**			
**Seller**	1.2	0	2.4
**Regulator**	1.2	0	2.4

[illegible]

Please state your price by moving the red slider below. You will see the related consequences in the table above. *If the values do not change, please click the red slider again.*

Your minimum prices form the first five rounds:
8 months: ${q://QID369/ChoiceNumericEntryValue/1} $
10 months: ${q://QID373/ChoiceNumericEntryValue/1} $
12 months: ${q://QID378/ChoiceNumericEntryValue/1} $
15 months: ${q://QID383/ChoiceNumericEntryValue/1} $
17 months: ${q://QID388/ChoiceNumericEntryValue/1} $

These page timer metrics will not be displayed to the recipient.
First Click: *0 seconds*
Last Click: *0 seconds*
Page Submit: *0 seconds*
Click Count: *0 clicks*

Decision situation (5 of 5):

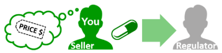

Now your company developed a new treatment which prolongs the survival of the patient by **seventeen** months (compared to no treatment), increasing the life expectancy by an additional **twelve** months compared to the current standard treatment. The treatment does not increase the quality of life compared to the standard treatment.

This work is licensed under a Creative Commons Attribution 4.0 International License. Survey created with Qualtrics.

Social Preferences in Reimbursement Negotiations, Additional file 4 Wettstein D., Boes S. 2021

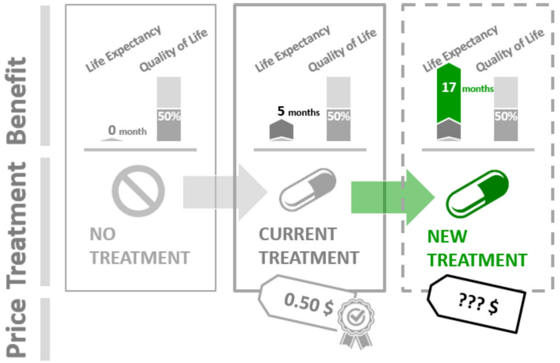

Please chose your price statement to the regulator for this product (the minimum prices that you have defined before are displayed below the decision table).

Your minimum price of ___ $ leads to the following state of the society:			
in Dollars	**New Benefit**	**Compared to Current State**	**New Asset (Benefit + Initial Asset)**
**Patient**	0.85	+0.6	0.85
**2 Payers**			
**2 Investors**			
**Seller**	1.2	0	2.4
**Regulator**	1.2	0	2.4

[illegible]

Please state your price by moving the red slider below. You will see the related consequences in the table above. *If the values do not change, please click the red slider again.*

Your minimum prices form the first five rounds:
8 months: ${q://QID369/ChoiceNumericEntryValue/1} $
10 months: ${q://QID373/ChoiceNumericEntryValue/1} $
12 months: ${q://QID378/ChoiceNumericEntryValue/1} $
15 months: ${q://QID383/ChoiceNumericEntryValue/1} $
17 months: ${q://QID388/ChoiceNumericEntryValue/1} $

These page timer metrics will not be displayed to the recipient.
First Click: *0 seconds*
Last Click: *0 seconds*
Page Submit: *0 seconds*
Click Count: *0 clicks*

For the past five rounds: please indicate in descending order the relevance of the stakeholders for your decision.
You can drag and drop the options below to bring them in your preferred order.

Investors
Premium payers
Own role
Negotiation partner

This work is licensed under a Creative Commons Attribution 4.0 International License. Survey created with Qualtrics.

Patient

These page timer metrics will not be displayed to the recipient.
First Click: *0 seconds*
Last Click: *0 seconds*
Page Submit: *0 seconds*
Click Count: *0 clicks*

Powered by Qualtrics

This work is licensed under a Creative Commons Attribution 4.0 International License. Survey created with Qualtrics.

Social Preferences in Reimbursement Negotiations, Additional file 4 Wettstein D., Boes S. 2021

This work is licensed under a Creative Commons Attribution 4.0 International License. Survey created with Qualtrics.
